# A Turn-On Fluorescent Amino Acid Sensor Reveals Chloroquine’s Effect on Cellular Amino Acids via Inhibiting Cathepsin L

**DOI:** 10.1021/acscentsci.2c01325

**Published:** 2023-04-24

**Authors:** Michael R. Smith, Le Zhang, Yizhen Jin, Min Yang, Anusha Bade, Kevin D. Gillis, Sadhan Jana, Ramesh Naidu Bypaneni, Timothy E. Glass, Hening Lin

**Affiliations:** † Department of Chemistry and Chemical Biology, Cornell University, Ithaca, New York 14853, United States; ‡ Department of Chemistry, University of Missouri, Columbia, Missouri 65211, United States; § Graduate Program of Biochemistry, Molecular and Cell Biology, Department of Chemistry and Chemical Biology, Cornell University, Ithaca, New York 14853, United States; ∥ Howard Hughes Medical Institute, Department of Chemistry and Chemical Biology, Cornell University, Ithaca, New York 14853, United States; ⊥ Dalton Cardiovascular Research Center, Department of Bioengineering and Department of Medical Pharmacology and Physiology, University of Missouri, Columbia, Missouri 65211, United States

## Abstract

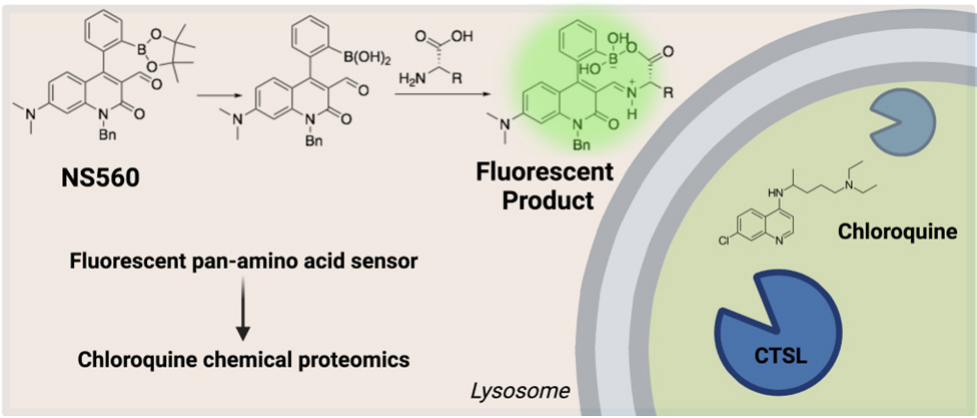

Maintaining homeostasis of metabolites such as amino acids is critical for cell survival. Dysfunction of nutrient balance can result in human diseases such as diabetes. Much remains to be discovered about how cells transport, store, and utilize amino acids due to limited research tools. Here we developed a novel, pan-amino acid fluorescent turn-on sensor, NS560. It detects 18 of the 20 proteogenic amino acids and can be visualized in mammalian cells. Using NS560, we identified amino acids pools in lysosomes, late endosomes, and surrounding the rough endoplasmic reticulum. Interestingly, we observed amino acid accumulation in large cellular foci after treatment with chloroquine, but not with other autophagy inhibitors. Using a biotinylated photo-cross-linking chloroquine analog and chemical proteomics, we identified Cathepsin L (CTSL) as the chloroquine target leading to the amino acid accumulation phenotype. This study establishes NS560 as a useful tool to study amino acid regulation, identifies new mechanisms of action of chloroquine, and demonstrates the importance of CTSL regulation of lysosomes.

## Abstract 2

Novel pan-amino acid sensor probe unveils amino acid mis-regulation after chloroquine treatment. Chemical proteomics identifies Cathepsin L as a chloroquine protein target that regulates amino acids accumulation in the lysosomes.

## Introduction

Maintenance of amino acid homeostasis is important for cellular function.^1^ In order to accomplish this task, cells have evolved different signaling and regulatory mechanisms, such as the mTOR and GCN2 signaling pathways, to sense and regulate the uptake and utilization of amino acids.^1−6^ It is established that under stress conditions such as amino acid deprivation, mTORC1 activity is downregulated, translation is inhibited, and cells adapt via a host of stress-response mechanisms.^2,3,7,8^ Dysregulation of amino acid homeostasis can lead to human diseases. For example, branched chain amino acids are critical switches in Maple syrup syndrome, mental retardation, and premature death if catabolism is dysregulated.^9^ As an amino acid sensing hub, mTORC1 has long been implicated in cancer and neurodegenerative diseases.^4,10^ These diseases highlight the importance of understanding the uptake, storage, utilization, and regulation of amino acids.

Lysosomes are critical amino acid sensing and storage depots and are responsible for degradation of autophagosomes, mitochondria, and other damaged organelles.^7^ Autophagy inhibitors, such as Bafilomycin A1, chloroquine, and ammonium chloride, are known to disrupt these functions.^11^ Bafilomycin A1 (BafA1) inhibits the vacuolar (H^+^)-ATPase critical for maintaining low pH in lysosomes and late endosomes.^12^ Chloroquine (CQ), an antimalarial drug, and ammonium chloride (NH_4_Cl) are proposed to cross lysosomal membrane where they become protonated and accumulate.^12,13^ This temporarily neutralizes lysosomes (H^+^ “sponge” effect) and renders them dysfunctional. Despite widespread use as a tool to inhibit autophagy, as well as in clinical trials for cancer and SARS-CoV-2 therapies,^14−16^ the mechanism(s) of action of chloroquine is still not well understood.

To study the uptake, storage, and utilization of amino acids, it would be useful to be able to visualize and monitor amino acid pools in live cells at high resolution. There are a number of genetically encoded biosensors available that utilize fluorescence resonance energy transfer (FRET) or fluorescent protein permutations to monitor individual metabolites.^17^ One system, named OLIVe (optical biosensor for leucine, isoleucine, and valine), uses YFP/CFP FRET technology to sense branched chain amino acids. This method is effective for branched chain amino acids but does not detect amino acids in individual organelles due to low fluorescent turn-on.^18^ Many studies have focused on glutamate sensors to study synaptic transmission in neurons but also lack resolution to determine organelle localization.^19^ Histidine or cysteine sensing follows a similar pattern.^20,21^ These methods are limited by localization of an overexpressed sensing domain and low relative turn-on signal due to the ratiometric analysis. Enriching and purifying lysosomes for eventual metabolomics (Lyso-IP) is a quantitative method used to reveal the interplay between SLC38A9 transporter activity and mTOR signaling at lysosomes, but it is not a microscopy tool for live cells.^7^ We envision that a membrane-permeable small molecule that exhibits robust turn-on fluorescence upon binding amino acids would be simple to use and enable investigations on the regulation of amino acids in cells.

A number of small-molecule fluorescent sensors for amino acids have been developed over the years, though few function well under physiological conditions except for cysteine.^22,23^ Here we report a novel fluorescent turn-on sensor for amino acids, NS560, based on our neurosensor class of fluorescent sensors.^24^ We demonstrate that NS560 can detect amino acids in live cells and confirm that lysosomes and late endosomes house free amino acid pools in cells. With this amino acid sensor, we were able to quickly test a number of small molecules for their ability to affect cellular amino acids utilization. Interestingly, chloroquine causes dramatic changes in cellular amino acids. Using a functionalized chloroquine analog for chemical proteomics, we discovered that chloroquine has affinity toward lysosomal proteins Cathepsin L (CTSL), NPC2, and PSAP. We found that chloroquine can bind and inhibit CTSL. The robust change in amino acid labeling happens mainly due to the inhibition of CTSL by chloroquine as knockdown or inhibition of CTSL led to similar NS560 amino acids labeling. Our work establishes NS560 as a useful tool for rapid imaging of free amino acids in live cells and identifies CTSL as a new target for chloroquine, which provide important insights into the various reported biological activities of chloroquine.

## Results

### Design, Synthesis, and Characterization of a Pan-Amino Acid Biosensor, NS560

**Figure 1:**
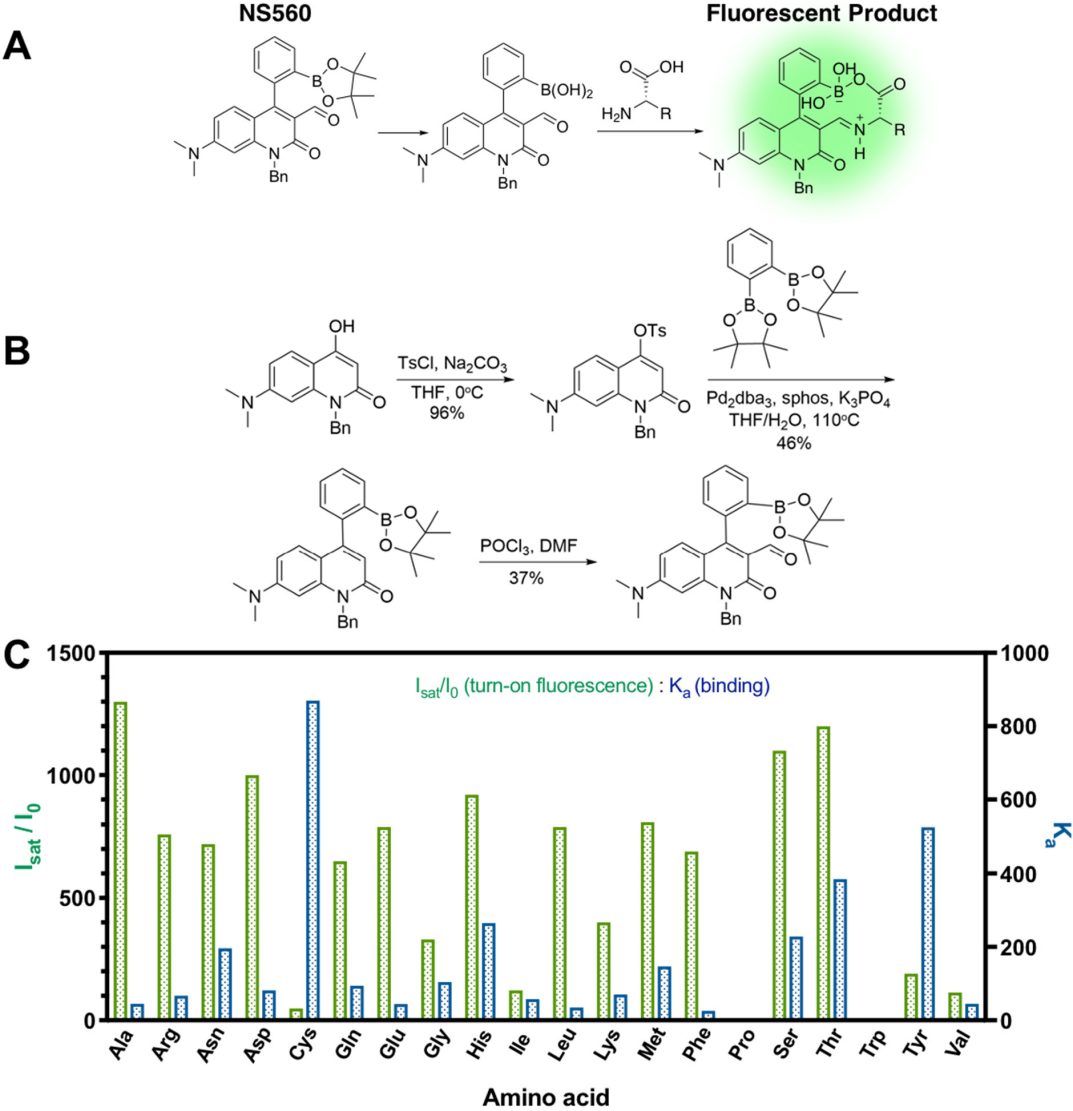
Design and synthesis of NS560 as a pan-specific amino acid probe. (A) Incubation of NS560 with free amino acids results in reversible covalent attachment of the N and C termini to the aldehyde and boronic acid, respectively, producing a fluorescent adduct. (B) Synthesis of NS560. (C) In vitro incubation of NS560 with proteinogenic amino acids leads to metabolite binding and fluorescence increase. Fluorescence enhancement was determined by taking the ratio of saturated over control fluorescence signal (10 μM NS560, 25 mM HEPES, 50 mM Na_2_S_2_O_3_, 1% DMSO, pH 7.4, λ_ex_ = 488 nm, λ_em_ = 560 nm). The fluorescence of Tyr was estimated due to low solubility.

NS560 is a quinolone fluorophore with an aldehyde and a boronic acid functional group that can react with amino acids. Phenylboronic pinacol esters are susceptible to hydrolysis at physiological pH.^25^ After reacting with amino acids, the aldehyde is converted to an iminium ion, which has a long wavelength absorption allowing selective excitation and fluorescence upon binding (Figure 1A). While isolated carboxylate groups typically do not form boronate esters favorably, proximity promotes ester formation for NS560. Formation of the macrocycle restricts the rotation of the arylboronic acid which affects the fluorescence properties of the molecule (Figure 1).^26^ NS560 was synthesized in three steps in overall 16% yield (Figure 1B, see SI Synthetic Procedures).

NS560 was tested as a pan-amino acid probe in vitro by titration with amino acids. Measurements of both absorbance and emission were collected for all 20 proteogenic amino acids. For example, upon titration with glutamate, NS560 gave a 50 nm red shift of the maximum absorbance and a ∼800-fold fluorescence enhancement at 560 nm using excitation at 488 nm (Figure S2). Although the red shift in absorbance is similar to other sensors in this class, the very high fluorescence enhancement was unprecedented. The apparent association constant of NS560 for glutamate was 44 M^–1^, which is only moderate for these types of sensors. GABA, a gamma-amino acid, also binds with moderate affinity but with much lower fluorescence enhancement and altered maximum emission wavelength (Figure S4). Thus, binding of both functional groups to the sensor is essential for the extremely high fluorescence enhancements seen with α-amino acids. Mass spectroscopic analysis of a reaction sample supports the iminium ion adduct (Figure S1). The fluorescence enhancement, indicated by the ratio of fluorescence emission of NS560 with or without amino acid ligands, was greater than 35-fold for all but proline and tryptophan (Figures 1C and S2–42, Table S1). The lack of enhancement with proline is due to the terminal secondary amine, reducing the nucleophilic attack on the aldehyde recognition element. Tryptophan’s indole ring quenches the fluorescence after reacting with NS560 similar to catecholamine with previous probes.^24^ Thus, NS560 is a pan-specific amino acid probe in vitro capable of recognizing 18 of the 20 proteogenic amino acids.

### NS560 Labels Amino Acids in Mammalian Cells

**Figure 2:**
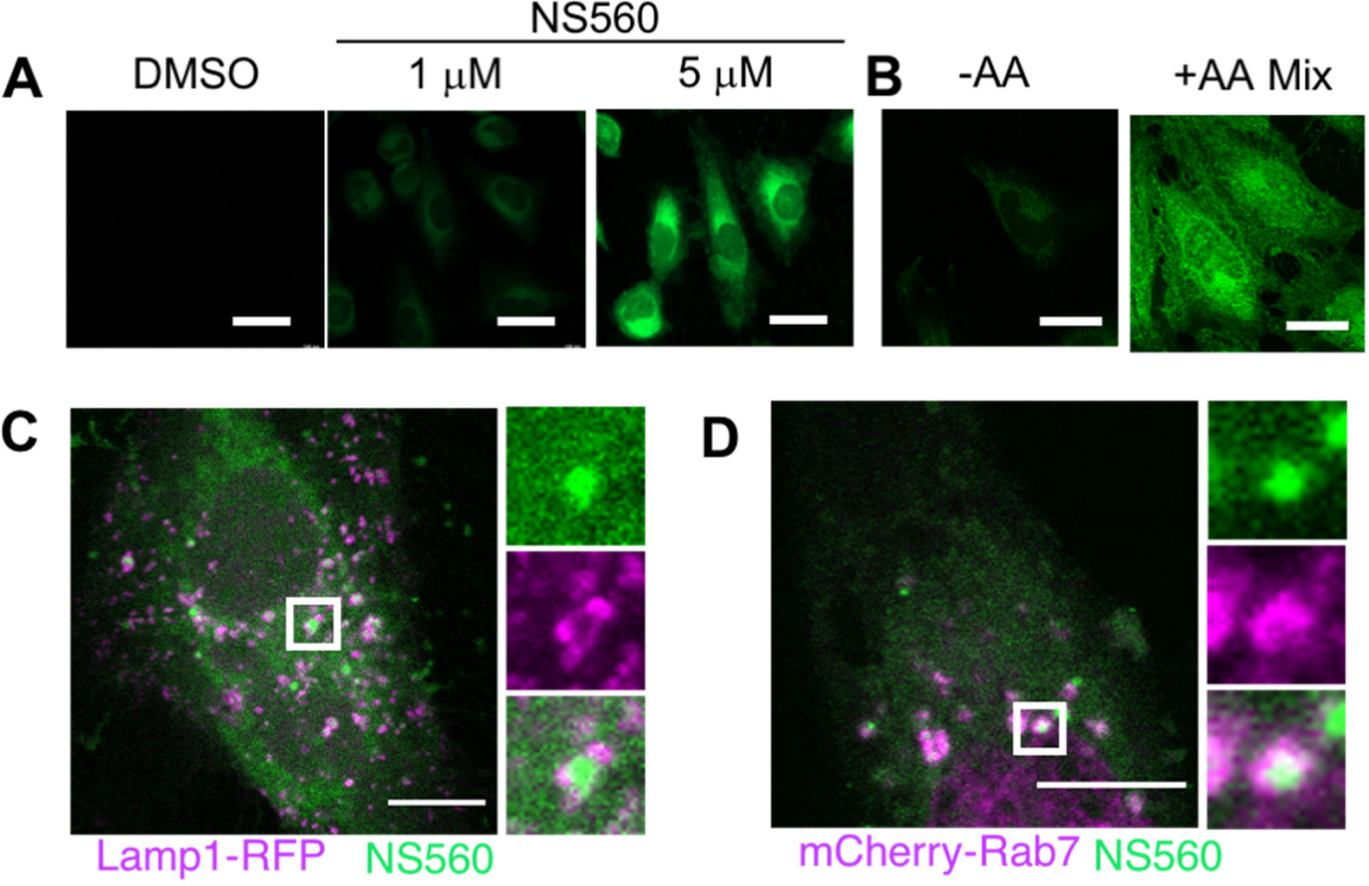
NS560 labels amino acids in HeLa cells. (A) Incubation of NS560 in HeLa cells for 45 min resulted in strong fluorescence at 5 μM. Scale bars, 40 μm. (B) Amino acid deprived HeLa cells incubated with NS560 showed increased labeling after replenishment with 5× essential amino acid solution. Scale bars, 30 μm. Representative images of three replicates. (C–D) Confocal microscopy reveals that NS560 signal can be surrounded by lysosomal marker Lamp1-RFP or dsRed-Rab7 under basal conditions. Scale bar, 5 μm. Representative images of three biological replicates.

We next tested whether NS560 can detect amino acids in live cells. HeLa cells were incubated with NS560 for 45 min, and after washing to remove probe from the cell media, the cells were examined using fluorescence microscopy. Fluorescence signal was detected at different NS560 concentrations, and 5 μM was chosen for future experiments (Figure 2A). To ensure that NS560 fluorescence was indeed from detecting amino acids, cells were briefly starved of amino acids with EBSS buffer for 60 min, incubated with NS560 for 30 min, and then replenished with a 5-fold excess of essential amino acids (compared to standard DMEM) for 30 min. NS560 signal was greatly enhanced in cells replenished with amino acids compared with control cells (Figure 2B), suggesting that NS560 could detect amino acids in cells.

### Lysosomes and Late Endosomes Are Primary Storage Compartments for Free Amino Acids

Cells in basal conditions displayed mostly diffusive amino acids with occasional small punctate structures. We hypothesized these represented organelle-specific free amino acid pools. Because lysosomes are known to store amino acids, we overexpressed fluorescent markers of the lysosome and late endosomes, Lamp1-RFP and mCherry-Rab7, respectively, and analyzed the localization of these markers with NS560 signal using confocal microscopy. Both Lamp1-RFP and mCherry-Rab7 partially colocalize or surround NS560 signal in larger puncta (Figure 2C–D). We also observed NS560 signal near rough-ER (marked with mCherry-Sec61) (Figure S45A). Rough endoplasmic reticulum (ER) is the site where protein translation occurs and may need to have amino acids readily available. Other organelle markers such as dsRed2-Rab5A (early endosome) or mCherry-Rab11A (recycling endosome) did not overlap with NS560 signal (Figure S45B–C). Given that lysosomes, late endosomes, and rough ER are predictable locations for free amino acids in cells, the data further support that NS560 can detect free amino acids in cells.

### Chloroquine Treatment Accumulates Amino Acids in Late Endosomes and Lysosomes

**Figure 3:**
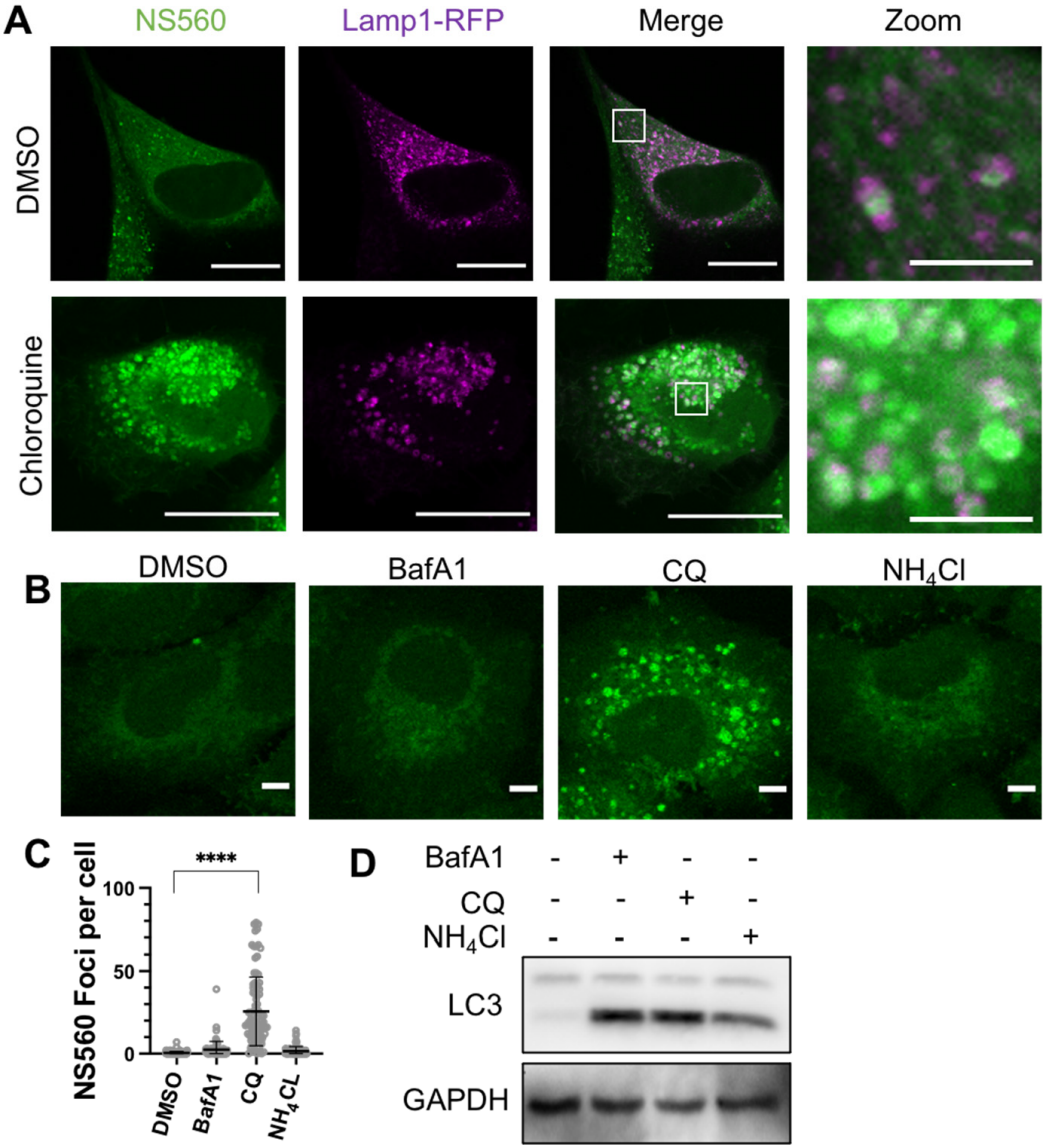
Chloroquine treatment alters amino acid pools in A549 cells. (A) Five-hour chloroquine treatment at 100 μM causes drastic buildup in lysosomal amino acid labeling. Scale bars, 20 or 4 μm (for zoom-in images). (B) A549 cells treated with BafA1 (25 nM), CQ (25 μM), and NH_4_Cl (25 mM) for 7 h. Representative images of three biological replicates. Scale bar, 5 μm. (C) Representative quantification of NS560 foci in cells treated with different small molecules. (D) Western blot analysis of cells treated in B to detect LC3 levels (autophagy). GAPDH was used as loading control. Representative images are from three independent biological replicates.

As NS560 allows facile detection of free amino acids in live cells, we wanted to use it to screen for small molecules that could affect the distribution of cellular amino acids. Interestingly, we found that chloroquine can dramatically alter the distribution of cellular amino acids. Incubation for either 7 or 24 h with 25 μM of chloroquine in A549 cells led to a buildup of puncta of amino acids signals that colocalized with both lysosomes and late endosomes (Figures 3A and S46). The effect was unique to chloroquine, as other lysosome inhibitors bafilomycin or ammonium chloride did not result in such a phenotype despite the fact that all three inhibitors led to LC3-II accumulation, a hallmark of autophagy blockade (Figure 3B–D). Chloroquine, originally used as an antimalarial drug, is now commonly used as an autophagy inhibitor in cells. It is generally believed that chloroquine inhibits autophagy by raising the pH of acidic organelles.^27^ However, our observation of amino acid buildup after treatment is not consistent with augmented lysosomal pH, especially given that bafilomycin and ammonium chloride do not cause amino acid buildup. In our hands and in published literature, LysoTracker Red signal increases after prolonged (24 h) chloroquine treatment, indicating more acidic lysosomal organelles.^12^ Our observation suggests chloroquine treatment accumulates amino acids in lysosomes and late endosomes via mechanisms other than affecting lysosomal pH.

### Chemical Proteomics Identifies Chloroquine Binding Proteins in Lysosomes

**Figure 4:**
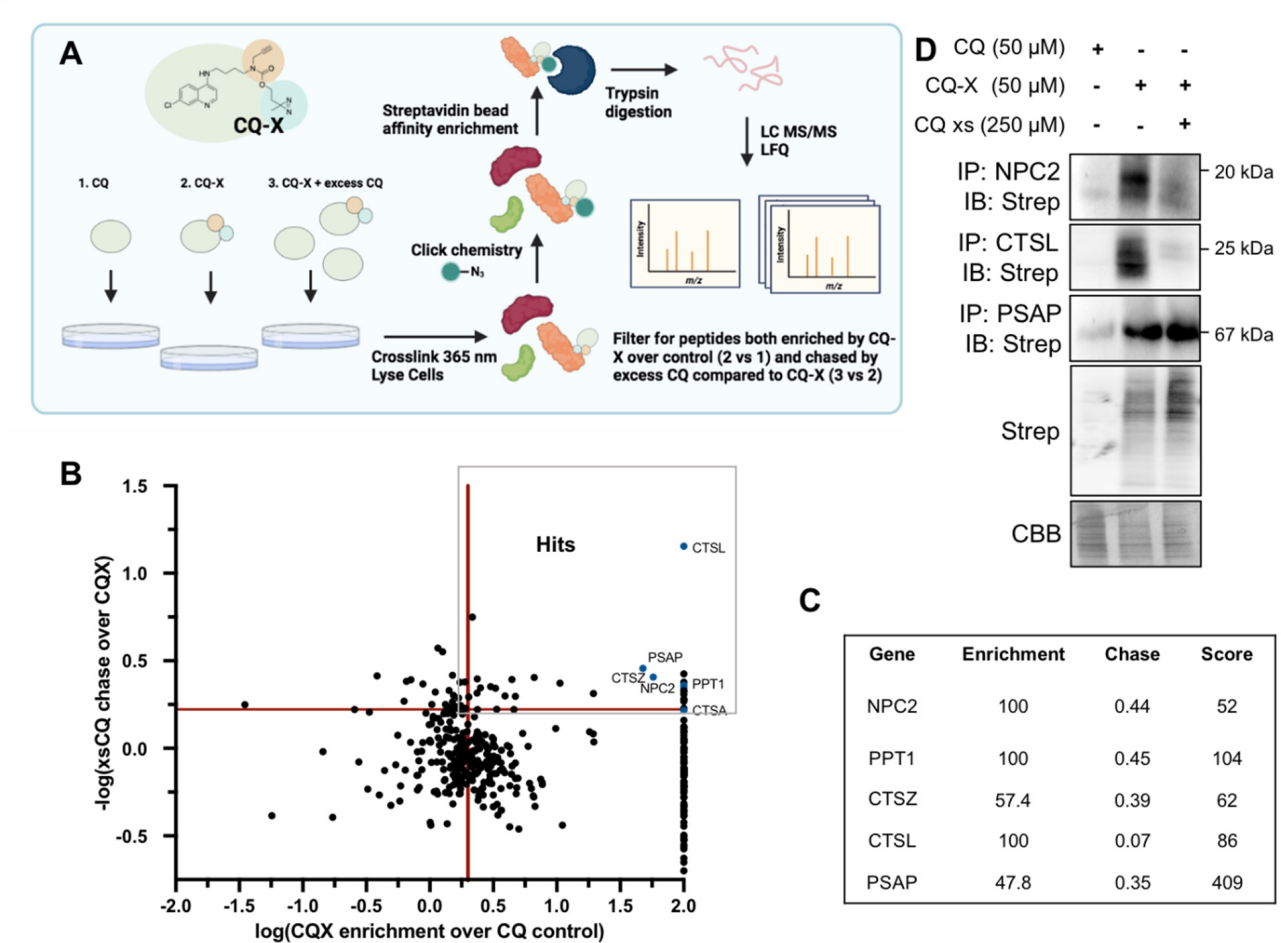
Chemical proteomics identifies lysosomal CQ targets. (A) Schematic of the proteomics strategy. Samples were prepared in triplicate for analysis. (B) Label-free proteomics results. Red bars indicate cut-offs (enrichment >2, chase rate >40%). (C) Relevant hits from proteomics data. (D) Validation of three chloroquine binding proteins from the proteomics data, CTSL, PSAP, and NPC2. CQ-X cross-linked targets were enriched via antibody and confirmed by blotting for cross-linked CQ-X via streptavidin blot. Representative Western blot for three biologically independent experiments.

We hypothesized that chloroquine binds to and inhibits certain proteins, leading to free amino acids accumulation in lysosomes. In order to test this and identify these target proteins, we synthesized a chloroquine derivative (CQ-X) with a diazirine group for UV-cross-linking and an alkyne handle for copper(I)-catalyzed azide–alkyne cycloaddition to “click” on biotin (Figure S47). Importantly, CQ-X maintains the same NS560 labeling phenotypes as the parent chloroquine compound (Figure S48A). Furthermore, treatment of cells with chloroquine or CQ-X results in LC3-II accumulation (Figure S48B). A549 cells were treated for 1 h with chloroquine (control, 50 μM), CQ-X (50 μM), or CQ-X in combination with a 5-fold excess of chloroquine (chase). Cross-linking was carried out with 365 nm light. Cells were lysed, and biotin was attached via click chemistry. Proteins labeled by CQ-X were then affinity enriched with streptavidin beads and identified by MS after on-bead trypsin digestion (Figure 4A). We used label-free quantification (LFQ) to find proteins that are more abundant (≥2-fold) in the CQ-X treated sample than in the chloroquine or CQ-X/chloroquine treated samples. 243 proteins were enriched >2-fold by CQ-X, but only 29 were both enriched and chased by excess chloroquine below a ratio of 0.6 (Figure 4B–C, Table S2). The proteomics results are reliable for several reasons. First, there is a published crystal structure of chloroquine bound to saposin B (PSAP is a precursor for saposin A through saposin D and is identified as a chloroquine target in our proteomics study).^28^ Second, palmitoyl-protein thioesterase 1 (PPT1) is reported to bind dimeric chloroquine derivatives^29^ and is identified as a chloroquine target in our proteomics study. Lastly, many of the identified proteins exist in the lysosomes, which is consistent with chloroquine being a lysosomotropic agent.

We confirmed the proteomics results by cross-linking with CQ-X and pulling down several protein hits identified, NPC2, CTSL, and PSAP, and then immunoblotted for streptavidin signal. We confirmed that all three targets were bound by CQ-X, but only NPC2 and CTSL were chased by excess chloroquine (Figure 4D). The results confirmed that chloroquine could bind to NPC2 and CTSL.

### Knockdown or Inhibition of CQ-X Binding Proteins Alters Free Amino Acids

**Figure 5:**
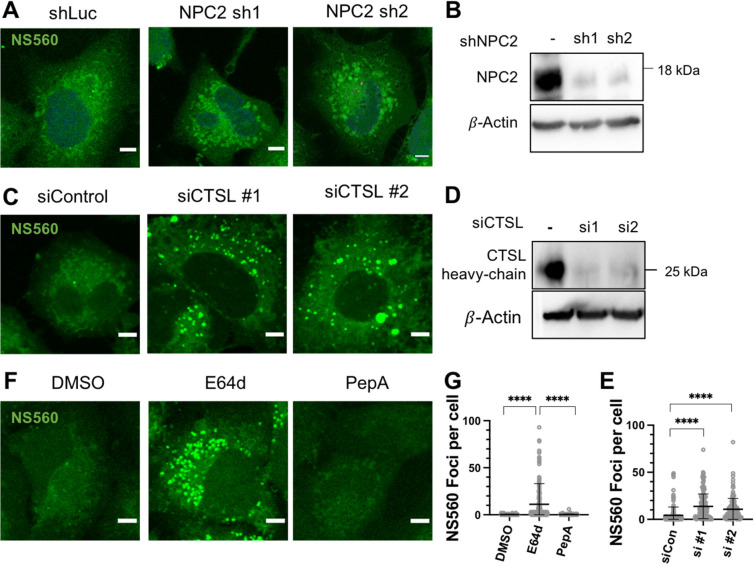
Chloroquine targets CTSL and NPC2 to regulate free amino acids. A549 cells were treated with shRNA for NPC2 for 48 h (A) or transfected with siRNA for CTSL for 48−72 h (C) then labeled with NS560 for 45 min. Representative Western blots from three biological replicates show efficient knockdown of all genes (B and D). (E) Representative quantification of NS560 foci per cell for images shown in C. (F) A549 cells treated with E64d (25 μM) or Pepstatin A (25 μM) for 24 h then labeled with NS560. Scale bar, 5 μm for all images. (G) Representative quantification of NS560 foci per cell for images shown in (F).

In order to connect the CQ-X proteomics results with chloroquine induced cellular amino acids distribution, we used shRNA or siRNA to knockdown the identified chloroquine target proteins, NPC2 and CTSL. Knockdown of NPC2 resulted in larger fluorescent amino acid sites (Figure 5A–B), but the NS560 signal was not drastically increased. In contrast, siRNA knockdown of CTSL resulted in a quantifiable increase in NS560 foci compared to control (Figure 5C–E). The siRNA knockdown of CTSL efficiently reduced CTSL as detected via Western blot. Because multiple cathepsins (A, L, and Z) were identified in the proteomics study, the chloroquine effect on cathepsin may not be specific to CTSL. We treated A549 cells with an irreversible pan-cysteine protease inhibitor, E64d, or an aspartyl protease inhibitor Pepstatin A. After NS560 labeling, E64d treated cells showed an obvious free amino acid buildup in NS560 foci, while Pepstatin A had no effect (Figure 5F–G). This suggests cysteine proteases (Cathepsins L, Z, or others) but not aspartyl proteases (Cathepsins D and E) regulate free amino acid pools. Overall, genetic and chemical manipulation of CTSL inhibition could lead to changes in amino acids distribution.

### Chloroquine Inhibits Cathepsin L In Vitro

**Figure 6:**
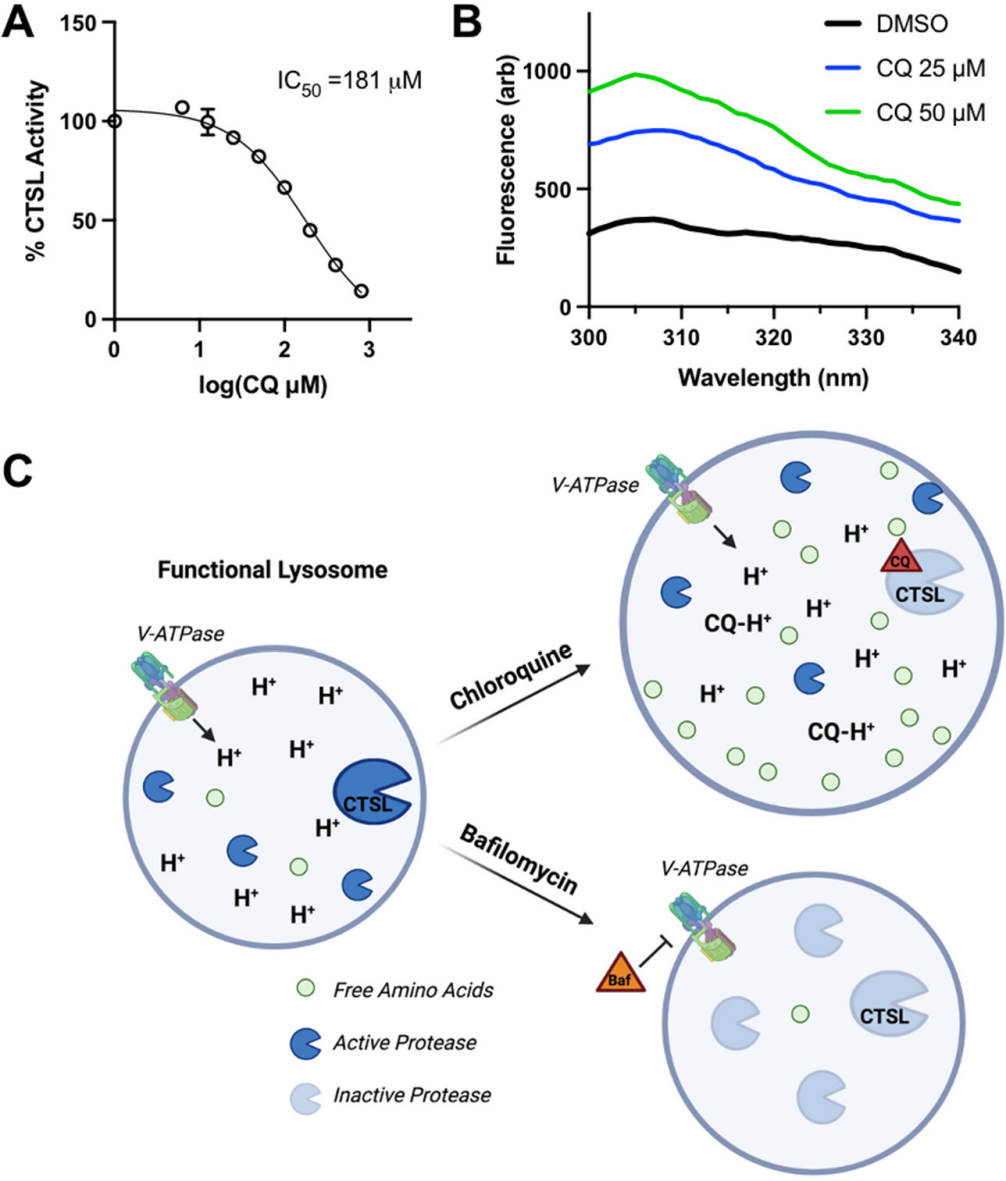
Chloroquine inhibits CTSL activity and increases intrinsic fluorescence in vitro. (A) CTSL hydrolysis of dipeptide substrate Z-FR-AMC was monitored by measuring the fluorescence of the released AMC. Reaction was performed at pH 5.5 in 100 mM MES-NaOH, 150 mM NaCl, and 7.5 mM DTT for 30 min after 25 min preincubation with chloroquine (CQ). Representative plot and calculation of three biological replicates. (B) Intrinsic fluorescence of purified CTSL-Flag, 200 nM, incubated with and without chloroquine. Representative data from three independent experiments. Fluorescence values were corrected for chloroquine background at these wavelengths. (C) Schematic representation of chloroquine-induced lysosome amino acid accumulation as compared with Bafilomycin treatment.

Next, we focused on the specific effect of chloroquine on CTSL. First, we tested if chloroquine can inhibit CTSL in vitro using commercially available purified enzyme and well-established dipeptide substrate Z-Phe-Arg-AMC. Active CTSL will cleave AMC and increase the fluorescence. After 25 min chloroquine preincubation with CTSL enzyme, AMC cleavage was inhibited by chloroquine with an IC_50_ of 181 μM (Figure 6A). While the IC_50_ seems to be on the high range, we believe this is physiologically relevant as chloroquine is a lysosomotropic drug and is estimated to reach concentrations of >25 mM in the lysosomes.^13^

Seeking further validation of chloroquine binding CTSL, we overexpressed a CTSL-Flag-Myc plasmid in HEK-293T cells and purified the enzyme using Flag beads (Figure S55). We measured chloroquine binding to CTSL by measuring the changes in the intrinsic fluorescence of CTSL by different concentrations of chloroquine. Exciting CTSL at 265 nm results in an emission peak around 308 nm. The presence of chloroquine caused an increase in the fluorescence, indicating the binding of chloroquine to CTSL (Figure 6B).

To further validate the direct binding of chloroquine to CTSL, we designed a chloroquine-TAMRA derivate (CQ-TAMRA) and performed a fluorescence polarization assay by incubating this new probe with varying concentrations of CTSL. A mild milipolarization (mP) shift of 20–25 was obtained at maximum CTSL concentrations indicating mild binding (Figure S56). The data again support that chloroquine binds to CTSL. However, due to the limited CTSL we could obtain, we could not saturate the binding to get a binding constant.

Using computational modeling, chloroquine can be docked to CTSL near its catalytic triad (Figure S51). This suggests that CQ is a competitive inhibitor. Based on this assumption and the IC_50_ value we obtained as well as the reported *K*_*m*_ value of the Z-Phe-Arg-AMC substrate,^30^ we can calculate a dissociation constant of ∼35 μM using the equation of *K*_*d*_ = IC_50_/(1 + [S]/*K*_*m*_).

Overall, our study shows that CTSL is an important protein for regulating free amino acids in cells. Prolonged chloroquine or E64d treatment inhibits lysosomal CTSL, but the degradative capacity of the lysosome remains largely intact likely due to the presence of other proteases. However, the resulting degradation products (free amino acids) are not efficiently exported due to the inhibition of CTSL by chloroquine or E64d (Figure 6C). In contrast, bafilomycin, which increases the lysosomal pH and thus inhibits all the lysosomal proteases, leads to diminished protein degradation and thus did not lead to amino acids accumulation in the lysosome.

## Discussion

Our study introduces a novel chemical probe, NS560, that can be utilized to uncover important cell biology related to amino acid storage, utilization, and regulation. The probe rapidly labels free amino acid pools in cells and thus can provide important information about the levels and localizations of total amino acids.

Using NS560, we screened several small molecules to see whether any of them could alter cellular amino acid levels and/or localization. This led to the finding that chloroquine treatment causes previously unknown accumulation of amino acids in specific organelles in cells. Further chemical proteomics and biochemical studies established that this effect is due to chloroquine binding to and inhibiting lysosomal proteins CTSL (and possibly other cathepsins cysteine proteases). The study of chloroquine’s effect on cellular amino acid distribution is a nice example demonstrating the utility of NS560. Amino acids are essential for cellular life and thus have to be carefully regulated. The ability to visualize amino acids would enable us to track changes in amino acid levels and localization in a variety of conditions, such as metabolic stress, signaling activation, or disease conditions, which could provide important insights to understand fundamental cellular processes.

Since its discovery as a potent antimalarial agent, chloroquine’s mechanism of action has been intensely studied. Here we observe extended (7 h or longer) chloroquine treatment results in large amino acid foci (Figure 4A). Whether these pools of amino acids are a result of increased uptake, increased protein degradation, or decreased export requires further research. There is a dichotomy in the literature pertaining to pH and hydrolytic capacity of lysosomes after chloroquine treatment. Nobel laureate Christian de Duve wrote a commentary discussing lysosomotropism that highlights amine-containing, weak base compounds hyper-accumulate in lysosomes due to pH partitioning.^13,27,31,32^ Upon protonation, agents such as chloroquine become trapped, resulting in commonly cited increased organelle pH that inactivates hydrolytic enzymes.^32−34^ In stark contrast, there are reports showing that lysosomes adapt to chloroquine treatment and increase lysosomal acidity but develop other dysfunctions.^32^ Another report emphasizes that chloroquine inhibits the autophagosome-lysosome fusion step of autophagy and severely alters Golgi and endolysosomal systems but does not increase pH.^12^ In our hands, prolonged CQ treatment results in enlarged lysosomes, recovered acidic pH, and accumulation of amino acids.

We employed commonly used autophagy inhibitors Bafilomycin A1 and NH_4_Cl to compare to chloroquine. Bafilomycin A1 inhibits the lysosomal proton pump to raise lysosomal pH, while NH_4_Cl is another lysosomotropic compound. In our hands, chloroquine or NH_4_Cl treatment for 1 h completely eliminates Lysotracker signal, consistent with predictions from the initial lysosomotropism analysis.^13^ However, 7 h after addition of either lysosomotropic autophagy inhibitor, Lysotracker signal returns due to previously reported cellular adaptation.^12,32^ Amino acid accumulation begins during this adaptation time period, but the drastic phenotype is only present in chloroquine-treated cells but not in bafilomycin A1 or NH_4_Cl-treated cells. Thus, we believe the amino acid buildup after chloroquine treatment is not a result of raised pH or inactive lysosomes.

Interestingly, several reports are consistent with our findings that chloroquine (but not BafA1 or NH_4_Cl) leads to amino acids accumulation in the lysosomes. One study shows that after 24 h of chloroquine (but not BafA1) treatment, mTORC1 relocalizes away from lysosomes.^6^ Another shows that chloroquine (but not BafA1 or NH_4_Cl) treatment for 24 h reduces mTORC1 activity (p-S6K).^11^ It is likely that the accumulation of amino acids in the lysosomes by chloroquine leads to the decrease of amino acids in the cytosol, which are required for mTORC1 activation.

Our data also suggest CTSL plays an important role in amino acid regulation beyond degradation of proteins. Cathepsins have become increasingly studied in relation to lysosomal dysfunction in recent years. For example, Cathepsins B and L regulate NPC2 secretion in macrophages activated with LPS, which impacts cholesterol metabolism.^35^ Another study reports that inhibition or genetic deletion of Cathepsins B and L (but not D) results in lysosomes with accumulated cholesterol, LC3-II, and Lysotracker.^36^ How CTSL inhibition by chloroquine or E64d leads to amino acids accumulation requires further studies. One hypothesis is that CTSL regulates an amino acid transporter. Regardless of the exact mechanism, our findings highlight the importance of CTSL in lysosomal regulation.

CTSL has become a drug candidate for the SARS-CoV-2 pandemic because it cleaves the spike protein critical for infection.^37^ The inhibition of CTSL by chloroquine may also explain the reported potential beneficial effects of chloroquine in treating SARS-CoV-2 infection. Given that CTSL is not the major protease that cleaves the spike protein, our data are also consistent with the fact that chloroquine is not highly effective in treating SARS-CoV-2 infection in humans.^38,39^

Overall, this work highlights the usefulness of NS560 as a novel tool to visualize amino acid in cells. By employing NS560, a snapshot of the amino acid state of cells can be visualized. The use of NS560 is amenable for high-throughput screening, which will help uncover new biology related to the essential cellular building blocks, amino acids, as we show here for chloroquine.

## Methods

### General Synthetic Procedures of NS560

Chemicals were obtained from Sigma-Aldrich, Acros, Fisher, TCI America, Alfa Aesar, or Combi-Blocks and were used without further purification. Flash chromatography was performed with 32–63 μm silica gel. NMR spectra were recorded on a Bruker DRX 500 and 600. IR spectra were recorded on a Nexus 670 FT-IR E.S.P. spectrometer. Detailed synthesis and structural characterization can be found in the Supporting Information.

### Spectroscopic Studies of NS560

One mM stock solution of NS560 in DMSO for UV/vis spectra and fluorescence spectra was prepared and diluted to 1 mL with buffer (1.0 × 10^–5^ M, 25 mM HEPES, 50 mM Na_2_S_2_O_3_, pH 7.4, 5.0, 1% DMSO). The analytes (20 proteogenic amino acids and GABA) were prepared by dissolving the analytes in buffer sensor solution (the concentration of NS560 was the same as described above) to make sure the concentration of NS560 keeps constant. Sodium thiosulfate was used to protect aromatic analytes from oxidation in solution. UV/vis spectra were recorded on an Agilent Cary 100 UV/vis spectrophotometer at ambient temperature. Fluorescence spectra were recorded on a Shimadzu RF-6000 PC Spectro Fluorophotometer at ambient temperature.

### Common Reagents and Antibodies

The following reagents and antibodies were purchased from commercial sources: Antibodies against β-actin HRP (sc-4777), CTSL (sc-32320), GAPDH (sc-47724 HRP), normal mouse IgG (sc-2025), normal rabbit IgG (sc-2027) along with Protein A/G PLUS-Agarose beads (sc-2003) were purchased from Santa Cruz. Antibodies against LC3 (Cat. #12741) and Streptavidin-HRP (Cat. #3999) were purchased from Cell Signaling Technology. Antibodies against PSAP (A1819) and NPC2 (A5413) were purchased from Abclonal. Protease inhibitor cocktail was purchased from Sigma-Aldrich (Cat. P8340). Streptavidin beads, ECL Western blotting detection reagent, and Pierce Universal nuclease were purchased from ThermoScientific. ClarityMax Western blotting detection reagent was purchased from BioRad (Cat. 1705062). Polyethylenimine (PEI) was purchased from Polysciences (Cat. 4765). Inhibitors used were all purchased as follows: Bafilomycin from CST (Cat. 54645), chloroquine diphosphate from TCI (C2301), ammonium chloride from Fisher Scientific (A661), E64d from SeleckChem (S7393), and Pepstatin A from Sigma (P5318). BCA assay was used for protein concentrations. Expression plasmids for organelle markers were all purchased from Addgene: mCherry-Sec61 (#49155), dsRed-Rab5 (#13050), mCherry-Rab11 (#55124), dsRed-Rab7 (#12661), and Lamp1-RFP (#1817).^40−43^

### Cell Culture

A549, HEK-293T, and Hela cells were purchased from ATCC. A549 cells were cultured in RPMI media from Thermo (1875135) with 10% fetal bovine serum from Thermo. HeLa cells were cultured in DMEM (Gibco Cat. 11965–092) with 10% fetal bovine serum. HEK-293T cells were cultured in DMEM with 10% calf serum. For transient knockdown experiments, shRNA and siRNA were purchased from Sigma. NPC2 sh.1 (TRC0000293234), NPC2 sh.2 (TRC0000293323), CTSL si.1 (SASI_Hs01_00079400), and CTSL si.2 (SASI_Hs02_00332791). shRNA lentiviral particles were generated by cotransfecting shRNA with psPAX packaging plasmid and pMD2.G enveloping plasmid. Particles were collected, filtered with 0.25 μm sterile filter, and used for future knockdown in A549 cells.

### In-Cell Microscopy of NS560

A549 or HeLa cells were seeded on a 35 mm glass bottom poly-d-lysine coated MatTek imaging dishes (Cat. P35GC-1.5C) 1 day prior to experiment. NS560 was added to cell media at 5 μM for 45 min prior to either live cell imaging or fixing unless indicated otherwise (Figure 2A–B). At the same time as NS560 addition, Lysotracker Deep Red (ThermoFisher Cat. L12492), Magic Red L (Immunochemistry Cat. #941), and/or Hoechst 33342 nuclear stain (ThermoFisher Cat. H3570) were added to cell media as indicated. After 45 min, cells were washed twice in PBS, and images were captured by BioTek Cytation 5 microscope (Figure 2A–B). For all detailed puncta or localization experiments, cells were washed twice with PBS, fixed with 4% PFA in PBS, and mounted using Fluoromount-G (Southern Biotech Cat. # 0100-01). Cells were then imaged the same day as the probe addition using Zeiss LSM 710 confocal microscope. NS560 and LysoTracker fluorescence will decrease if imaged the following day. Detailed analysis for NS560 foci count was performed using a macro designed with ImageJ software.

### Amino Acid Deprivation and Addition Studies

A549 or HeLa cells were seeded the same as described above in standard media 1 day prior to experiment. The following day, cells were washed 3 times with EBSS salt solution (ThermoFisher Cat. #24010043) and then grown in EBSS media without amino acids but supplemented with 2 g/L glucose (Thermo Cat. #A24940-01), vitamins (100× stock from Thermo Cat. 11120052), and 10% dialyzed FBS for 30 min following protocols known to impact mTORC1.^8^ Next, NS560 (5 μM) and Hoechst stain were added and incubated for 30 more minutes. Cells were again washed with EBSS to remove NS560 from the media and then incubated with the same EBSS starvation media supplemented with or with 5X MEM essential amino acids (50× stock from ThermoFisher Cat. # 11130-051). Cells were imaged for total integral NS560 fluorescence using Cytation 5 microscope and data analysis.

### Sample Preparation for CQ-X In-Cell Cross-Linking and Click Chemistry

A549 cells were treated with chloroquine or CQ-X as indicated for 45 min. Cells were subjected to 10 min of cross-linking at 365 nm using a Boekel Scientific UV cross-linker. Next, cells were collected with cold PBS and lysed in NP-40 lysis buffer with protease inhibitor cocktail. Biotin-azide (Apex Bio Cat. A8013) was covalently attached via copper(I) catalyzed click chemistry. The reaction was run for 1 h at room temperature. Proteins were extracted with chloroform–methanol extraction methods. The protein pellet was resolubilized in buffer containing 8 M urea, 2.5% SDS, and 0.3 M NaCl. After BCA quantification, equal amounts of lysate were diluted into 0.1% NP-40 IP wash buffer and incubated with either streptavidin beads for proteomics or antibodies for chloroquine protein targets to validate proteomic results. Streptavidin beads were washed and submitted for digestion and proteomics analysis.

### On-Bead Trypsin Digestion for Proteomics Samples

The PBS storage buffer was removed from the beads. To denature and reduce the proteins bound to the beads, 30 μL of 50 mM TEAB (pH 8.5), 6 M urea, 2 M thiourea, 10 mM DTT were added and then incubated for 1 h at 35 °C. This was then followed by alkylation with 50 mM iodoacetamide for 45 min in the dark and quenched with a final concentration of 50 mM dithiothreitol (DTT). Samples were diluted with 50 mM TEAB pH 8.5 to a final concentration of 1 M urea. Trypsin was then added to a final concentration of 10 ng/μL and incubated overnight (16 h) at 35 °C. The digested peptides were desalted with Oasis MCX cartridge (Waters) and then dried down to ∼100 μL using speed vacuum SC110 (Thermo Savant, Milford, MA). All samples were filtered with 0.22 μm cellulose acetate spin filters (Costar). Filtered peptides were then dried down to dryness in the speed vacuum.

### Protein Identification by Nano LC/MS/MS Analysis

The tryptic digests were reconstituted in 2% acetonitrile (ACN) containing 0.5% formic acid (FA), and enolase (yeast) tryptic digest was added to the final concentration of 100 fmol/μL as internal standard for nanoLC-ESI-MS/MS analysis. The analysis was carried out using an Orbitrap Eclipse Tribrid (Thermo-Fisher Scientific, San Jose, CA) mass spectrometer equipped with a nanospray Flex Ion Source and coupled with a Dionex UltiMate 3000 RSLCnano system (Thermo, Sunnyvale, CA). The peptide samples (10 μL) were injected onto a PepMap C-18 RP nano trapping column (5 μm, 100 μm i.d × 20 mm) at 20 μL/min flow rate for rapid sample loading and then separated on a PepMap C-18 RP nano column (2 μm, 75 μm × 25 cm) at 35 °C. The tryptic peptides were eluted in a 90 min gradient of 5% to 35% ACN in 0.1% formic acid at 300 nL/min, followed by 8 min ramping to 90% ACN-0.1% FA and a 7 min hold at 90% ACN-0.1% FA. The column was re-equilibrated with 0.1% FA for 25 min prior to the next run. The Orbitrap Eclipse was operated in positive ion mode with spray voltage set at 1.6 kV and source temperature at 300 °C. External calibration for FT, IT, and quadrupole mass analyzers was performed. In data-dependent acquisition (DDA) analysis, the instrument was operated using FT mass analyzer in MS scan to select precursor ions followed by 3 s “Top-Speed” data-dependent CID ion trap MS/MS scans at 1.6 *m*/*z* quadrupole isolation for precursor peptides with multiple charged ions above a threshold ion count of 10,000 and normalized collision energy of 30%. MS survey scans were at a resolving power of 120,000 (fwhm at *m*/*z* 200) for the mass range of *m*/*z* 375–1575. Dynamic exclusion parameters were set at 50 s of exclusion duration with ±10 ppm exclusion mass width. All data were acquired under Xcalibur 4.4 operation software (Thermo-Fisher Scientific).

### Data Analysis

The DDA raw files for CID MS/MS were subjected to database searches using Proteome Discoverer (PD) 2.5 software (Thermo Fisher Scientific, Bremen, Germany) with the Sequest HT algorithm. Processing workflow for precursor-based quantification. The PD 2.5 processing workflow containing an additional node of Minora Feature Detector for precursor ion-based quantification was used for protein identification and protein relatively quantitation analysis between samples. The database search was conducted against a *Homo sapiens* NCBI database that has 81,786 sequences. Two-missed trypsin cleavage sites were allowed. The peptide precursor tolerance was set to 10 ppm, and fragment ion tolerance was set to 0.6 Da. Variable modification of methionine oxidation, deamidation of asparagines/glutamine, acetylation, M-loss, and M-loss+acetylation on protein N-terminus and fixed modification of cysteine carbamidomethylation were set for the database search. Only high confidence peptides defined by Sequest HT with a 1% FDR by Percolator were considered for the peptide identification.

Relative quantitation of identified proteins between the control and treated samples was determined by the Label Free Quantitation (LFQ) workflow in PD 2.5. The precursor abundance intensities for each peptide identified by MS/MS in each sample were automatically determined, and their unique peptides for each protein in each sample were summed and used for calculating the protein abundance by PD 2.5 software with normalization against the spike yeast enolase protein. Protein ratios were calculated based on pairwise ratio for treatment over control samples.

### CTSL Activity Assay Using Z-FR-AMC

A Cytation 5 (BioTek) plate-reader was used to analyze CTSL enzymatic activity against a dipeptide substrate Z-FR-AMC (R&D Systems ES009). CTSL for this experiment was purchased from BPS Biosciences as part of the Cathepsin L Inhibitor screening assay kit (Cat. # 79591). The kit buffer was substituted with a similar buffer: 100 mM MES-NaOH pH 5.5, 7.5 mM DTT, and 150 mM NaCl. 2-Fold serial dilutions of CQ were preincubated with CTSL for 25 min on ice. The final enzyme concentration was 0.016 ng/μL, the final substrate concentration was 5 μM, and the reaction was run at 25 °C for 30 min. Three technical replicates were run for each reaction, and the experiment was performed three times. AMC fluorescence (substrate cleavage) was monitored by excitation at 340 nm and emission at 445 nm (F_445_). Proper controls were run for all components, as CQ fluorescence has potential to impact fluorescence spectra. Percent activity was calculated by comparing F_445_ for DMSO treated CTSL to the varying CQ concentrations. IC_50_ was calculated using Graphpad Prism analysis using log(inhibitor) vs response nonlinear regression analysis.

### Purification of CTSL

CTSL-Flag-Myc mammalian expression vector was purchased from Origene (Cat. # RC203143). The plasmid was expressed in six 15 cm plates of HEK-293T cells (ATCC). Cells were collected and lysed in 1%-NP-40 lysis buffer. Importantly, protease inhibitors were not used in the lysis process. Instead, all steps were performed as efficiently as possible on ice. Lysates were enriched with Flag-beads for 2 h (Sigma Cat. # A2220), washed 3 times in 0.1% NP-40 wash buffer and then 2 more times in 50 mM Tris, 150 mM NaCl buffer. Flag-beads were eluted with 180 μM Flag-peptide (Biomatik) and concentrated, and total protein was quantified via Bradford assay. An aliquot of purified protein was confirmed to be CTSL via Coomassie stained SDS-page gel and Western blot for Flag signal.

### CTSL Intrinsic Fluorescence

Purified CTSL-Flag intrinsic fluorescence was monitored using a Cytation 5 plate reading instrument. 200 nM of protein was added to the same, chilled, CTSL activity assay buffer (see above) and kept in a clear-bottom 96-well UV-star plate (Greiner Cat. # 655809). Exciting the protein at 265 nm and monitoring emission from 300 nm to beyond 400 nm showed a peak above the buffer background around 308 nm. 10 min preincubation of increasing CQ concentrations caused an increase in fluorescence at this peak. At concentrations of 25 and 50 μM (Figure 6B), CQ alone has lower fluorescence than buffer control at 308 nm. Thus, the data are presented with CQ background subtraction to best represent fluorescence increase of CTSL with CQ treatment.

### Chloroquine Computational Modeling

A reference CTSL structure was obtained from PDB database (2XU3) and loaded into MOE (2020) software as Biomolecule Assembly with default settings.^44^ The protein structure was then prepared using the QuickPrep function with the default parameters and thoroughly checked using the Structure Preparation function. The potential binding sites of CTSL were calculated and identified using the Site Finder tool. CQ was then docked into the identified site, and the docking poses were scored using London dG and refined based on GBVI/WSA dG. The most confident binding pose was selected and visualized in MOE software.

### Fluorescence Polarization Assay

The stock solution of purified CTSL was diluted with mixture of 10× assay buffer (final concentrations of 25 mM Tris pH 8.0, 150 mM NaCl and 0.01% Tween-20), CQ-TAMRA (150 nM) and water to a total volume of 50 uL in Corning 96-well, half-area black plates. The plate was covered and left on ice for 10 min. Two technical replicates per sample type were measured. The plate was scanned 3 times on Cytation5 using a FP filter cube (Agilent, part number: 8040562, Ex: 530/25, Em: 590/35). The parallel and perpendicular fluorescence intensities of each well were recorded, and the mP values were then calculated based on the blank-subtracted data using established formula.^45^

## Supporting Information Available

Figures S1–S56, Table S1, and synthetic procedures for NS560 and CQ-X (PDF)
Additional data (XLSX)
Transparent Peer Review report available (PDF)
