# Supplementary material for: A Turn-On Fluorescent Amino Acid Sensor Reveals Chloroquine’s Effect on Cellular Amino Acids via Inhibiting Cathepsin L
Source: ACS Cent Sci. 2023 Apr 24;9(5):980–91. doi: 10.1021/acscentsci.2c01325 (PMC10214525; doi:10.1021/acscentsci.2c01325)

Supporting information for

A turn-on fluorescent amino acid sensor reveals chloroquine's effect on cellular amino acids via inhibiting cathepsin L

Michael R. Smith[1,#], Le Zhang[2, #], Yizhen Jin[3], Min Yang[1], Anusha Bade,[2] Kevin D. Gillis[5], Sadhan Jana[1], Ramesh N. Bypaneni[2], Timothy E. Glass[2,*], and Hening Lin[1,4,*]

[1]Department of Chemistry and Chemical Biology, Cornell University, Ithaca, NY 14853, USA.

[2]Department of Chemistry, University of Missouri, Columbia, Missouri, 65211, USA

[3] Graduate Program of Biochemistry, Molecular and Cell Biology; Department of Chemistry and Chemical Biology, Cornell University, Ithaca, NY 14853, USA

[4]Howard Hughes Medical Institute; Department of Chemistry and Chemical Biology, Cornell University, Ithaca, NY 14853, USA

[5]Dalton Cardiovascular Research Center, Department of Bioengineering and Department of Medical Pharmacology and Physiology, University of Missouri, Columbia, Missouri, 65211, USA

#Equal contribution

*Correspondence to: Timothy Glass (glasst@missouri.edu) and Hening Lin (hl379@cornell.edu)

This file includes:

Supporting Information Figures S1 to S55

Supporting Information Table S1

Supporting Information Synthetic Procedures for NS560, CQ-X, and CQ-TAMRA

Total: 51 pages

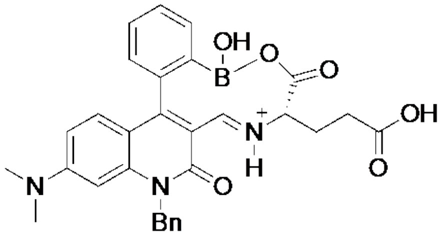

(m/z = 538.22)

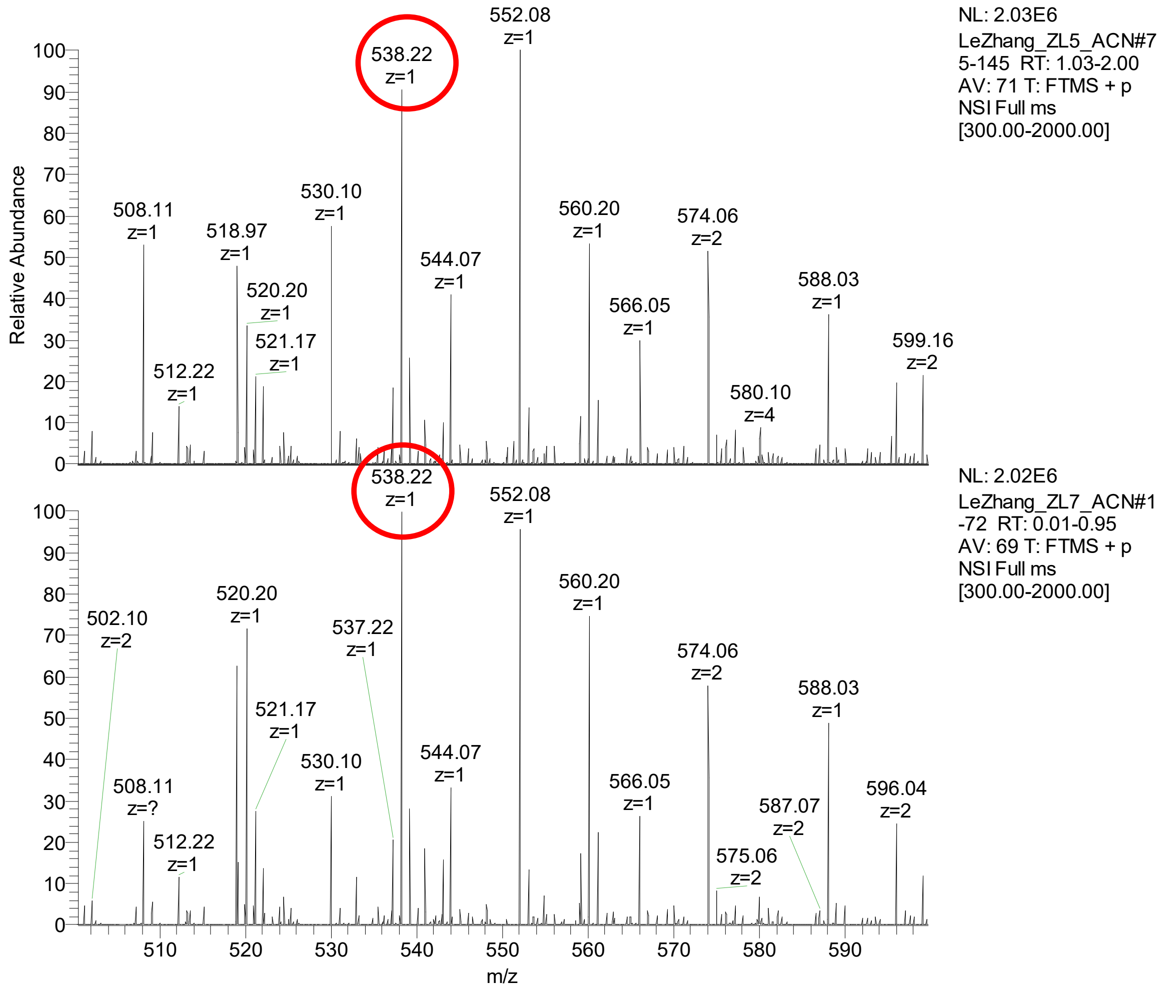

Figure S1. (Upper) mass spectra of NS560 solution upon addition of glutamic acid (0.625 mM NS560, 100 mM glutamic acid, 50%/50% pH =5 buffer/DMSO). (Lower) Mass spectra of NS560 solution upon addition of glutamic acid (0.625 mM NS560, 100 mM glutamic acid, 50%/50% pH = 7.4 buffer/DMSO). Samples were also diluted 1:100 in 70%/1% acetonitrile/FA and loaded into (separate) static nanospray ECONO 12 tips (Proxeon) and analyzed by nano-electrospray ionization in positive-ion on a ThermoScientific LTQ Orbitrap XL mass spectrometer. Typical flow rates from these tips are estimated to be 50nL/min. FTMS data were collected in the Orbitrap

(60,000 resolving power, 300-2000 m/z, 1 microscan, maximum inject time of 50ms, AGC= 5e5) over 1min of infusion. Xcalibur Qual Browser V2.1.0.1139 was used to examine the data.

Spectroscopic Studies

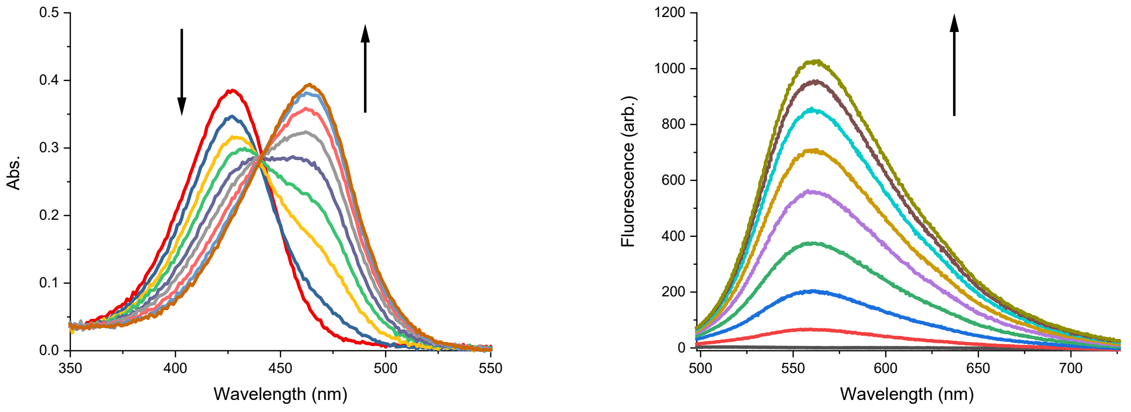

Figure S2. UV/Vis and fluorescence spectroscopy of **NS560** (10 µM in 25 HEPES, 50 mM $Na_2S_2O_3$, 1% DMSO, pH 7.4) with glutamic acid (500 mM in buffer), λ_{ex} = 488 nm, λ_{em} = 560 nm.

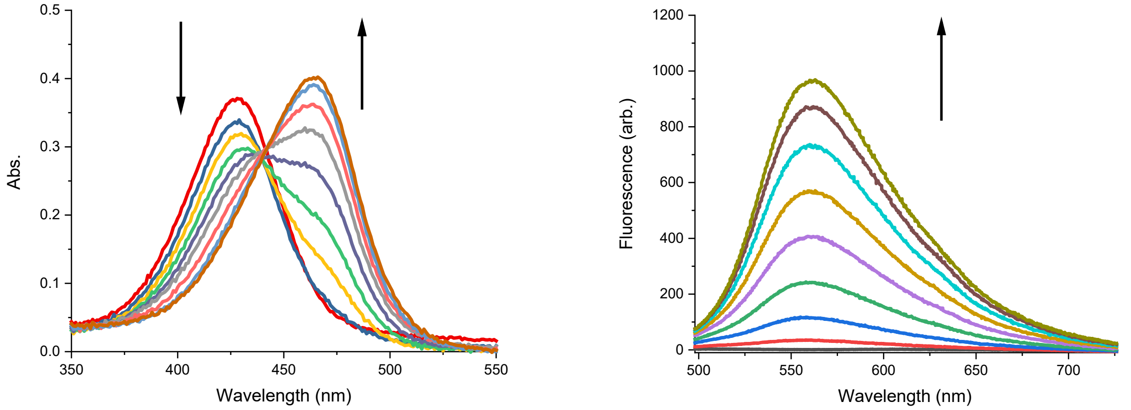

Figure S3. UV/Vis and fluorescence spectroscopy of **NS560** (10 µM in 25 HEPES, 50 mM $Na_2S_2O_3$, 1% DMSO, pH 5.0) with glutamic acid (500 mM in buffer), λ_{ex} = 488 nm, λ_{em} = 560 nm.

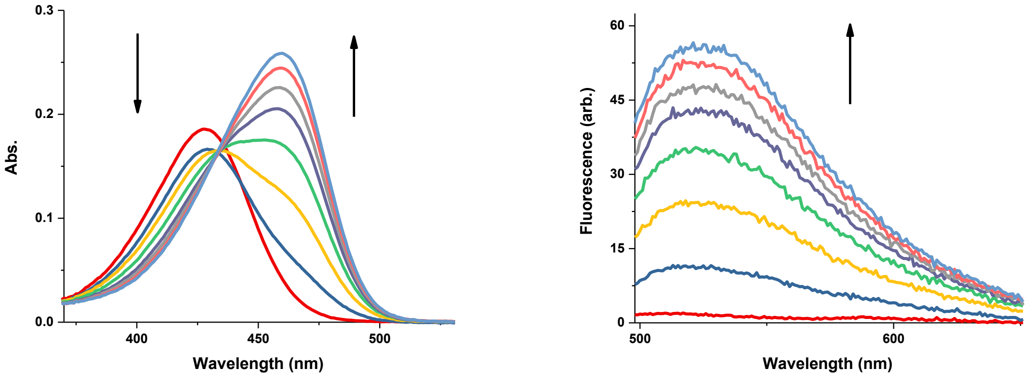

Figure S4. UV/Vis and fluorescence spectroscopy of **NS560** (10 µM in 25 HEPES, 50 mM $Na_2S_2O_3$, 1% DMSO, pH 7.4) with GABA (250 mM in buffer), λ_{ex} = 488 nm, λ_{em} = 560 nm.

.

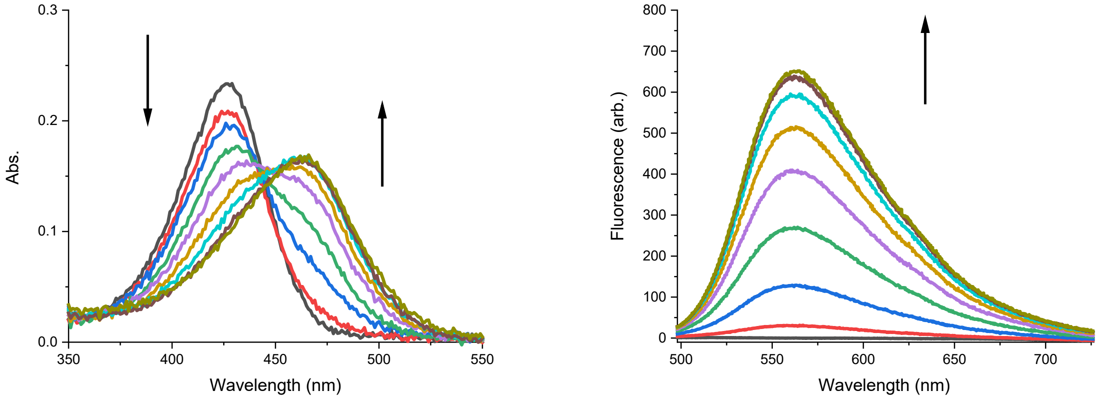

Figure S5. UV/Vis and fluorescence spectroscopy of **NS560** (10 µM in 25 HEPES, 50 mM $Na_2S_2O_3$, 1% DMSO, pH 7.4) with alanine (250 mm in buffer), λ_{ex} = 488 nm, λ_{em} = 560 nm.

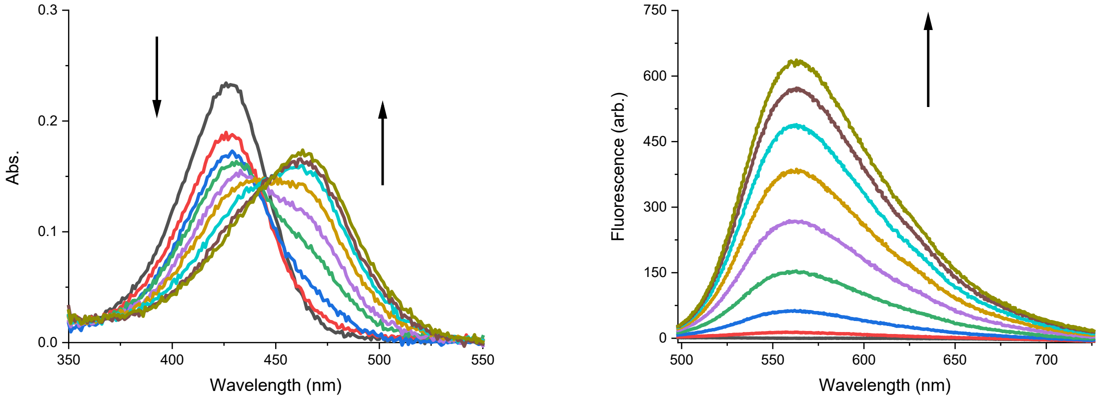

Figure S6. UV/Vis and fluorescence spectroscopy of **NS560** (10 µM in 25 HEPES, 50 mM $Na_2S_2O_3$, 1% DMSO, pH 5.0) with alanine (250 mM in buffer), λ_{ex} = 488 nm, λ_{em} = 560 nm.

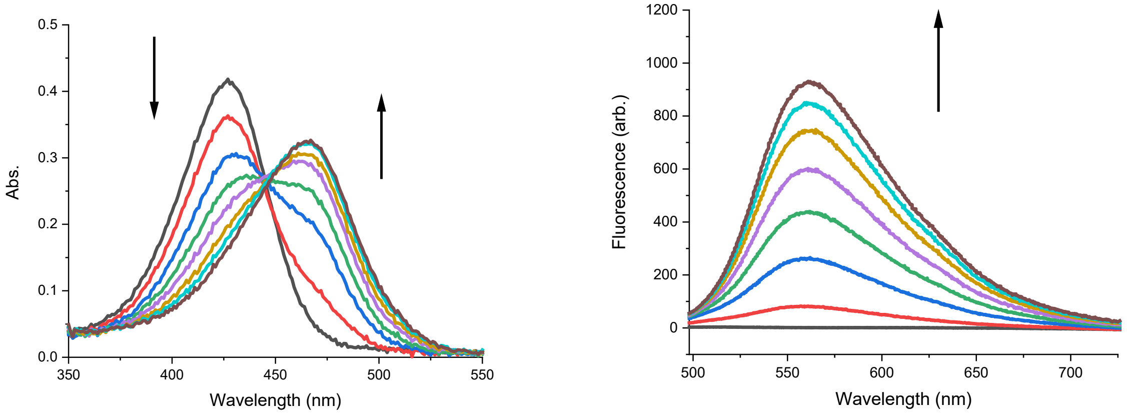

Figure S7. UV/Vis and fluorescence spectroscopy of **NS560** (10 μM in 25 HEPES, 50 mM $Na_2S_2O_3$, 1% DMSO, pH 7.4) with arginine (500 mM in buffer), λ_{ex} = 488 nm, λ_{em} = 560 nm.

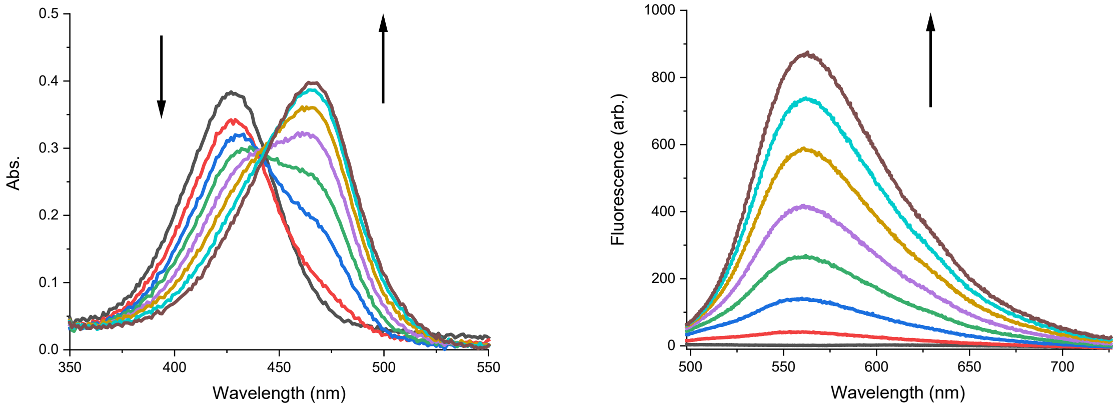

Figure S8. UV/Vis and fluorescence spectroscopy of **NS560** (10 μM in 25 HEPES, 50 mM $Na_2S_2O_3$, 1% DMSO, pH 5.0) with arginine (500 mM in buffer), λ_{ex} = 488 nm, λ_{em} = 560 nm.

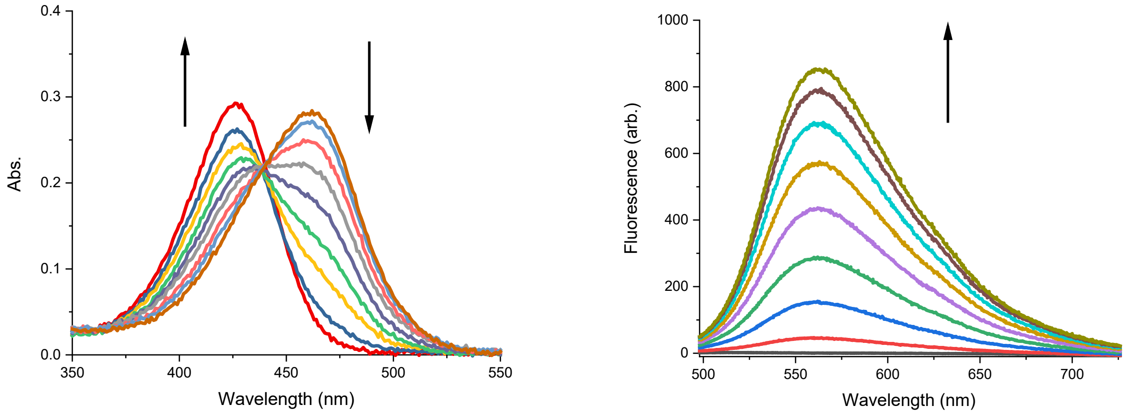

Figure S9. UV/Vis and fluorescence spectroscopy of **NS560** (10 µM in 25 HEPES, 50 mM $Na_2S_2O_3$, 1% DMSO, pH 7.4) with aspartic acid (500 mM in buffer), λ_{ex} = 488 nm, λ_{em} = 560 nm.

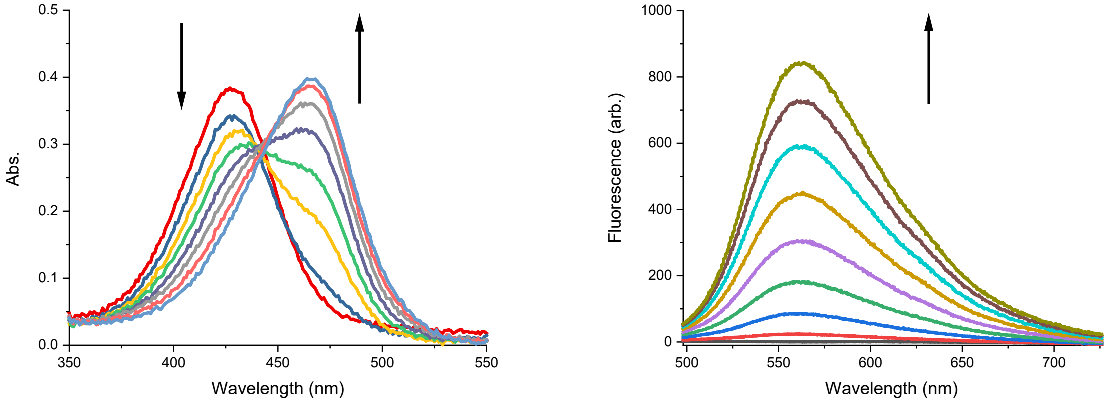

Figure S10. UV/Vis and fluorescence spectroscopy of **NS560** (10 µM in 25 HEPES, 50 mM $Na_2S_2O_3$, 1% DMSO, pH 5.0) with aspartic acid (500 mM in buffer), λ_{ex} = 488 nm, λ_{em} = 560 nm.

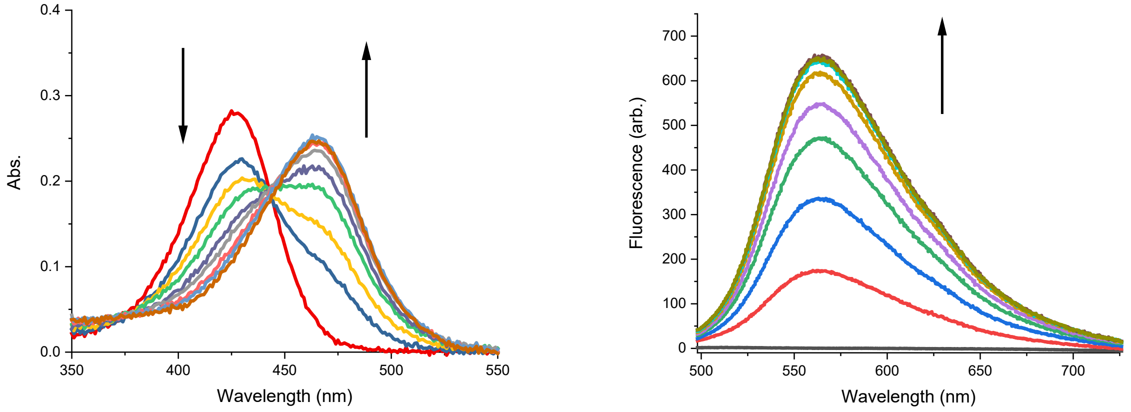

Figure S11. UV/Vis and fluorescence spectroscopy of **NS560** (10 µM in 25 HEPES, 50 mM $Na_2S_2O_3$, 1% DMSO, pH 7.4) with asparagine (250 mM in buffer), λ_{ex} = 488 nm, λ_{em} = 560 nm.

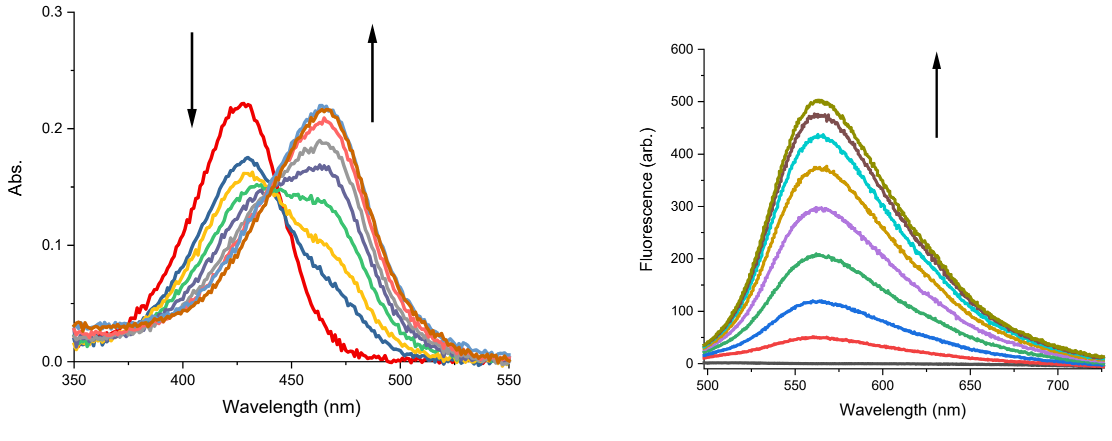

Figure S12. UV/Vis and fluorescence spectroscopy of **NS560** (10 µM in 25 HEPES, 50 mM $Na_2S_2O_3$, 1% DMSO, pH 5.0) with asparagine (250 mM in buffer), λ_{ex} = 488 nm, λ_{em} = 560 nm.

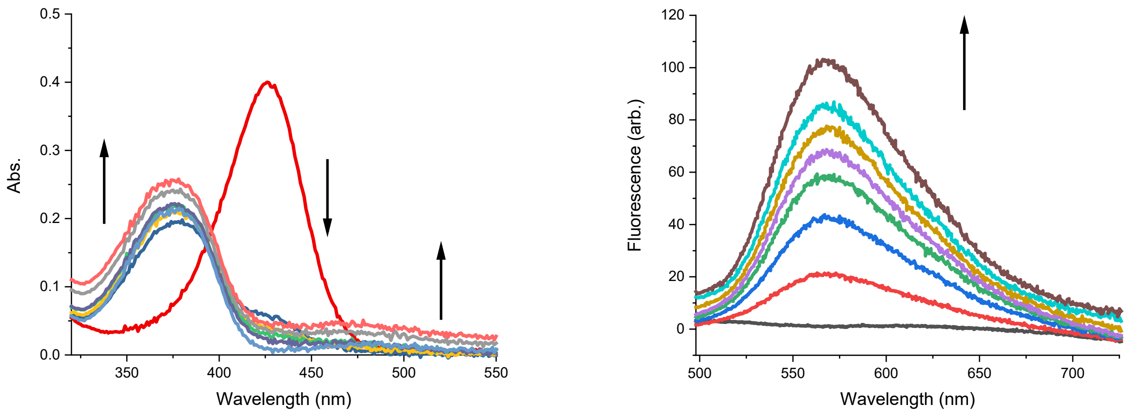

Figure S13. UV/Vis and fluorescence spectroscopy of **NS560** (10 µM in 25 HEPES, 50 mM $Na_2S_2O_3$, 1% DMSO, pH 7.4) with cysteine (100 mM in buffer), λ_{ex} = 488 nm, λ_{em} = 560 nm.

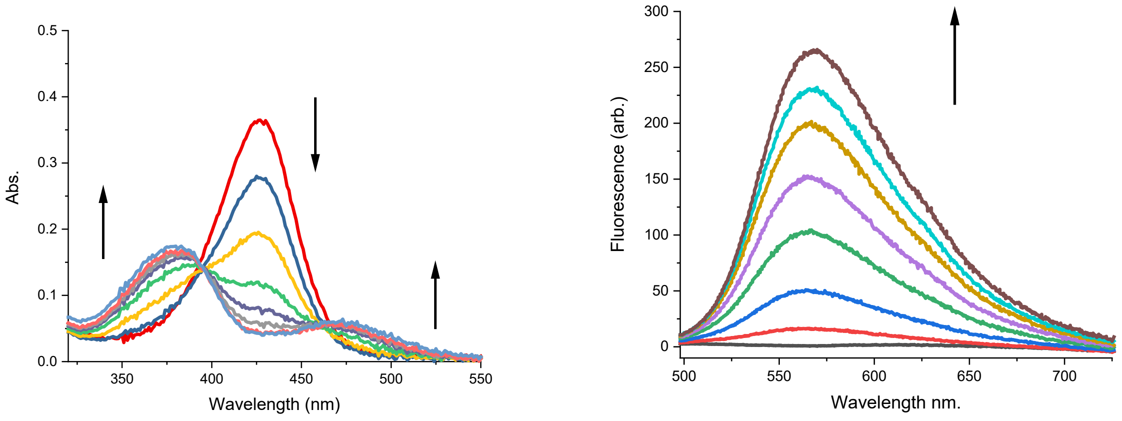

Figure S14. UV/Vis and fluorescence spectroscopy of **NS560** (10 µM in 25 HEPES, 50 mM $Na_2S_2O_3$, 1% DMSO, pH 5.0) with cysteine (100 mM in buffer), λ_{ex} = 488 nm, λ_{em} = 560 nm.

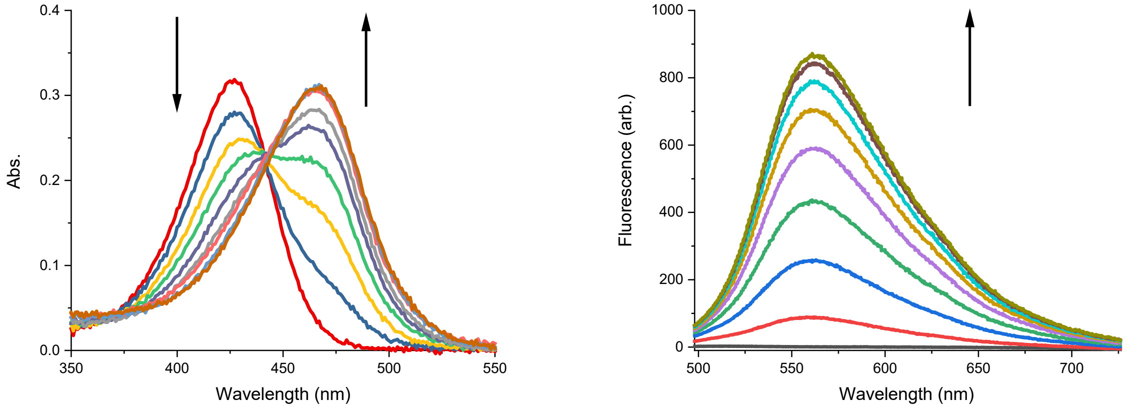

Figure S15. UV/Vis and fluorescence spectroscopy of **NS560** (10 µM in 25 HEPES, 50 mM $Na_2S_2O_3$, 1% DMSO, pH 7.4) with glutamine (250 mM in buffer), λ_{ex} = 488 nm, λ_{em} = 560 nm.

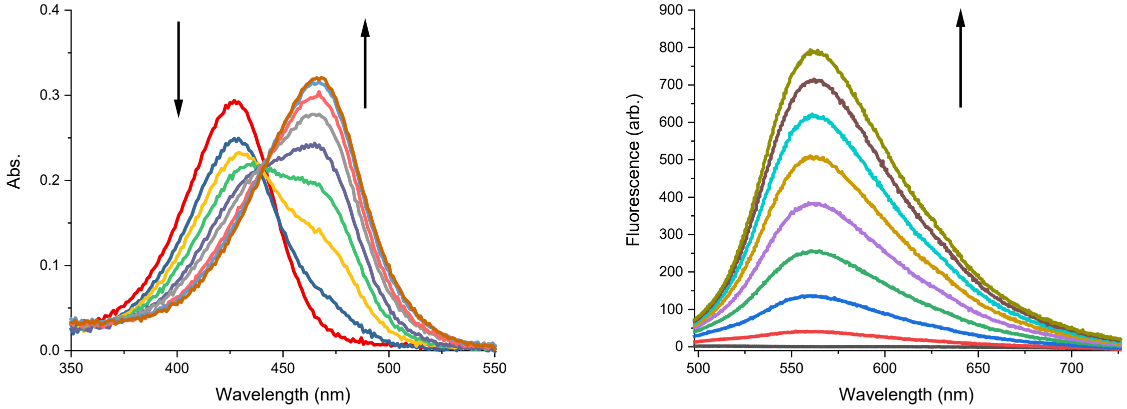

Figure S16. UV/Vis and fluorescence spectroscopy of **NS560** (10 µM in 25 HEPES, 50 mM $Na_2S_2O_3$, 1% DMSO, pH 5.0) with glutamine (250 mM in buffer), λ_{ex} = 488 nm, λ_{em} = 560 nm.

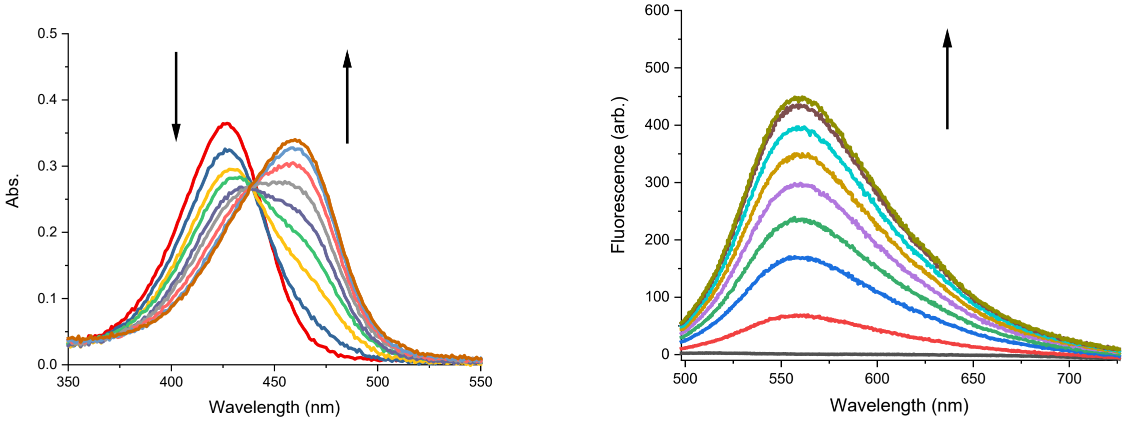

Figure S17. UV/Vis and fluorescence spectroscopy of **NS560** (10 µM in 25 HEPES, 50 mM $Na_2S_2O_3$, 1% DMSO, pH 7.4) with glycine (500 mM in buffer), λ_{ex} = 488 nm, λ_{em} = 560 nm.

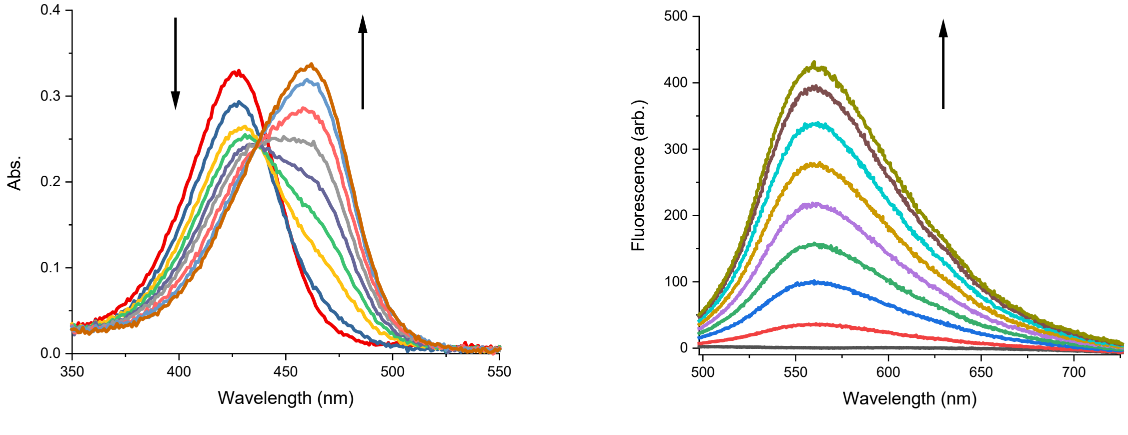

Figure S18. UV/Vis and fluorescence spectroscopy of **NS560** (10 µM in 25 HEPES, 50 mM $Na_2S_2O_3$, 1% DMSO, pH 5.0) with glycine (500 mM in buffer), λ_{ex} = 488 nm, λ_{em} = 560 nm.

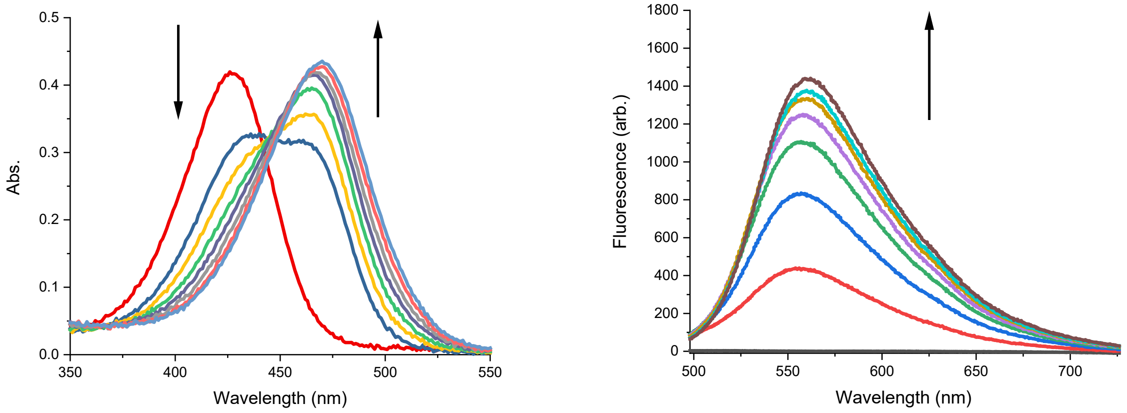

Figure S19. UV/Vis and fluorescence spectroscopy of **NS560** (10 µM in 25 HEPES, 50 mM $Na_2S_2O_3$, 1% DMSO, pH 7.4) with histidine (500 mM in buffer), λ_{ex} = 488 nm, λ_{em} = 560 nm.

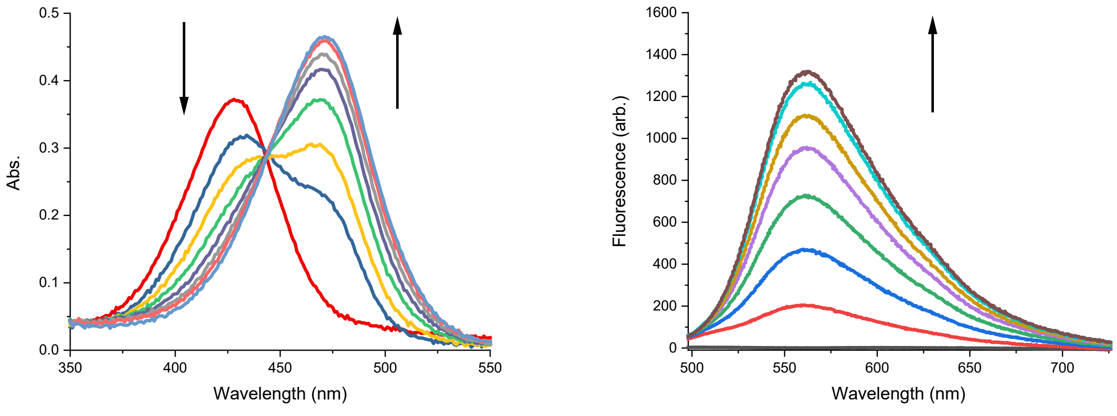

Figure S20. UV/Vis and fluorescence spectroscopy of **NS560** (10 µM in 25 HEPES, 50 mM $Na_2S_2O_3$, 1% DMSO, pH 5.0) with histidine (500 mM in buffer), λ_{ex} = 488 nm, λ_{em} = 560 nm.

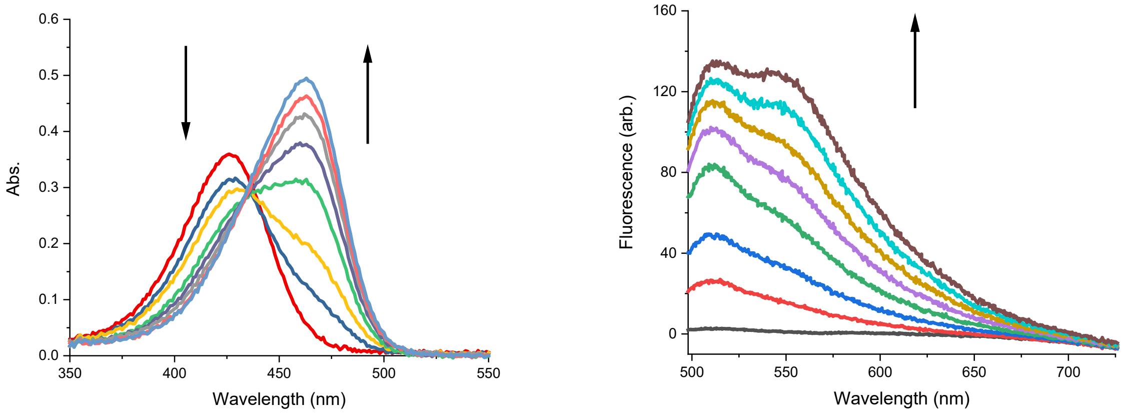

Figure S21. UV/Vis and fluorescence spectroscopy of **NS560** (10 µM in 25 HEPES, 50 mM $Na_2S_2O_3$, 1% DMSO, pH 7.4) with isoleucine(100 mM in buffer), λ_{ex} = 488 nm, λ_{em} = 560 nm.

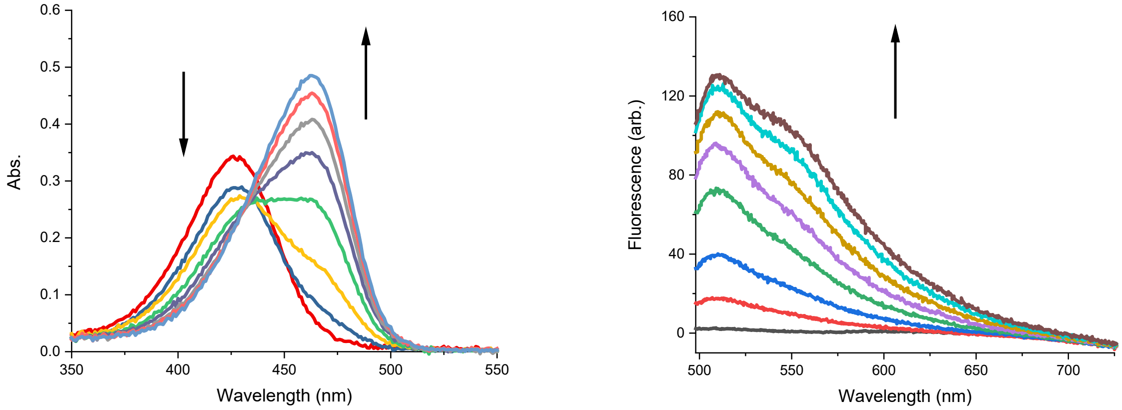

Figure S22. UV/Vis and fluorescence spectroscopy of **NS560** (10 µM in 25 HEPES, 50 mM $Na_2S_2O_3$, 1% DMSO, pH 5.0) with isoleucine (100 mM in buffer), λ_{ex} = 488 nm, λ_{em} = 560 nm.

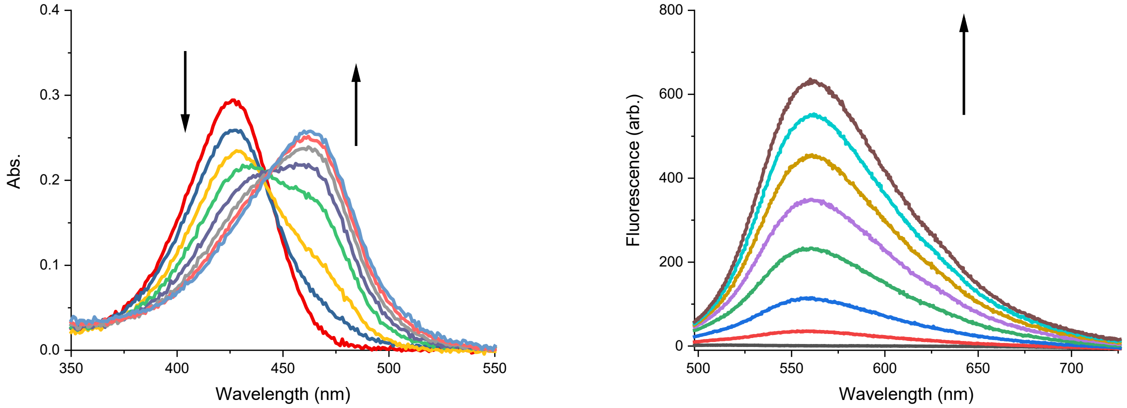

Figure S23. UV/Vis and fluorescence spectroscopy of **NS560** (10 µM in 25 HEPES, 50 mM $Na_2S_2O_3$, 1% DMSO, pH 7.4) with leucine (100 mM in buffer), λ_{ex} = 488 nm, λ_{em} = 560 nm.

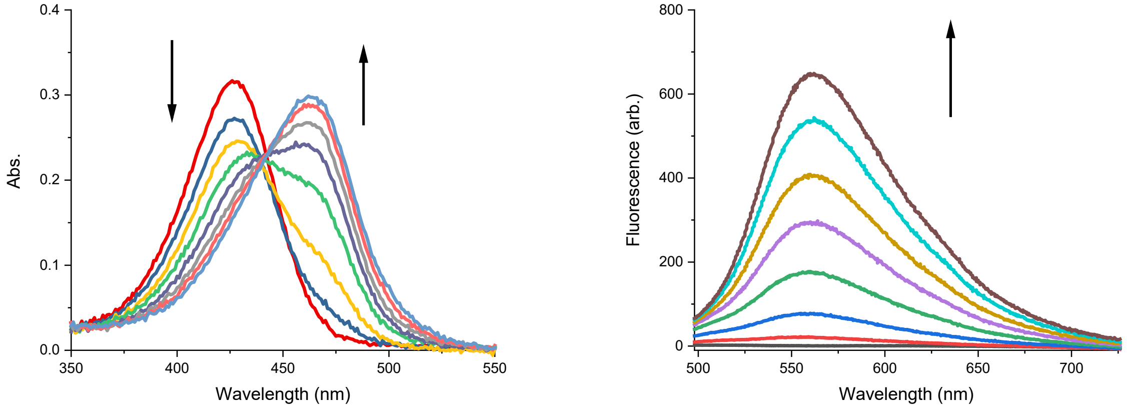

Figure S24. UV/Vis and fluorescence spectroscopy of **NS560** (10 µM in 25 HEPES, 50 mM $Na_2S_2O_3$, 1% DMSO, pH 5.0) with leucine (100 mM in buffer), λ_{ex} = 488 nm, λ_{em} = 560 nm.

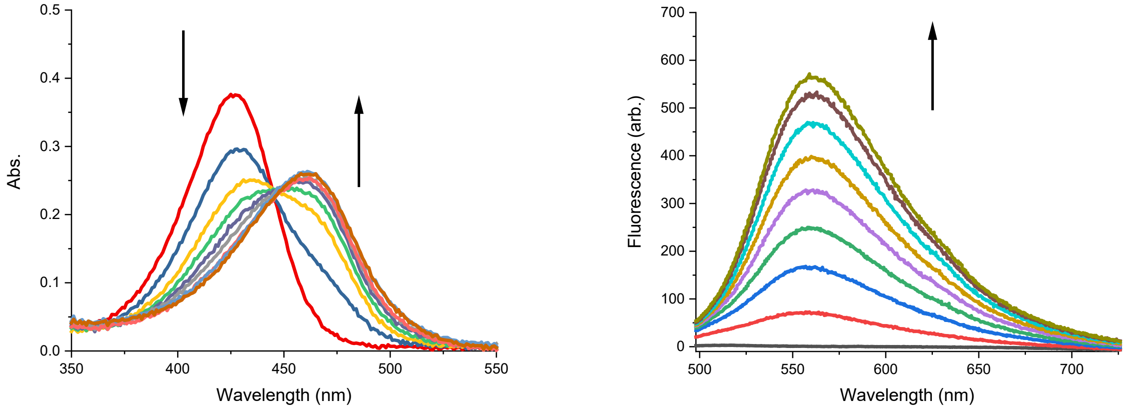

Figure S25. UV/Vis and fluorescence spectroscopy of **NS560** (10 µM in 25 HEPES, 50 mM $Na_2S_2O_3$, 1% DMSO, pH 7.4) with lysine (500 mM in buffer), λ_{ex} = 488 nm, λ_{em} = 560 nm.

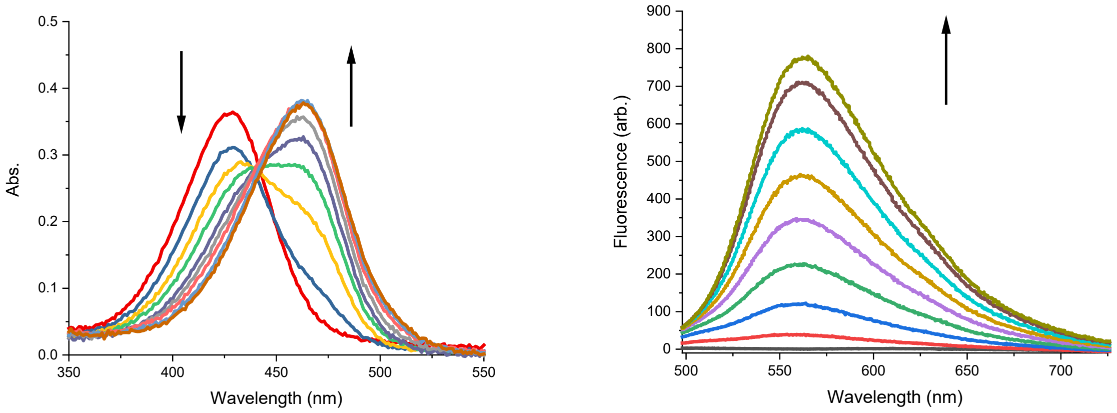

Figure S26. UV/Vis and fluorescence spectroscopy of **NS560** (10 µM in 25 HEPES, 50 mM $Na_2S_2O_3$, 1% DMSO, pH 5.0) with lysine (500 mM in buffer), λ_{ex} = 488 nm, λ_{em} = 560 nm.

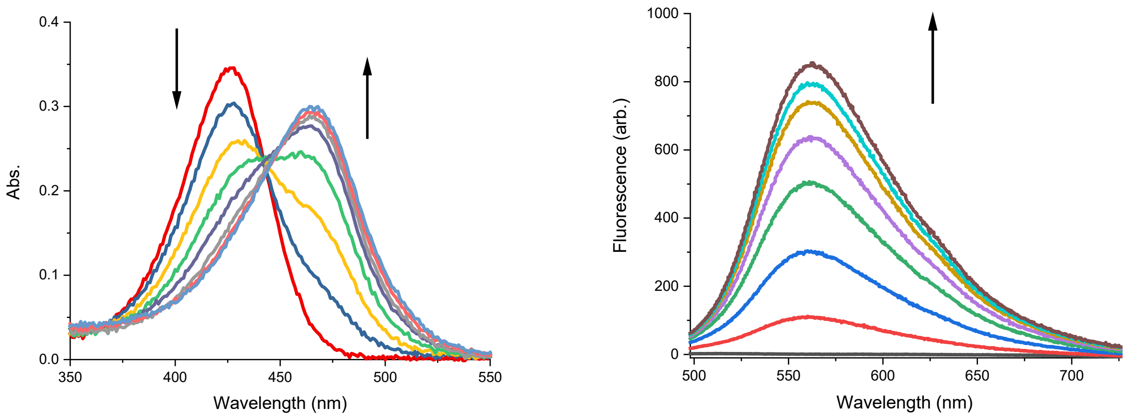

Figure S27. UV/Vis and fluorescence spectroscopy of **NS560** (10 µM in 25 HEPES, 50 mM $Na_2S_2O_3$, 1% DMSO, pH 7.4) with methionine (100 mM in buffer), λ_{ex} = 488 nm, λ_{em} = 560 nm.

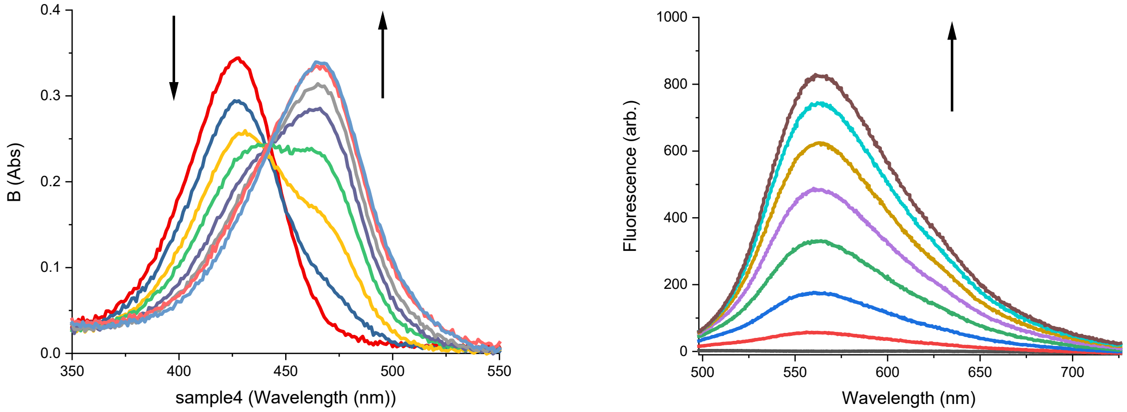

Figure S28. UV/Vis and fluorescence spectroscopy of **NS560** (10 µM in 25 HEPES, 50 mM $Na_2S_2O_3$, 1% DMSO, pH 5.0) with methionine (100 mM in buffer), λ_{ex} = 488 nm, λ_{em} = 560 nm.

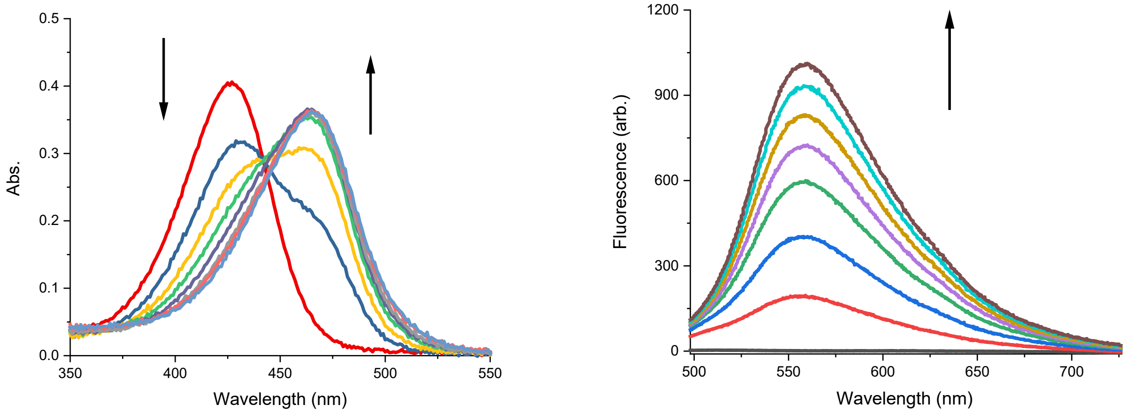

Figure S29. UV/Vis and fluorescence spectroscopy of **NS560** (10 µM in 25 HEPES, 50 mM $Na_2S_2O_3$, 1% DMSO, pH 7.4) with phenylalanine (100 mM in buffer), λ_{ex} = 488 nm, λ_{em} = 560 nm.

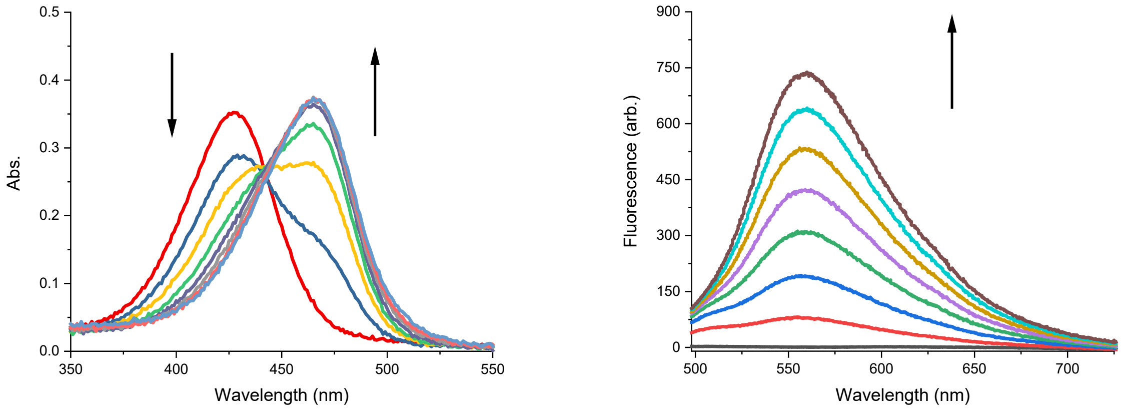

Figure S30. UV/Vis and fluorescence spectroscopy of **NS560** (10 µM in 25 HEPES, 50 mM $Na_2S_2O_3$, 1% DMSO, pH 5.0) with phenylalanine (100 mM in buffer), λ_{ex} = 488 nm, λ_{em} = 560 nm.

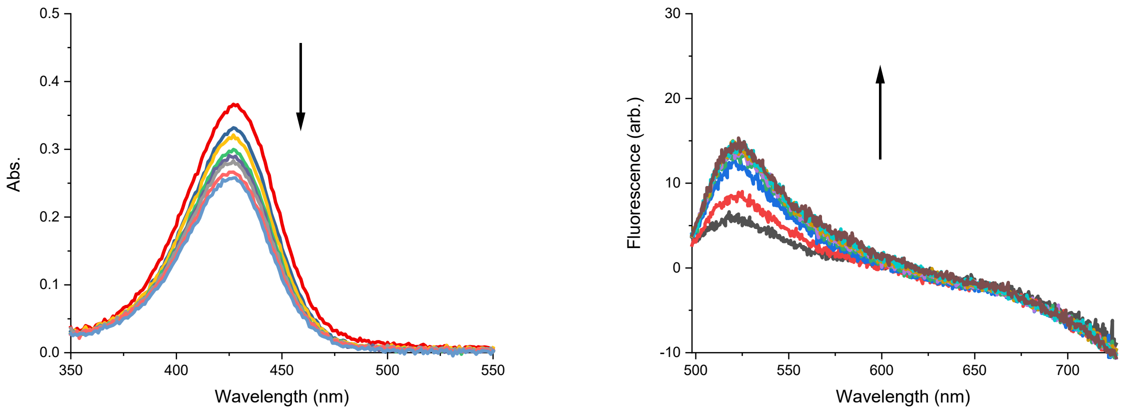

Figure S31. UV/Vis and fluorescence spectroscopy of **NS560** ((10 µM in 25 HEPES, 50 mM $Na_2S_2O_3$, 1% DMSO, pH 7.4) with proline (1 M in buffer), λ_{ex} = 488 nm, λ_{em} = 560 nm.

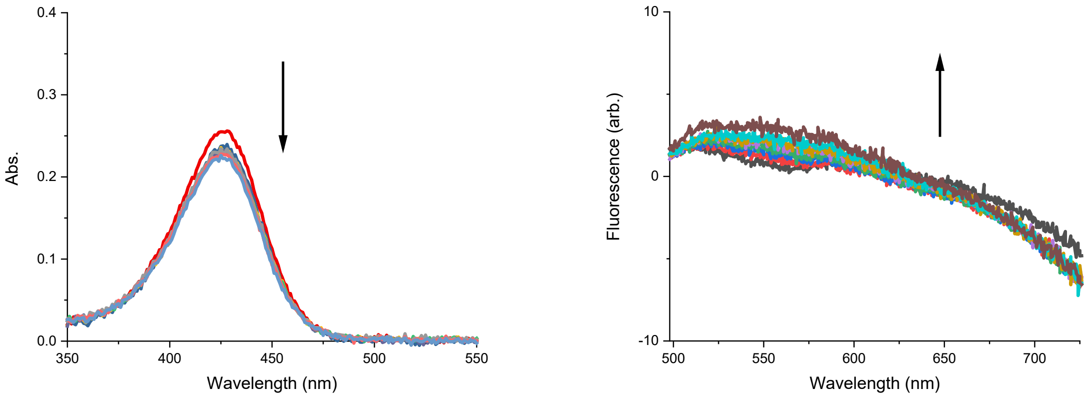

Figure S32. UV/Vis and fluorescence spectroscopy of **NS560** (10 µM in 25 HEPES, 50 mM $Na_2S_2O_3$, 1% DMSO, pH 5.0) with proline (1 M in buffer), λ_{ex} = 488 nm, λ_{em} = 560 nm.

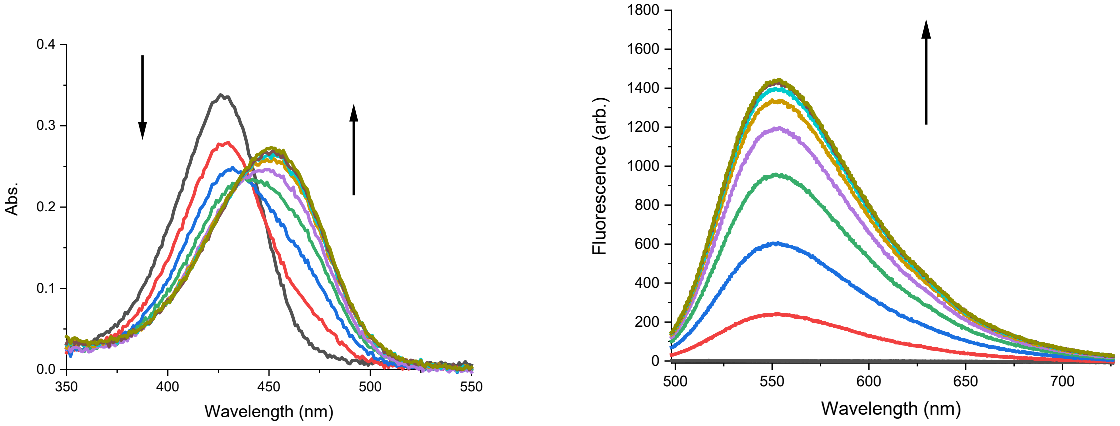

Figure S33. UV/Vis and fluorescence spectroscopy of **NS560** ((10 µM in 25 HEPES, 50 mM $Na_2S_2O_3$, 1% DMSO, pH 7.4) with serine (500 mm in buffer), λ_{ex} = 488 nm, λ_{em} = 560 nm.

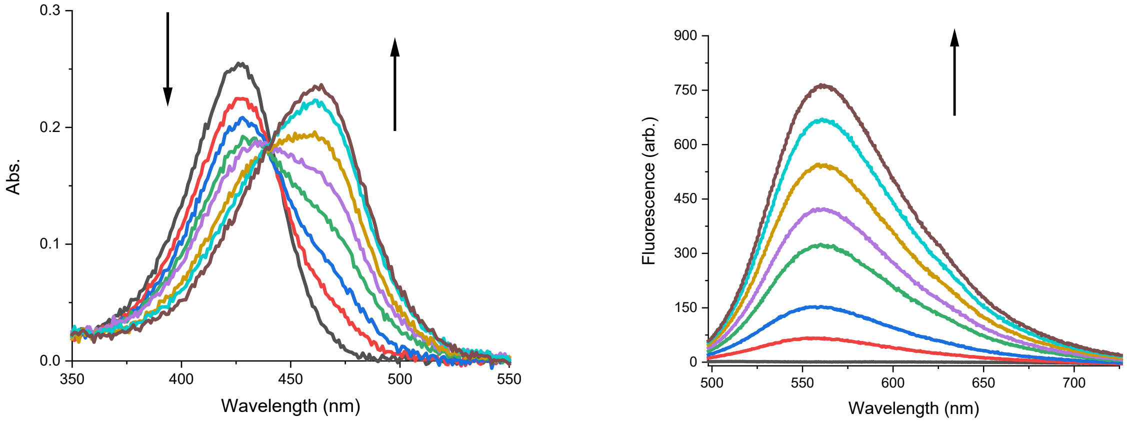

Figure S34. UV/Vis and fluorescence spectroscopy of **NS560** (10 µM in 25 HEPES, 50 mM $Na_2S_2O_3$, 1% DMSO, pH 5.0) with serine (500 mM in buffer), λ_{ex} = 488 nm, λ_{em} = 560 nm.

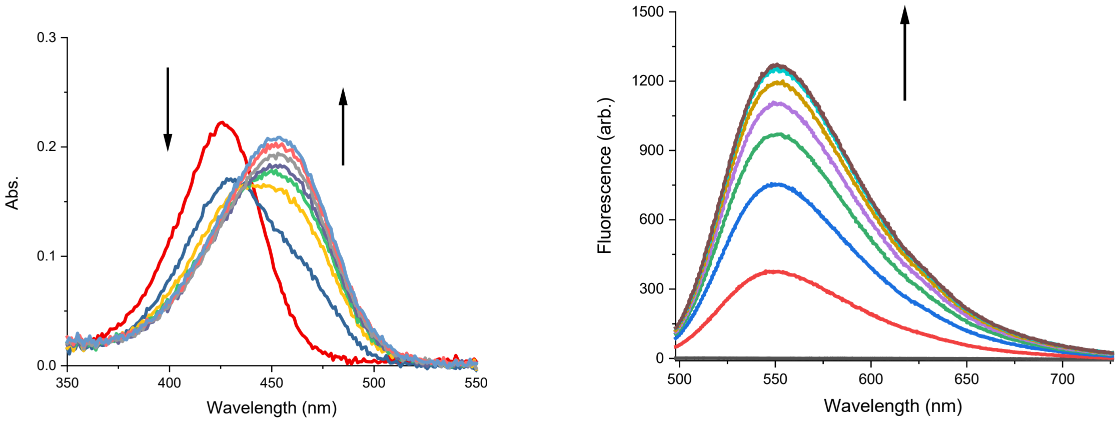

Figure S35. UV/Vis and fluorescence spectroscopy of **NS560** (10 µM in 25 HEPES, 50 mM $Na_2S_2O_3$, 1% DMSO, pH 7.4) with threonine (250 mM in buffer), λ_{ex} = 488 nm, λ_{em} = 560 nm.

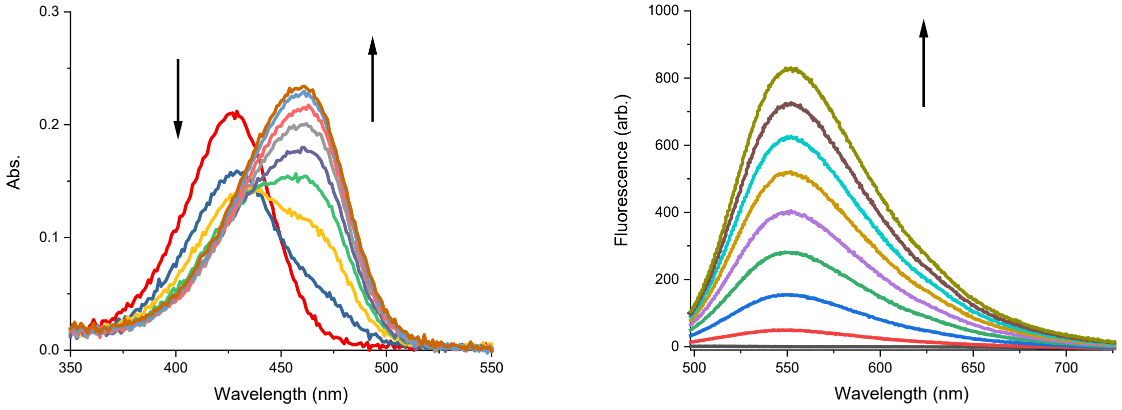

Figure S36. UV/Vis and fluorescence spectroscopy of **NS560** (10 µM in 25 HEPES, 50 mM $Na_2S_2O_3$, 1% DMSO, pH 5.0) with threonine (250 mM in buffer), λ_{ex} = 488 nm, λ_{em} = 560 nm.

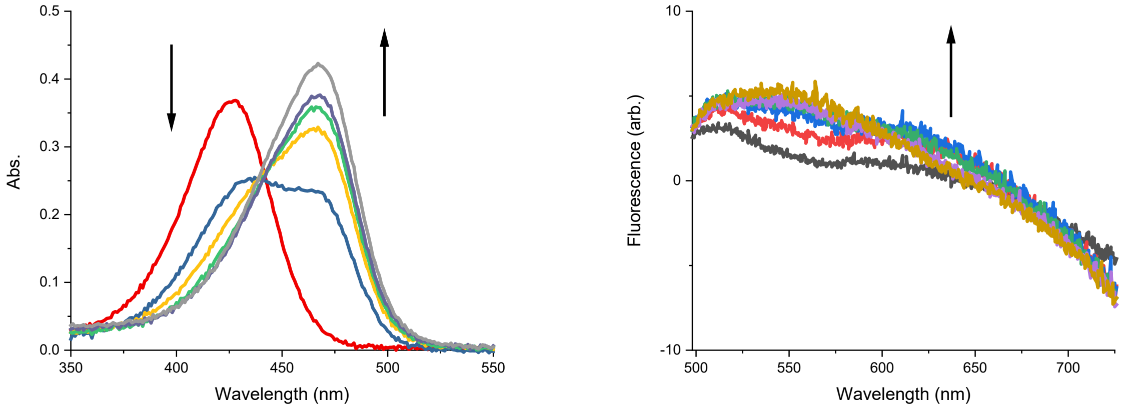

Figure S37. UV/Vis and fluorescence spectroscopy of **NS560** (10 µM in 25 HEPES, 50 mM $Na_2S_2O_3$, 1% DMSO, pH 7.4) with tryptophan (100 mM in buffer), λ_{ex} = 488 nm, λ_{em} = 560 nm.

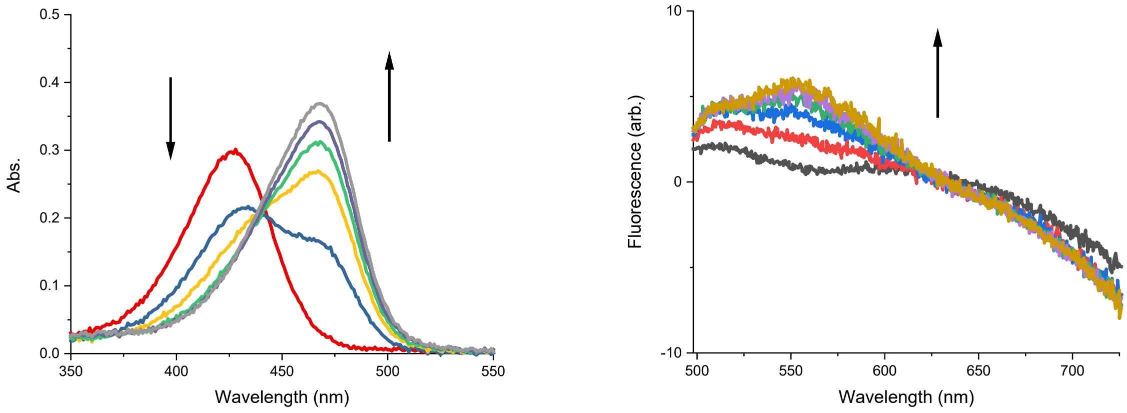

Figure S38. UV/Vis and fluorescence spectroscopy of **NS560** (10 µM in 25 HEPES, 50 mM $Na_2S_2O_3$, 1% DMSO, pH 5.0) with tryptophan (100 mM in buffer), λ_{ex} = 488 nm, λ_{em} = 560 nm.

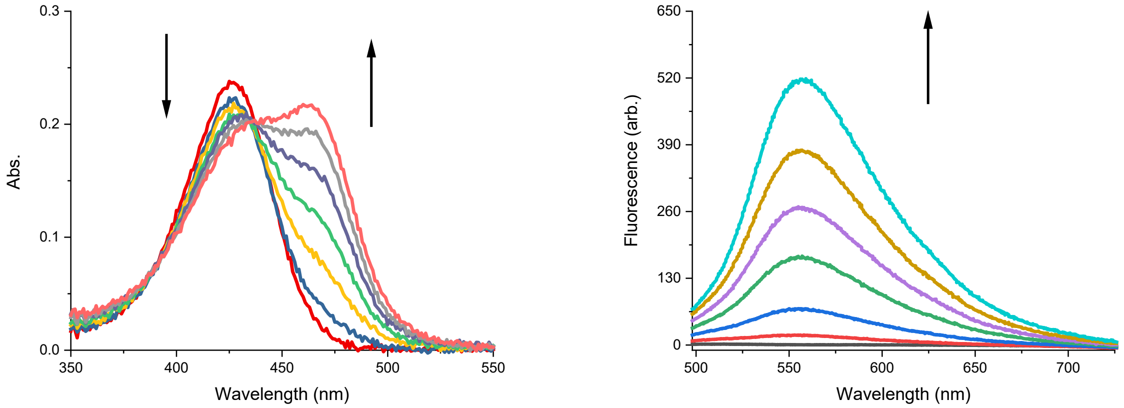

Figure S39. UV/Vis and fluorescence spectroscopy of **NS560** (10 µM in 25 HEPES, 50 mM $Na_2S_2O_3$, 1% DMSO, pH 7.4) with tyrosine in (10 mM in 80%/20% buffer/DMSO) , λ_{ex} = 488 nm, λ_{em} = 560 nm.

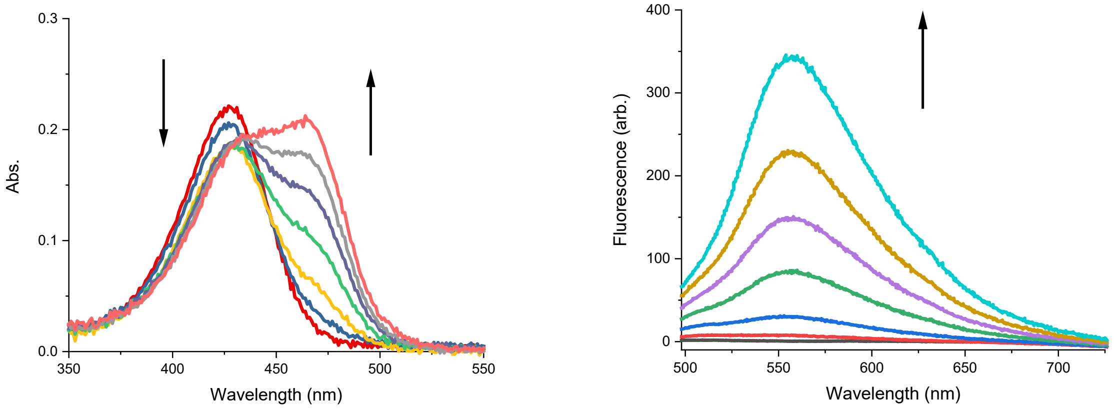

Figure S40. UV/Vis and fluorescence spectroscopy of **NS560** (10 µM in 25 HEPES, 50 mM $Na_2S_2O_3$, 1% DMSO, pH 5.0) with tyrosine (10 mM in 80%/20% buffer/DMSO), λ_{ex} = 488 nm, λ_{em} = 560 nm.

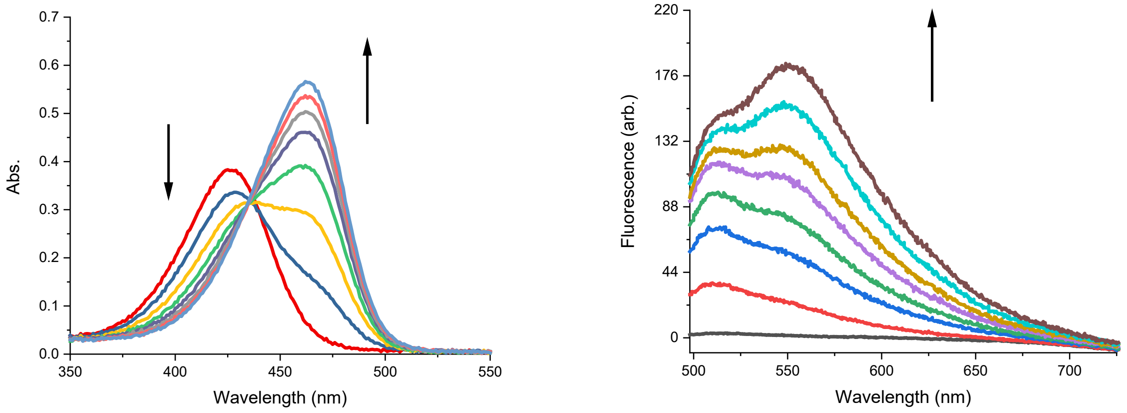

Figure S41. UV/Vis and fluorescence spectroscopy of **NS560** (10 µM in 25 HEPES, 50 mM $Na_2S_2O_3$, 1% DMSO, pH 7.4) with valine (250 mM in buffer), λ_{ex} = 488 nm, λ_{em} = 560 nm.

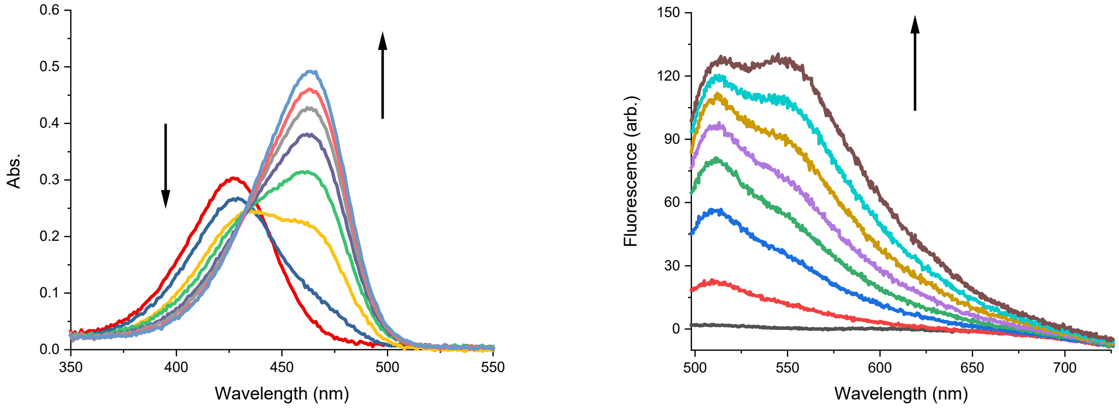

Figure S42. UV/Vis and fluorescence spectroscopy of **NS560** (10 µM in 25 HEPES, 50 mM $Na_2S_2O_3$, 1% DMSO, pH 5.0) with valine (250 mM in buffer), λ_{ex} = 488 nm, λ_{em} = 560 nm.

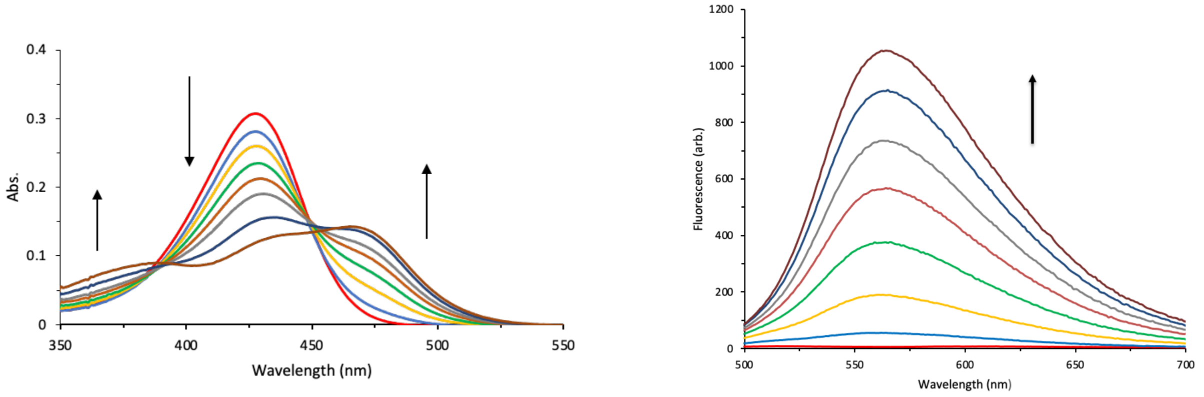

Figure S43. UV/Vis and fluorescence spectroscopy of **NS560** (10 µM in 25 HEPES, 50 mM $Na_2S_2O_3$, 1% DMSO, pH 7.4) with glutathione (250 mM in buffer), λ_{ex} = 488 nm, λ_{em} = 560 nm.

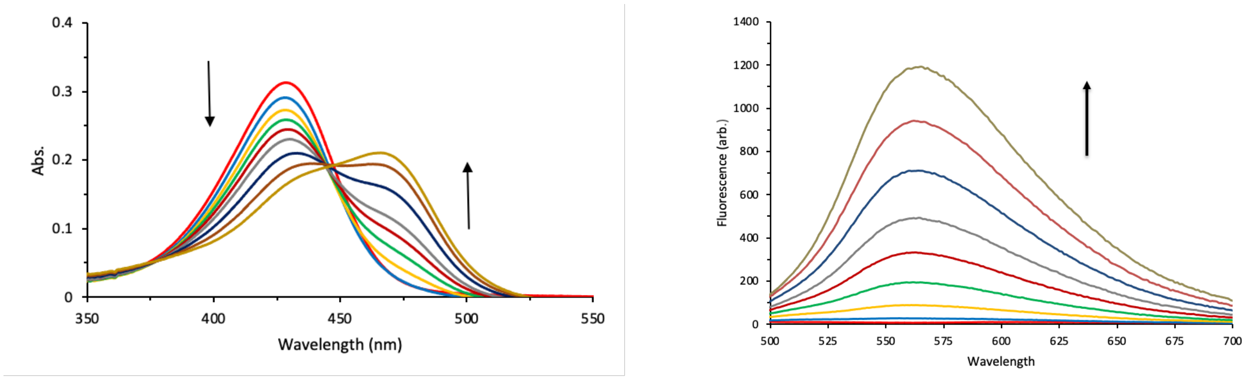

Figure S44. UV/Vis and fluorescence spectroscopy of **NS560** (10 µM in 25 HEPES, 50 mM $Na_2S_2O_3$, 1% DMSO, pH 5.0) with glutathione (250 mM in buffer), λ_{ex} = 488 nm, λ_{em} = 560 nm.

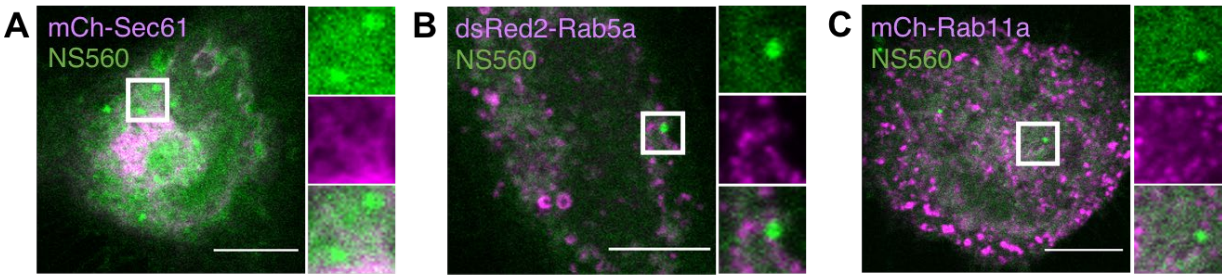

Figure S45. NS560 labeling surrounds ER but does not colocalize with early or recycling endosomes. HeLa cells in basal conditions overexpressing *(A)* mCherry-Sec61 (ER marker), *(B)* dsRed2-Rab5a, or *(C)* mCherry-Rab11a were labeled with NS560 for 45 minutes and representative images from confocal microscopy are shown. White boxes highlight regions with occasional NS560 foci. Scale bar, 10 µm. Representative images of two independent biological replicates.

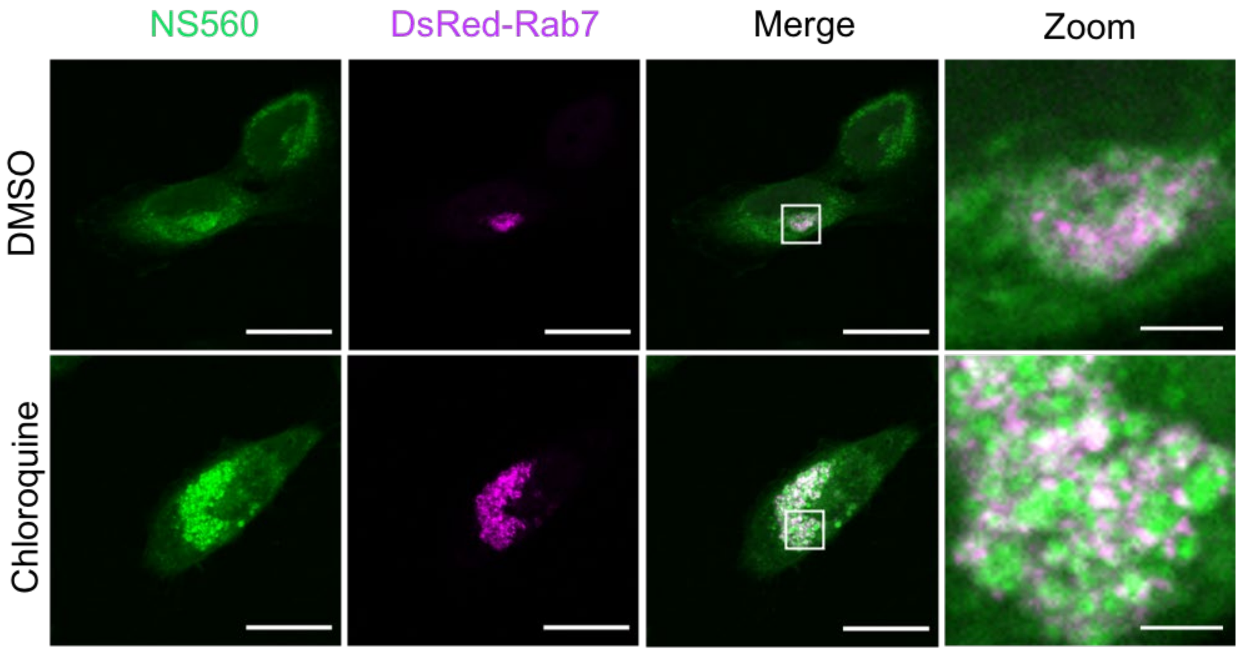

Figure S46. NS560 localizes to late endosomes. HeLa cells overexpressing dsRed-Rab7 were treated with 25 µM CQ for 24 hours and labeled with NS560 for the final 45 minutes. Representative images of two independent biological replicates. Scale bar, 10 µm or 2 µm for zoom panel.

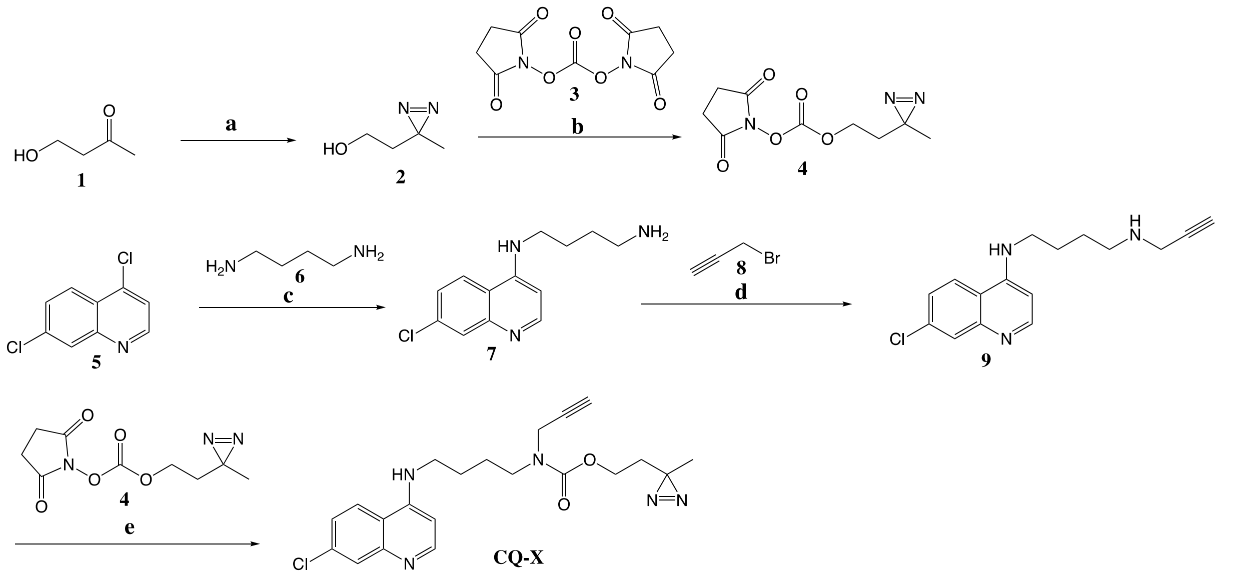

Figure S47. CQ-X design and synthesis.

Reagents and conditions. (a) NH_3(l)/NH_2OSO_3H/I_2/TEA/MeOH, -78˚C, Overnight, 33%. (b) **3**, MeCN/TEA/RT, Overnight, 85%. (c) **6**, 110˚C, 6 hrs, 45%. (d) **8**, K_2CO_3, EtOH, RT, Overnight, 52% (e) **4**, DCM, RT, 10 hrs, 66%.

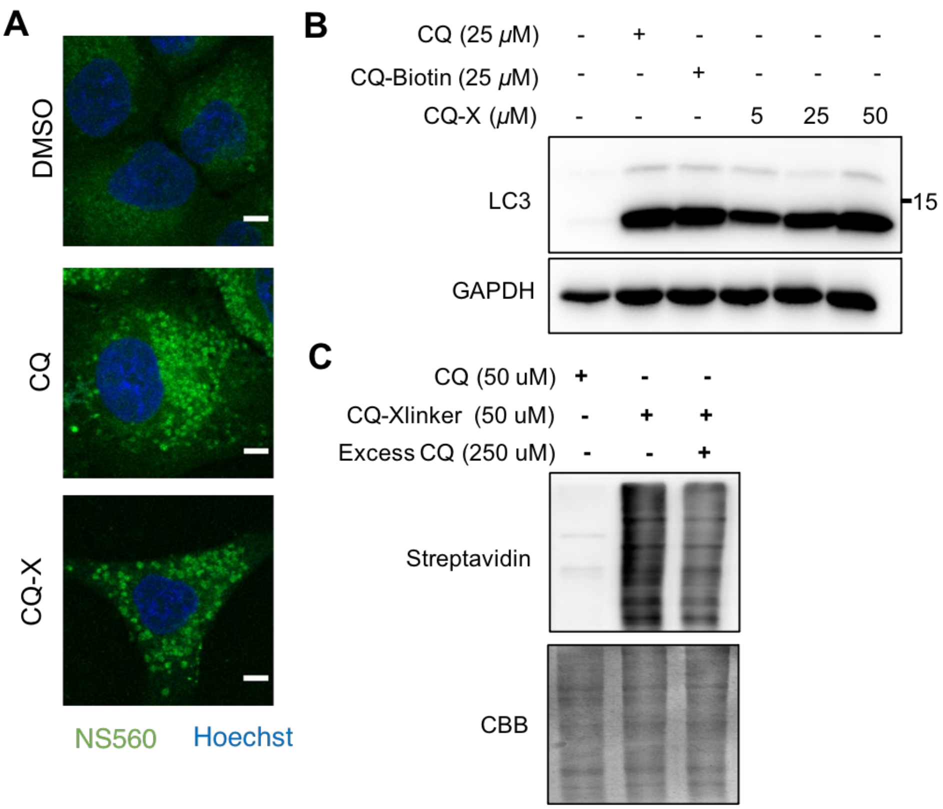

Figure S48. CQ derivatives maintain a similar phenotype upon microscopic analysis compared to the parent compound. (A) A549 cells were treated with 25 µM of chloroquine or CQ-X for 18 hours and labeled with NS560. Representative images. Scale bar, 5 µm. (B) Cells were treated the same as (A) but lysates were collected for Western blot to detect LC3 and GAPDH. (C) Western blot to verify biotinylation of CQ-X crosslinked proteins after UV and click chemistry. Representative Western blot of samples used in proteomics.

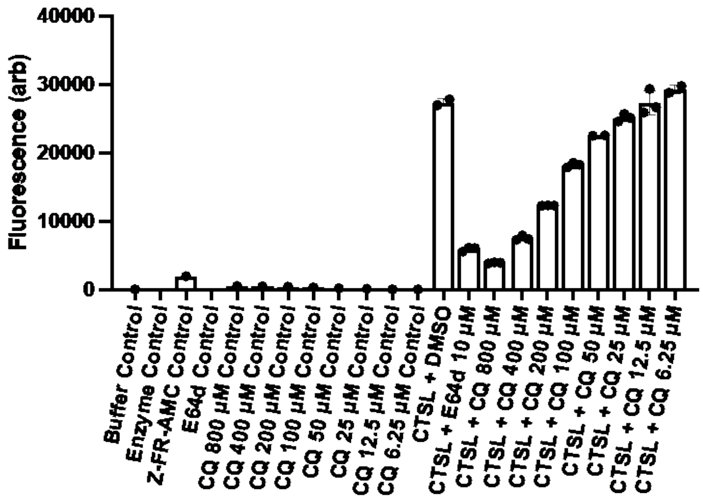

Figure S49. CTSL activity assay F_{445} values. Fluorescent values at 445 nm, including enzyme and substrate controls, are depicted after excitation at 340 nm. Each CTSL and substrate reaction was performed as technical replicates for 30 minutes at room temperature. % activity was calculated from these values, and the IC_{50} curve is plotted in Fig 6A.

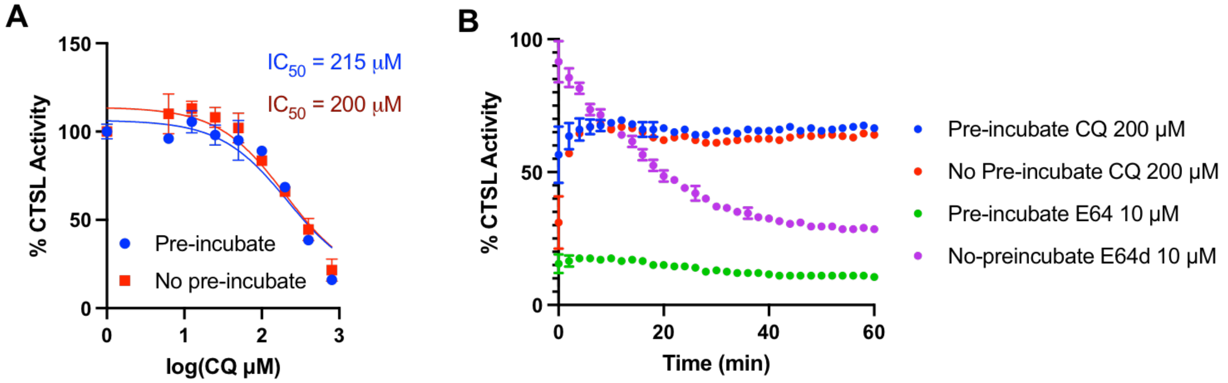

Figure S50. Chloroquine is not a covalent inhibitor of CTSL. CTSL hydrolysis of dipeptide substrate Z-FR-AMC was monitored via fluorescence of AMC at 445 nm. Reaction performed at pH 5.5 in 100 mM MES-NaOH, 150 mM NaCl, and 7.5 mM DTT for 60 minutes with fluorescent measurements taken every 2 minutes. (A) Dose-dependent enzyme inhibition and relative IC_{50} curves 10 minutes after substrate addition are depicted with or without 30-minute chloroquine pre-incubation. (B) Comparison of CQ and E64d inhibition of CTSL over with or without different pre-incubation time. CQ inhibition is independent of preincubation time, while E64d inhibition is dependent on preincubation time, suggesting that CQ is not a covalent inhibitor.

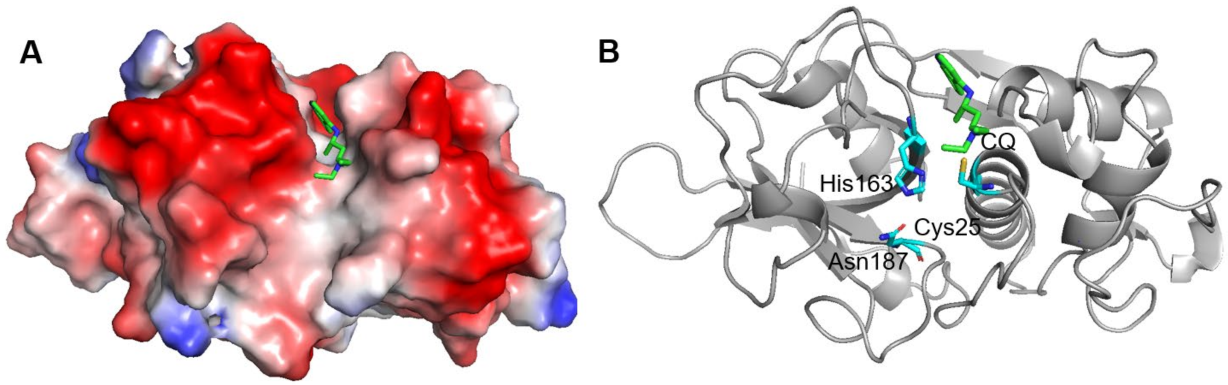

Figure S51. Computational docking study of chloroquine in the active site of CTSL. The CQ molecule is docked into the CSTL using the reference structure found in the PDB database (PDB ID: 2XU3) using MOE. (A) A surface potential map of CTSL is shown with CQ shown in stick representation. (B) CTSL is shown in cartoon representation, but CQ and the three catalytic residues are shown in stick representation.

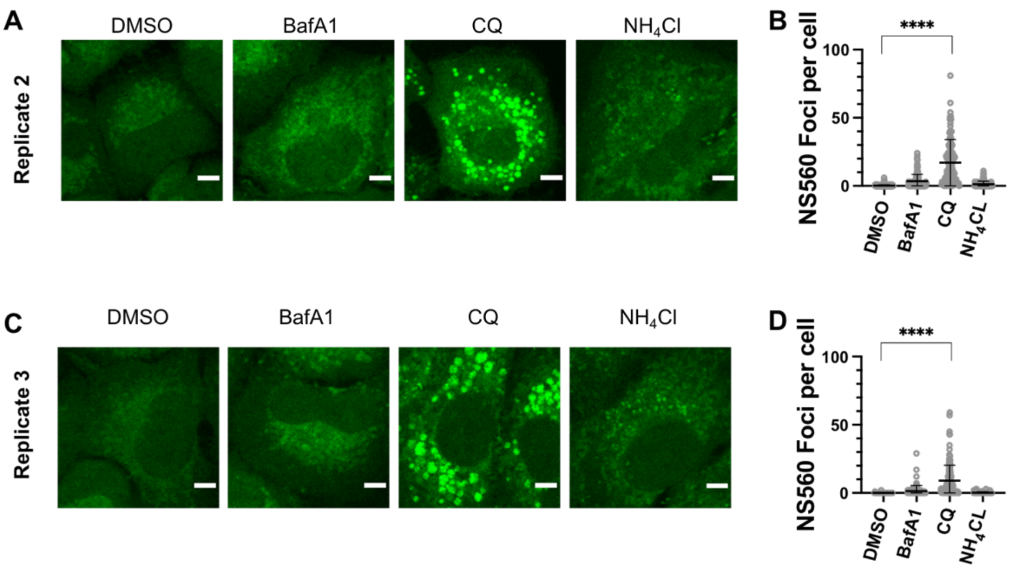

Fig S52. Fluorescent microscopy extended images for autophagy inhibitor screening. A549 cells treated with BafA1 (25 nM), CQ (25 µM), and NH_4Cl (25 mM) for 7 hours. Representative images of the final two biological replicates (A and C) and corresponding quantification (B and D). Scale bar, 5 µm.

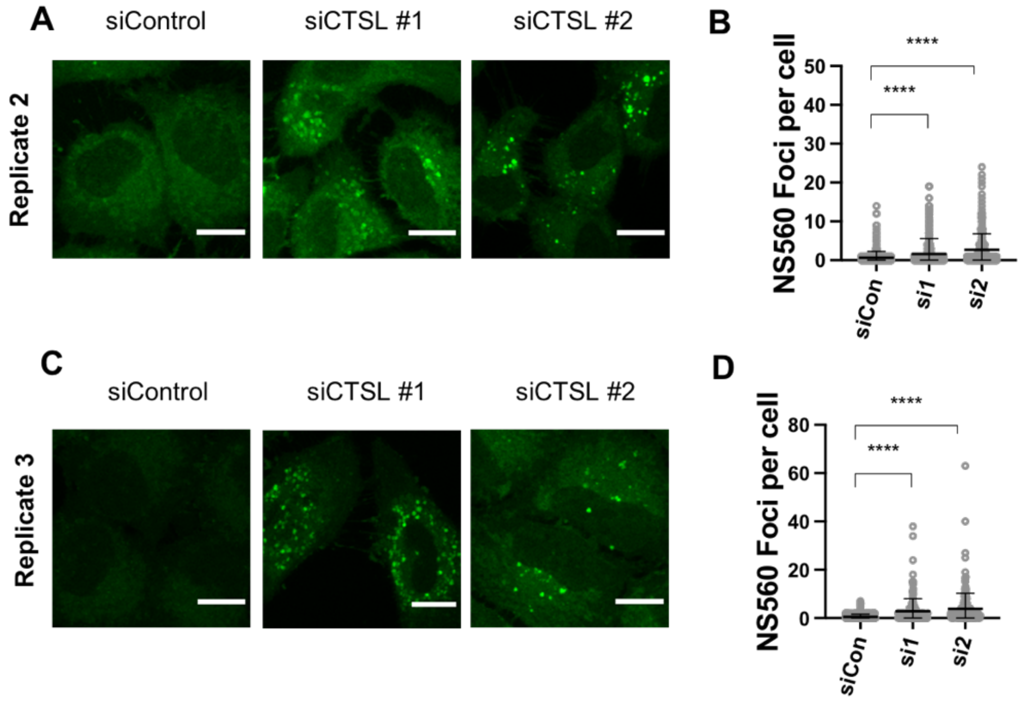

Fig S53. Fluorescent microscopy extended images for siCTSL. A549 cells were transfected with siRNA for CTSL for 48-72 hours then labeled with NS560 for 45 minutes. Representative images from remaining two biological replicates (A and C) along with quantification (B and D). Scale bar, 10 µm.

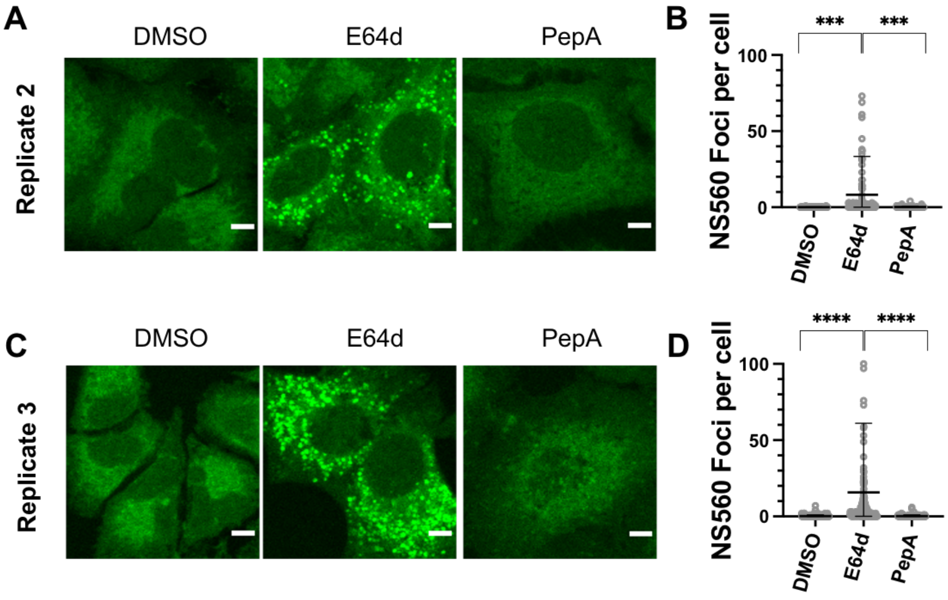

Fig S54. Fluorescent microscopy extended images for CTSL inhibition. A549 cells treated with E64d (25 µM) or Pepstatin A (25 µM) for 24 hours then labelled with NS560. Scale bar, 5 µm. (A and C) Representative quantification of NS560 foci per cell is shown for images in B and D. First biological replicate shown in Fig. 5F and quantification in 5G

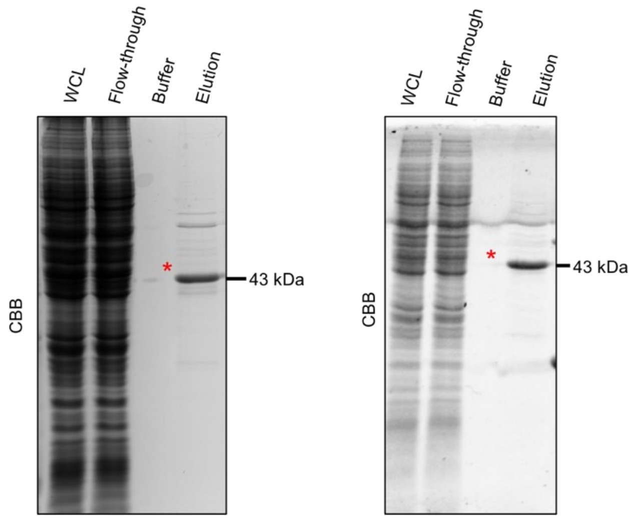

Figure S55. Representative SDS-gel images of purified CTSL. CTSL-Flag-Myc purified from HEK-293T cells using anti-Flag beads. Red star indicates CTSL.

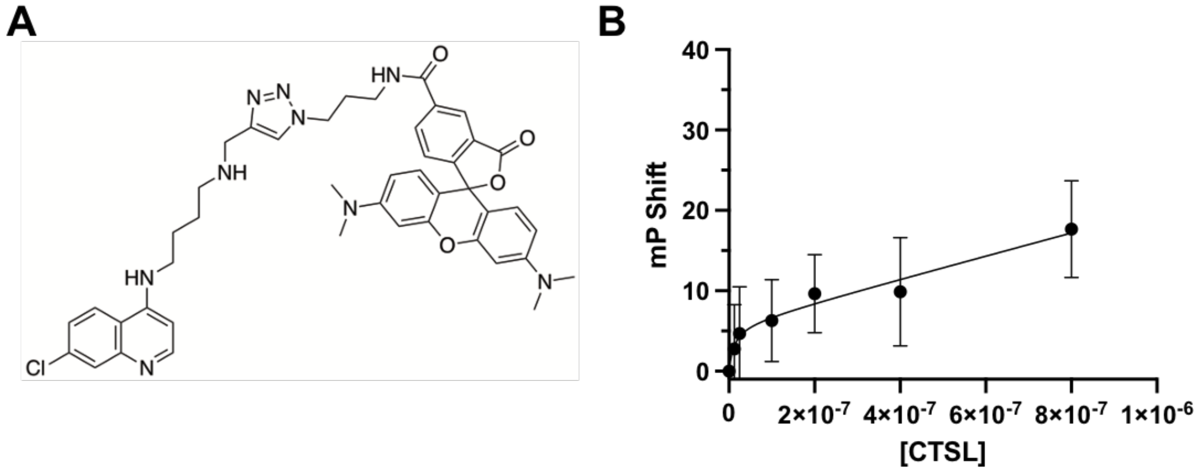

Figure S56. Titration of CTSL causes moderate mP shift of CQ-TAMRA. (A) Structure of CQ-TAMRA (B) mP shift values measured after 10-minute incubation of CQ-TAMRA with varying concentrations of CTSL. n =2.

Table S1. Association constants of NS560 binding to 20 proteogenic amino acids

Association constants of NS560 binding to 20 proteogenic amino acids

**Amino acid**	Ka(M^{-1}) pH = 5.0	Ka(M^{-1}) pH = 7.4
**Alanine**	9.625	45
**Arginine**	44.72	67
**Asparagine**	76.98	196
**Aspartic acid**	14.36	81
**Cysteine**	ND	870
**Glutamic acid**	23.17	44
**Glutamine**	80.06	94
**Glycine**	41.07	10
**Histidine**	148.5	264
**Isoleucine**	69.74	57
**Leucine**	65.92	35
**Lysine**	92.59	7.0
**Methionine**	125.0	150
**Phenylalanine**	225.3	25
**Proline**	ND	ND
**Serine**	106.4	230
**Threonine**	158.0	380
**Tryptophan**	ND	ND
**Tyrosine**	283.0	510
**Valine**	112.7	4.5
**Glutathione**	ND	103

Synthetic Procedures

Synthesis of NS560

All chemicals were obtained from Sigma Aldrich, Acros, Fisher, TCI America, Alfa Aesar, or Combi-Blocks and were used without further purification. Flash chromatography was performed with 32-63 µm silica gel. NMR spectra were recorded on a Bruker DRX 500 and 600. IR spectra were recorded on a Nexus 670 FT-IR E.S.P. spectrometer.

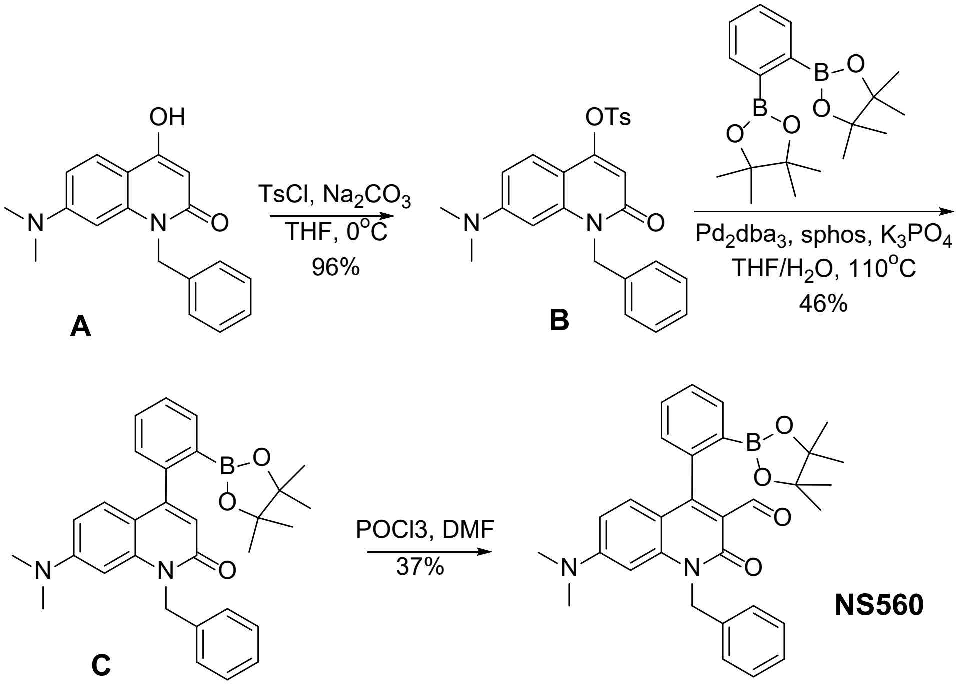

Compound **A**(2.94 g, 10 mmol) was mixed with THF (50 ml) and H_2O (2.5 ml) at ice/water baththen Na_2CO_3 (1.27 g, 12.0 mmol) was added into the cloudy mixture. The mixture was stirred at 0 °C for 15 min. *p*-Toluenesulfonyl chloride (2.29 g, 12.1 mmol) was added into the cloudy mixture and stirred it at 0 °C for 3 hrs. The mixture was warmed to room temperature and quenched with H_2O, and extracted with CH_2Cl_2 (3 x 20 mL), dried on $MgSO_4$, and the solvent was removed *in vacuo*. The resulting residue was purified by chromatography (2:1 Hex/EtOAc) to give compound **B** as yellow powder (4.3 g, 97%) M.P. 151-154 °C. ^1H NMR (600 MHz, $CDCl_3$) δ 7.86 (d, 2H, J = 8.4 Hz), 7.55 (d, 1H, J = 9 Hz), 7.32 (d, 2H, J = 8.4 Hz), 7.26 (t, 2H, J = 5.4 Hz), 7.18-7.25 (m, 3H), 6.50 (dd, 1H, J = 9, 2.4 Hz), 6.22 (d, 2H, J = 2.4 Hz), 5.41 (s, 2H), 2.87 (s, 6H), 2,41 (s, 3H); ^{13}C NMR (150 MHz, $CDCl_3$) δ 163.4, 155.2, 152.5, 146.1, 141.4, 136.6, 132.5, 130.2, 128.8, 128.4, 127.3, 126.8, 124.6, 108.3, 106.2, 104.4, 96.2, 46.1, 40.1, 21.8; IR (neat, cm^{-1}) 3060.6, 2993.2, 2683.1, 2305.6, 1649.4, 1622.4, 1595.5, 1528.0, 1402.2, 1271.9, 1191.0, 1164.0, 1065.1, 1029.2, 975.2; HRMS calculated for $C_{25}H_{24}N_2O_4SNa$ ($M+Na^+$): 471.1354, found 471.1349.

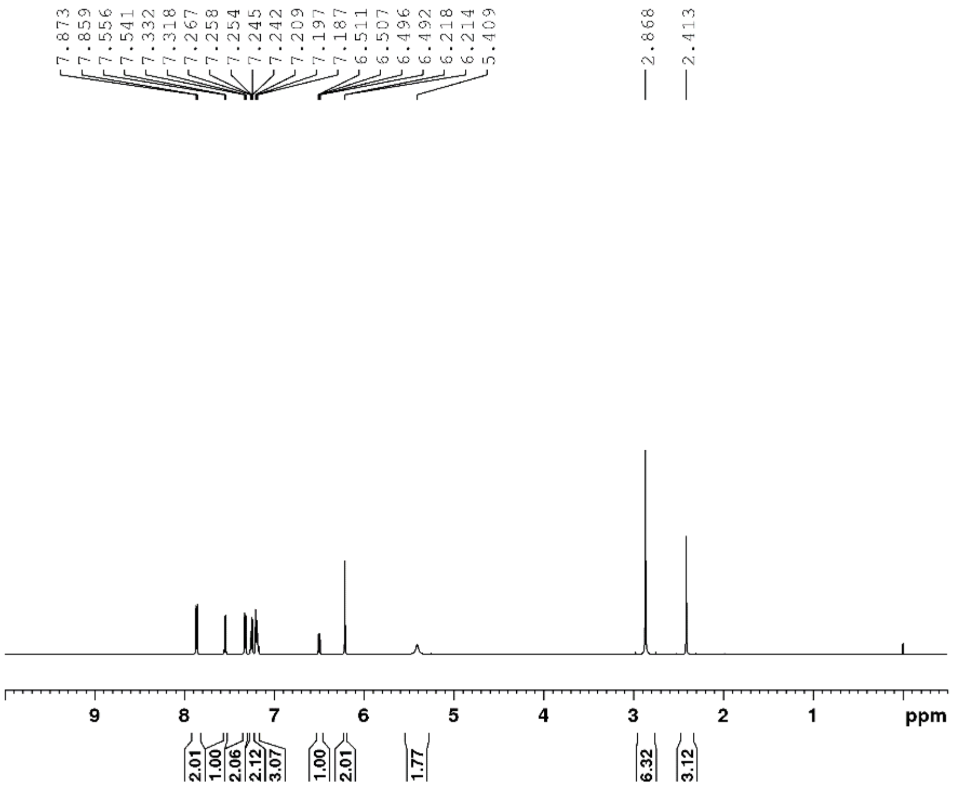
7.873
7.859
7.556
7.541
7.332
7.318
7.267
7.258
7.254
7.245
7.242
7.209
7.197
7.187
6.511
6.507
6.496
6.492
6.218
6.214
5.409
2.868
2.413
9
8
7
6
5
4
3
2
1
ppm
2.01
1.00
2.06
2.12
3.07
1.00
2.01
1.77
6.32
3.12

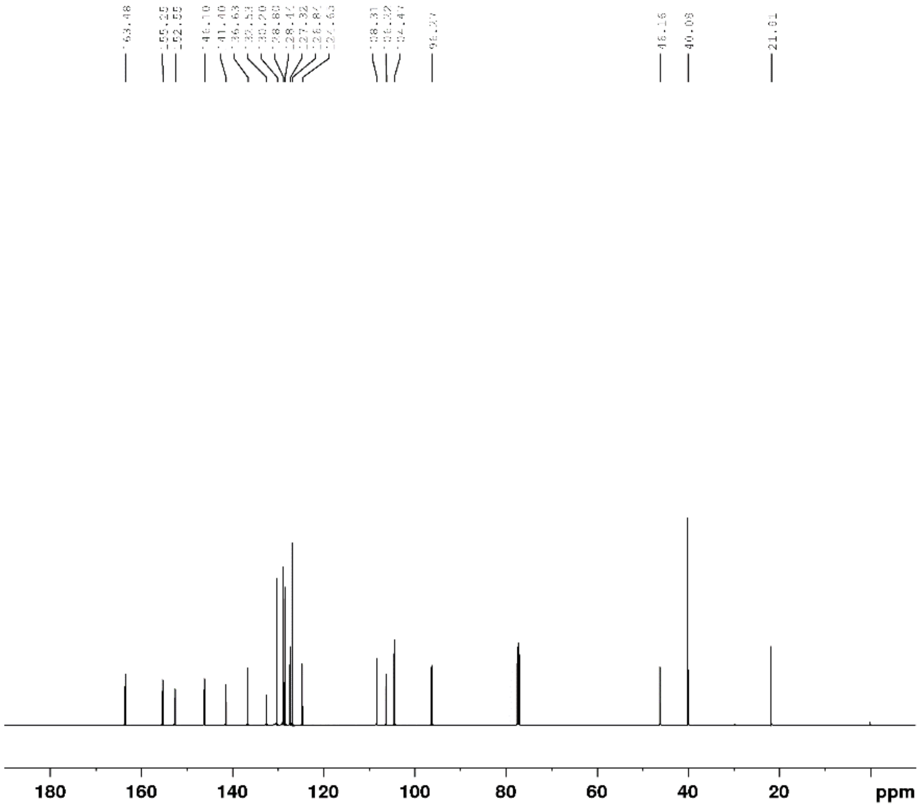
180
160
140
120
100
80
60
40
20
ppm

Compound **B** (1.2 g, 3 mmol), 2,2-(1,2-Phenylene)bis[4,4,5,5-tetramethyl-1,3,2-dioxaborolane] (1.08 g, 3.27 mmol), Pd_2dba_3 (137.4 mg, 0.15 mmol), Sphos (184.7 mg, 0.45 mmol) and K_3PO_4 (1.27 g, 6 mmol) were placed in a sealed tube with THF (15 ml) and H_2O (0.75 ml). After the mixture was degassed for 20 min, it was heated to 110 °C for 36 hrs. The solution was filtered and extracted with CH_2Cl_2 (3×50ml) and removed *in vacuo*. The remaining residue was purified by flash chromatography (80:1 CH_2Cl_2/MeOH, 40:1 CH_2Cl_2/MeOH) to furnish compound **C** as a light yellow amorphous solid (1.15 g, 96%). ^1H NMR (500 MHz, $CDCl_3$) δ 7.83 (dd, 1H, J = 7, 0.5 Hz), 7.52 (td, 1H, J = 7.5, 1 Hz), 7.43 (td, 1H, J = 7.5, 1 Hz), 7.26-7.35 (m, 5H), 7.22 (t, 1H, J = 7 Hz), 7.03 (d, 1H, J = 9 Hz), 6.45 (s, 1H), 6.40 (dd, 1H, J = 9, 2.5 Hz), 6.36 (d, 1H, J = 2.5 Hz), 5.93 (s, 1H), 5.29 (s, 1H), 2.87 (s, 6H), 1.09 (s, 1H), 0.89 (s, 6H); ^{13}C NMR (125 MHz, $CDCl_3$) δ 163.2, 153.1, 151.6, 143.4, 140.8, 137.4, 134.7, 130.7, 128.8, 128.7, 127.5, 127.1, 126.9, 115.7, 113.5, 107.8, 97.1, 83.6, 45.8, 40.3, 24.6, 24.4; IR (neat, cm^{-1}) 3433.7, 3056.1, 2984.2, 2692.1, 2305.6, 1658.4, 1613.4, 1541.4, 1429.2, 1406.7, 1352.8, 1276.4, 1146.0, 894.3; HRMS calculated for $C_{30}H_{33}BN_2O_3Na$ (M+Na^+): 503.2482, found 503.2477.

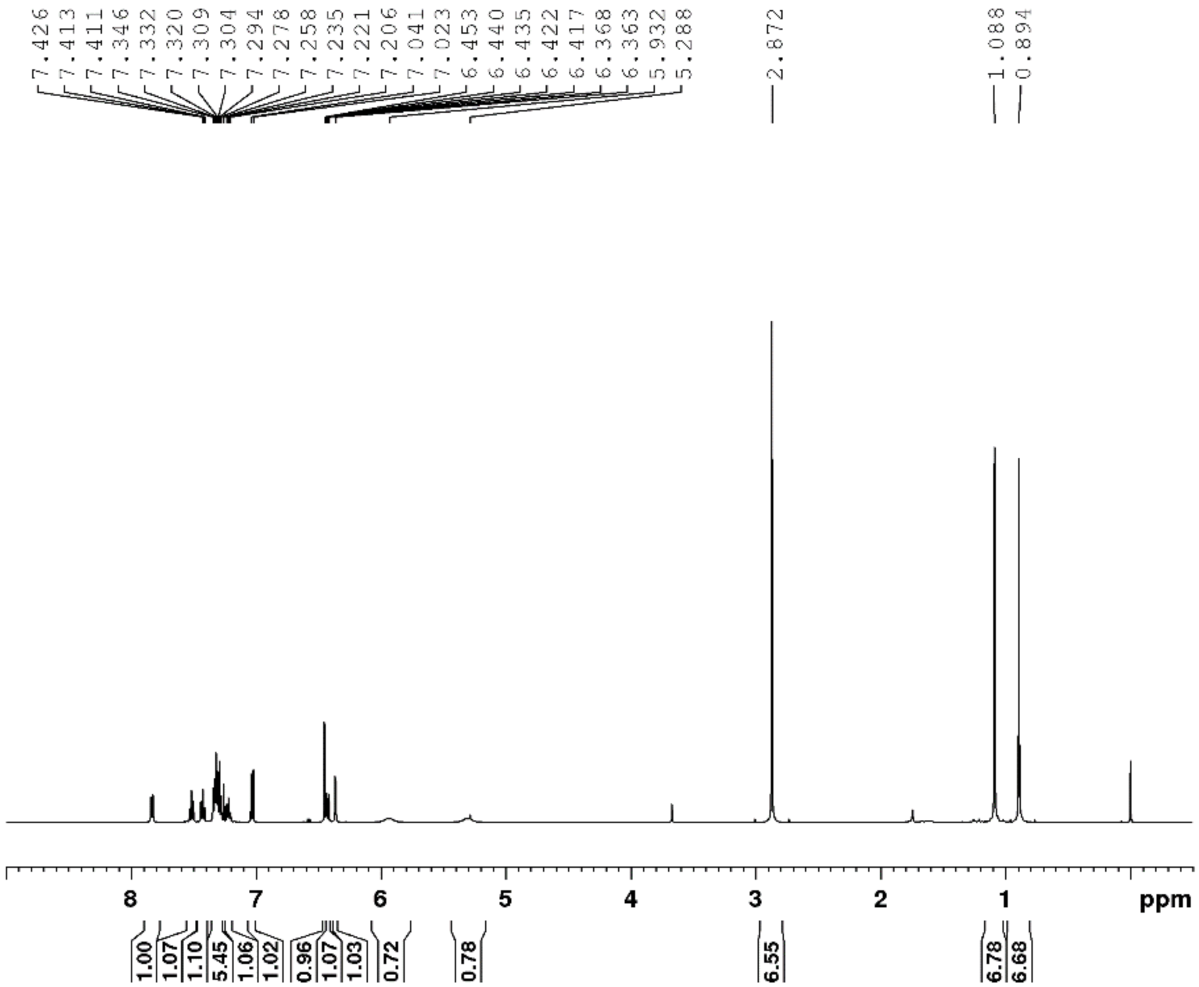

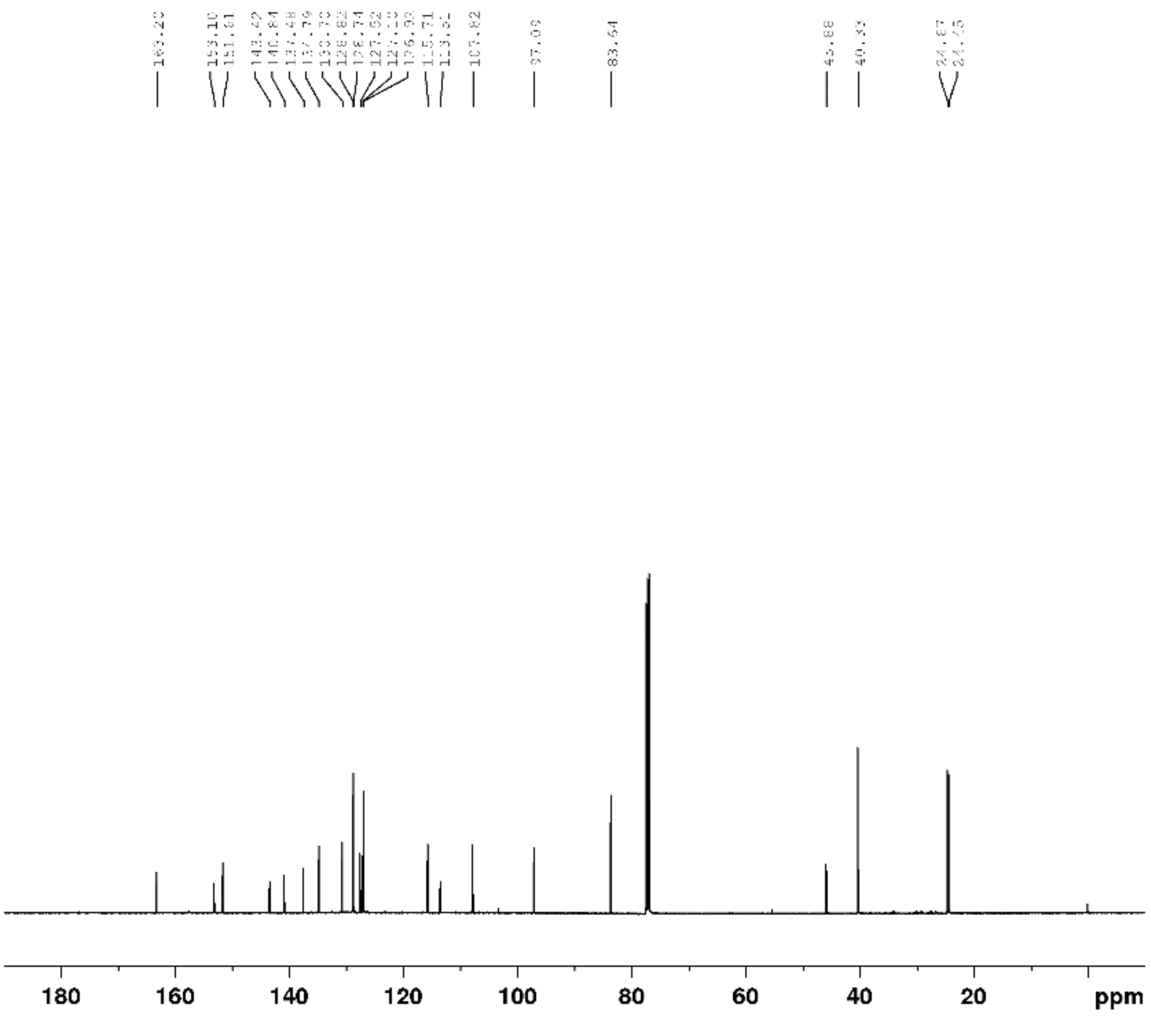

$POCl_3$ (0.37 ml) was added to DMF (0.8 ml) at 0 ºC in a flame dried round bottom flask. The mixture was stirred at ambient temperature for 1 hours. The Vilsmeier reagent (0.12 ml) was added to a solution of compound **C** (144.1 mg, 0.3 mmol) in 4 ml DMF. The solution was stirred at ambient temperature for 16 hours. The resulting red solution was poured onto ice water, extracted with CH_2Cl_2 (3×10ml). The combined organic layers were dried over Na_2SO_4 and the solvent was removed *in vacuo*. The residue was purified by chromatography (80:1 CH_2Cl_2/MeOH) to yield **NS560** (56.4 mg, 37%) as dark yellow foaming solids. ^{1}H NMR (500 MHz, $CDCl_3$) δ 10.18 (s, 1H), 7.93 (dd, 1H, J = 7, 0.5 Hz), 7.54 (td, 1H, J = 7.5, 1.5 Hz), 7.45 (td, 1H, J = 7.5, 1 Hz), 7.36 (d, 2H, J = 7 Hz), 7.32 (t, 2H, J = 2 Hz), 7.25 (d, 1H, J = 8.5 Hz), 7.19 (d, 1H, J = 7 Hz), 6.85 (d, 1H, J = 9 Hz), 6.38 (dd, 1H, J = 9.5, 2.5 Hz), 6.27 (d, 1H, J = 2.5 Hz), 5.97 (s, 1H), 5.29 (s, 1H), 2.93 (s, 6H), 1.06 (s, 6H), 0.89 (s, 6H); ^{13}C NMR (125 MHz, $CDCl_3$) δ 190.8, 162.4, 158.4, 153.2, 142.7, 141.5, 137.0, 135.3, 131.5, 130.5, 128.8, 128.5, 127.4, 127.3, 127.0, 117.9, 112.8, 108.4, 95.7, 83.5, 45.8, 40.1, 24.7, 24.4; IR (neat, cm^{-1}) 3438.2, 3056.1, 2984.2, 2310.1, 1703.3, 1604.4, 1528.0, 1483.1, 1424.7, 1352.8, 1267.4, 1164.0, 1146.0; HRMS calculated for $C_{31}H_{33}BN_2O_4Na$ (M+Na^{+}): 531.2431, found 531.2421.

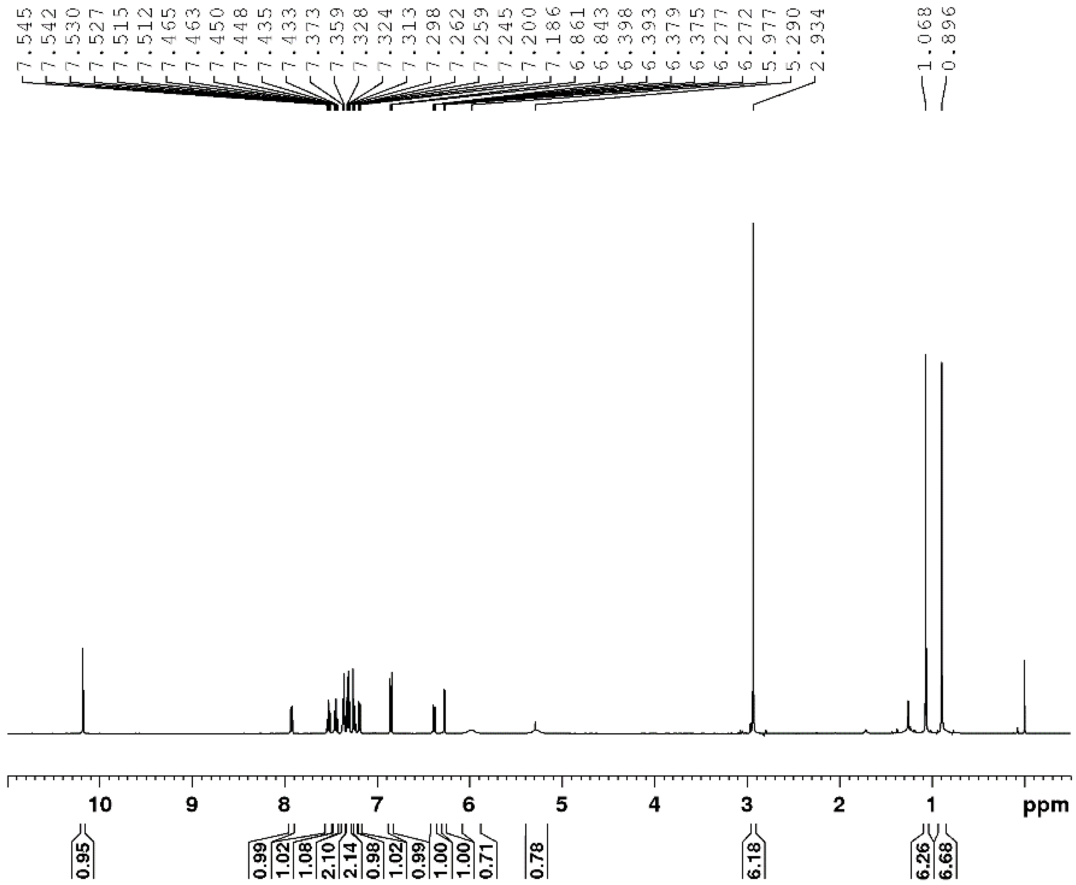

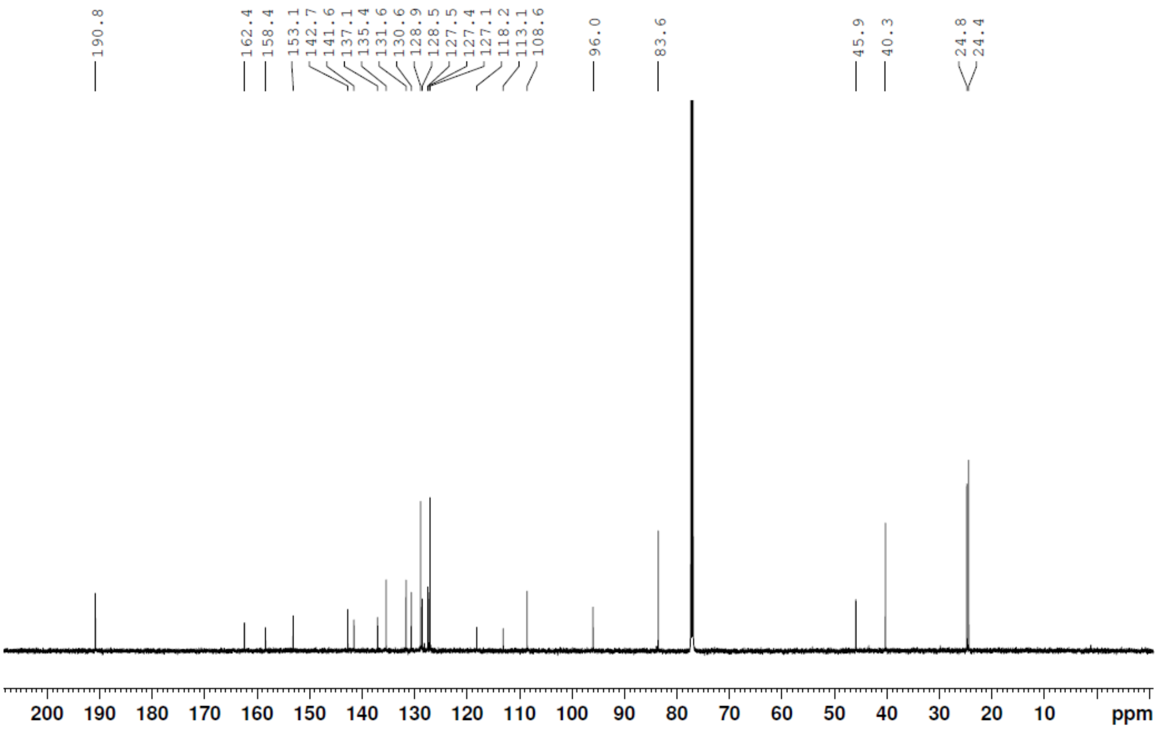

Chloroquine crosslinker CQ-X:

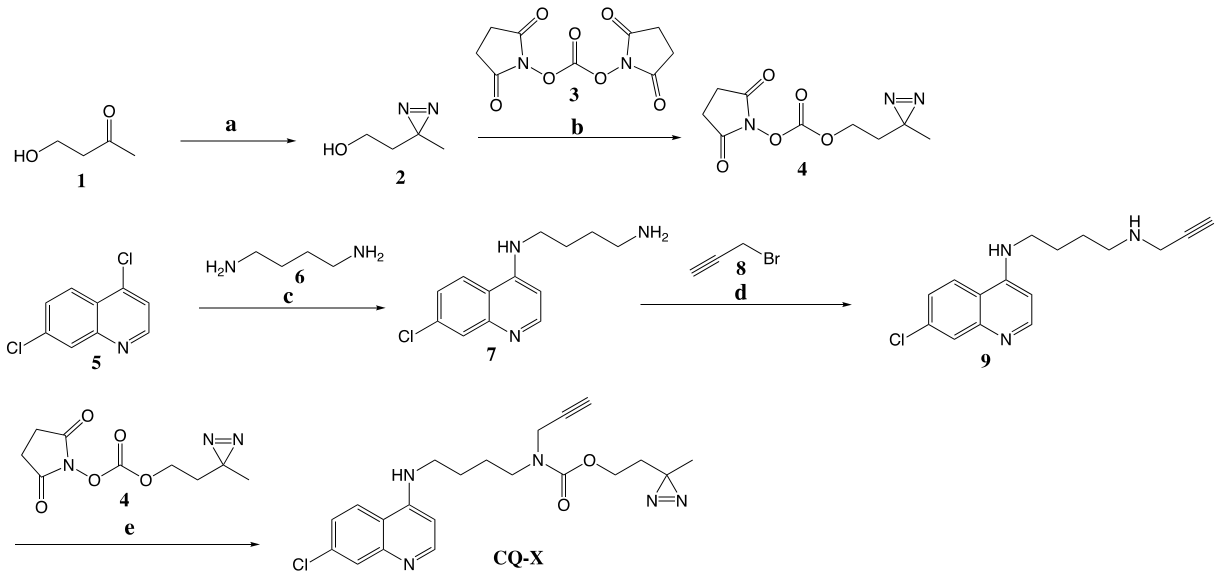

2-(3-methyl-3*H*-diazirin-3-yl)ethan-1-ol (2): To liquid NH_3 (110mL) was added 4-hydroxybutan-2-one (**1**, 17.7g, 0.20mol) slowly, then it was stirred at -78°C for 3 hrs. A solution of NH_2OSO_3H (25 g, 0.22 mol) in methanol (180 ml) was added. The dry-ice bath was removed, the solution was stirred at room temperature for overnight. After filtration, the solid was washed by methanol (5 mL). The combined methanol solution was cooled to 0°C and 27 mL of triethylamine (0.94 mmol) and I_2 (25.2g, 0.10 mol) was added slowly. The result solution was stirred at room temperature for 2 hrs. Brine (400 mL) was added and solution was extracted by ether (3 x 100 mL). The combined ether solution was dried and evaporated. The residue was purified by column (Hexane:Ethyl acetate = 6:1) to afford compound **2** (6.1g, 33% yield) as colorless oil. ^{1}H NMR ($CDCl_3$): **δ** 1.06(s, 3H), 1.62 (t, J6.0Hz, 3H), 3.52 (q, J6.0Hz, 2H).

2,5-dioxopyrrolidin-1-yl (2-(3-methyl-3*H*-diazirin-3-yl)ethyl) carbonate (4): A solution of DSC (**3,** 3.84 g, 14.98 mmol) in MeCN (10 mL) was added to a solution of 2-(3-methyl-3*H*-diazirin-3-yl)ethan-1-ol (**2**, 1.00 g, 9.99 mmol) and TEA (1.39 mL, 36.96 mmol) in MeCN (10 mL). The reaction solution was stirred at room temperature overnight. Solvents were removed under vacuum and the residue was purified by column (Hexane:Ethyl acetate = 3:1) to afford compound **4** (2.06 g, 85% yield) as white solid. ^{1}H NMR ($CDCl_3$): **δ** 1.09 (s, 3H); 1.77 (t, J6.0Hz, 2H), 2.85 (s, 4H), 4.27 (t, J6.0Hz, 2H).

***N*¹-(7-chloroquinolin-4-yl)butane-1,4-diamine (7):** A mixture of 4,7-dichloroquinoline (4 g, 20.20 mmol) and butane-1,4-diamine (8.9 g, 100.98 mmol) was heated to 80°C and stirred for 30 mins. Then the temperature was raised to 110°C and stirred for 6 hrs. Then the reaction was cooled down to room temperature and 50 mL of ethyl acetate and water were added. After stirring and separating the organic and aqueous layers, the organic layer was washed and dried. After concentration, the residue was purified by column (DCM : Methanol = 30 : 1) to afford compound **7** (2.26 g, 45% yield) as brown solid. LC-MS: Calcd 249.10, Found 250.22.

N^1-(7-chloroquinolin-4-yl)-N^4-(prop-2-yn-1-yl)butane-1,4-diamine (9): To a solution of N^1-(7-chloroquinolin-4-yl)butane-1,4-diamine (**7**.1 g, 6.71 mmol) in EtOH (40 mL) was added propargyl bromide (**8**, 0.71 mL, 8.00 mmol) and K_2CO_3 (1g, 7.23mmol). The mixture was stirred at room temperature for 12 hrs. Solvent was removed under vacuum and the residue was dissolved in ethyl acetate (80 mL) and washed with water (100 mL) and brine (80 mL) and dried. The residue was purified by column (DCM: Methanol = 40:1) to afford compound **9** (1.01 g, 52% yield) as white solid. LC-MS: Calcd 287.79, Found 288.51.

2-(3-methyl-3*H*-diazirin-3-yl)ethyl (4-((7-chloroquinolin-4-yl)amino)butyl)(prop-2-yn-1-yl)carbamate (CQ-X): To a solution of N^1-(7-chloroquinolin-4-yl)-N^4-(prop-2-yn-1-yl)butane-1,4-diamine (**9**, 50 mg, 0.1737 mmol) and 2,5-dioxopyrrolidin-1-yl (2-(3-methyl-3*H*-diazirin-3-yl)ethyl) carbonate (**4,** 38.08 mmol) in DCM (5ml) and DMF (1.5mL) was added DIPEA (30 µL, 0.1737 mmol). The solution was stirred at room temperature for 7 hrs, and then diluted with DCM (20 mL) and washed with water (30 mL) and brine (30 mL), dried and evaporated. The residue was purified by column (DCM:Methanol= 30:1) to afford CQ-X (47.46 mg, 66%) as white solid ^{1}H NMR (DMSO): **δ** 1.09 (3H, s), 1.39-1.60 (m, J6.0Hz, 7.1Hz, 6H), 2.87 (t, J3.0Hz, 2H), 3.01(s, 1H), 3.35(t, J7.1Hz, 2H), 3.49 (s, 2H), 4.11(t, J6.0Hz, 2H), 6.47 (d, J10.1Hz, 1H), 7.35 (s, 1H), 7.45 (d, J10.1Hz, 1H), 7.77 (s, 1H), 8.26(d, J 10.1Hz, 1H), 8.27 (d, 10.1Hz, 1H) LC-MS: Calcd 413.97, Found 414.76.

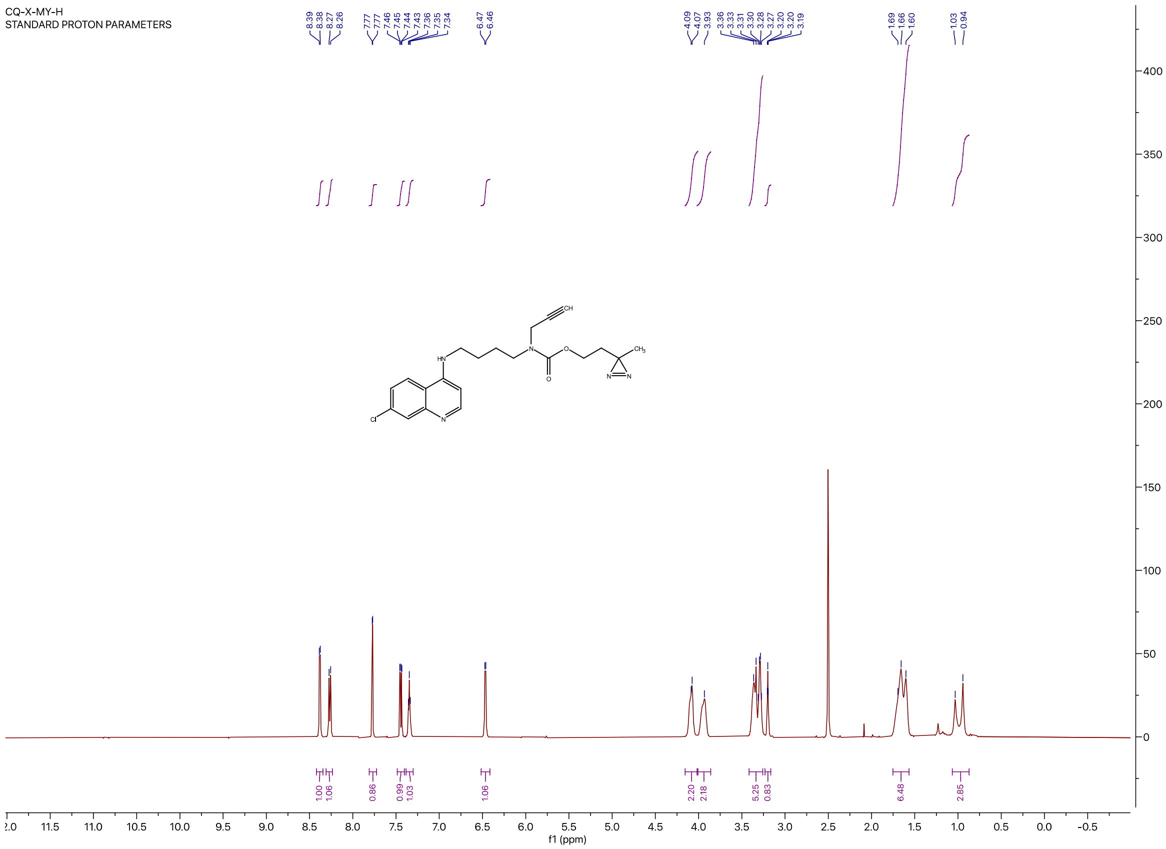

^{1}H NMR of CQ-X

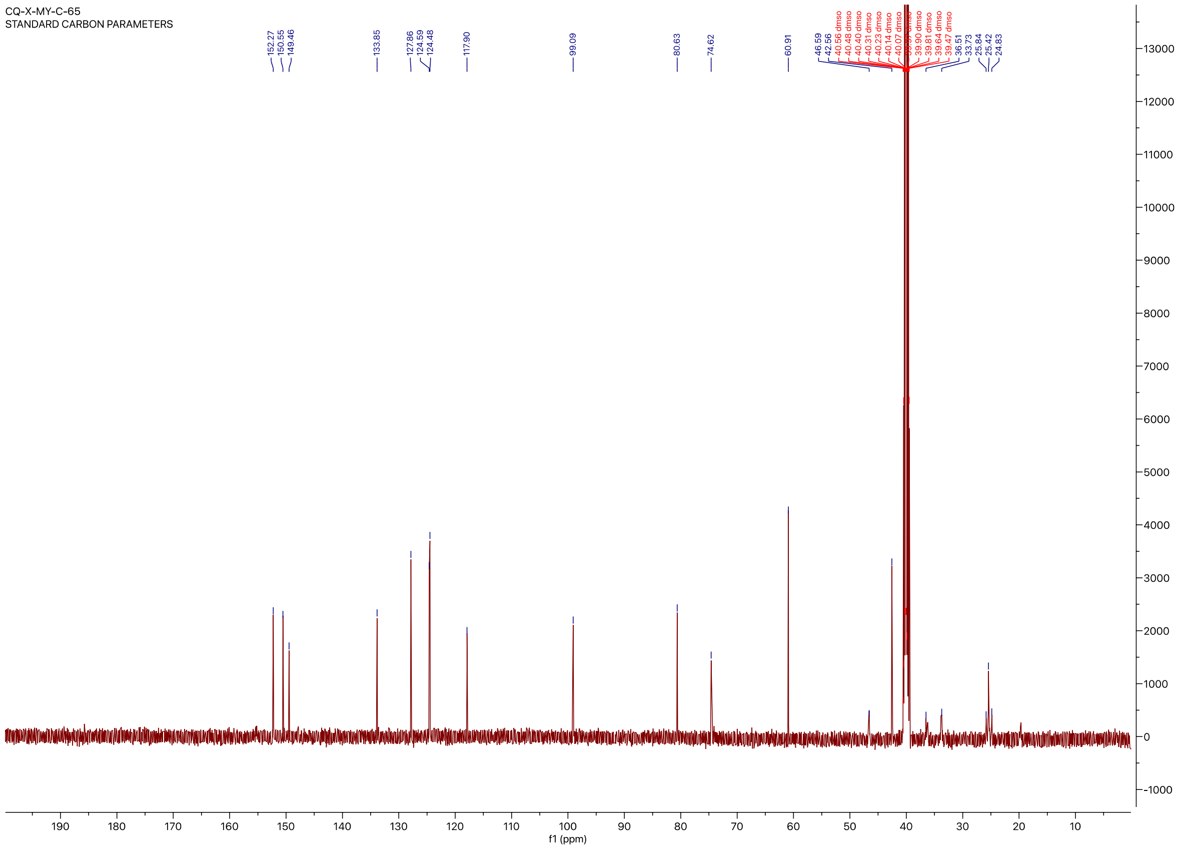

^{13}C NMR of CQ-X

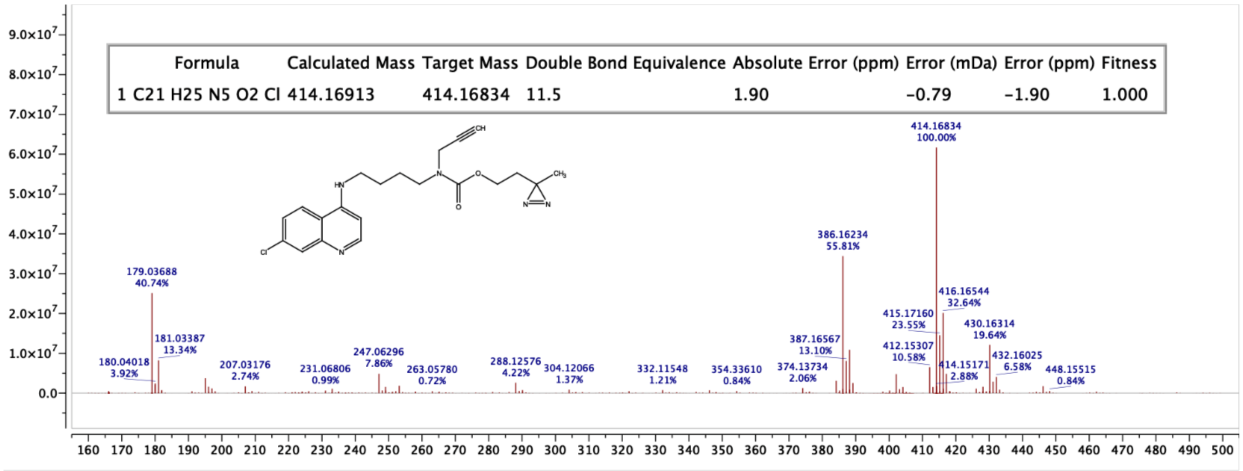

HRMS of CQ-X

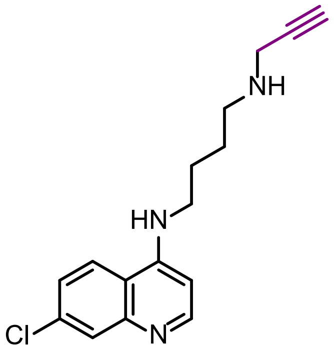

N1-(7-chloroquinolin-4-yl)-N4-(prop-2-yn-1-yl)butane-1,4-diamine **(compound 9)**

NMR (500 MHz, MeOD) δ 8.33 (d, *J* = 5.6 Hz, 1H), 8.08 (d, *J* = 9.0 Hz, 1H), 7.76 (d, *J* = 2.2 Hz, 1H), 7.37 (dd, *J* = 9.0, 2.2 Hz, 1H), 6.48 (d, *J* = 5.7 Hz, 1H), 3.40 (d, *J* = 2.5 Hz, 2H), 3.36 (t, *J* = 7.0 Hz, 2H), 2.73 (t, *J* = 7.3 Hz, 2H), 2.62 (t, *J* = 2.5 Hz, 1H), 1.83 – 1.73 (m, 2H), 1.70 – 1.60 (m, 2H). ^{13}C NMR (126 MHz, MeOD) δ 151.36, 150.85, 148.08, 134.94, 126.05, 124.55, 122.96, 117.32, 98.22, 80.63, 71.88, 48.14, 48.08, 47.97, 47.80, 47.63, 47.46, 47.29, 47.12, 42.45, 37.03, 26.44, 25.76. LCMS (M+H): C16H19ClN3 calculated 288.2, observed 288.2.

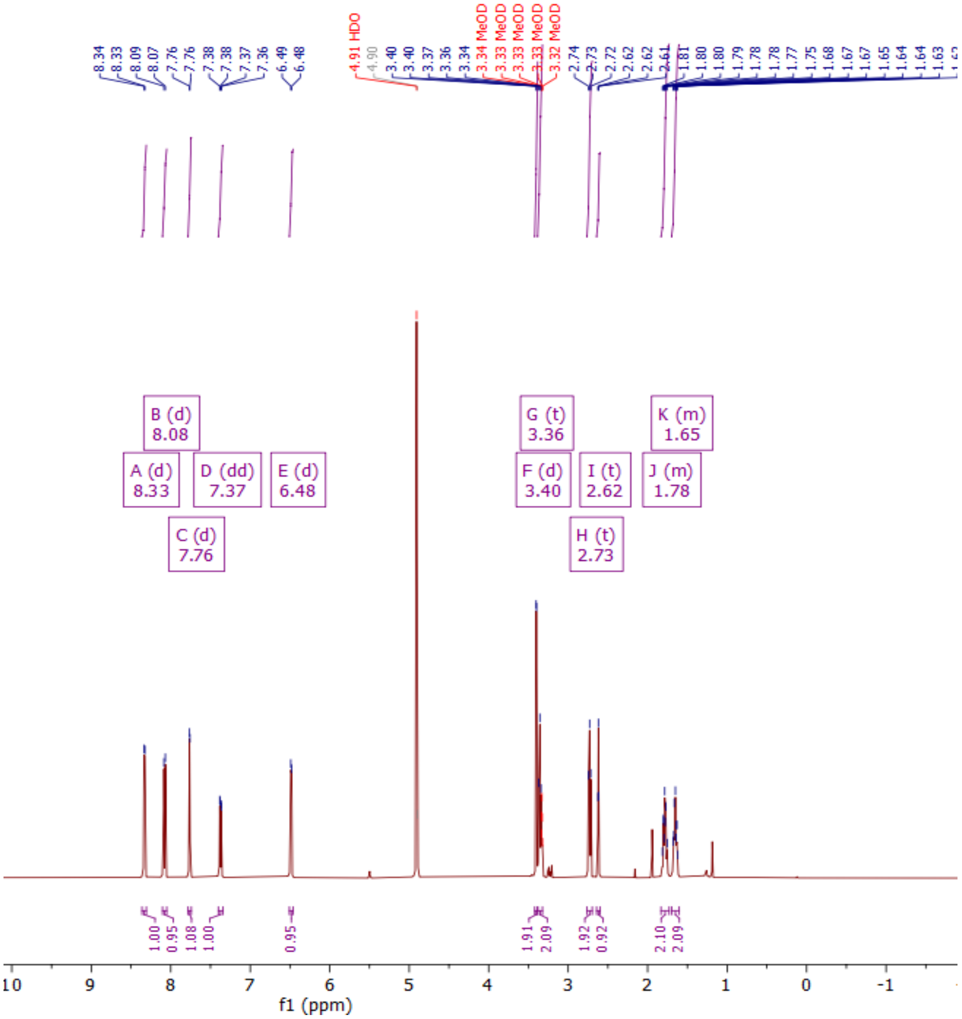

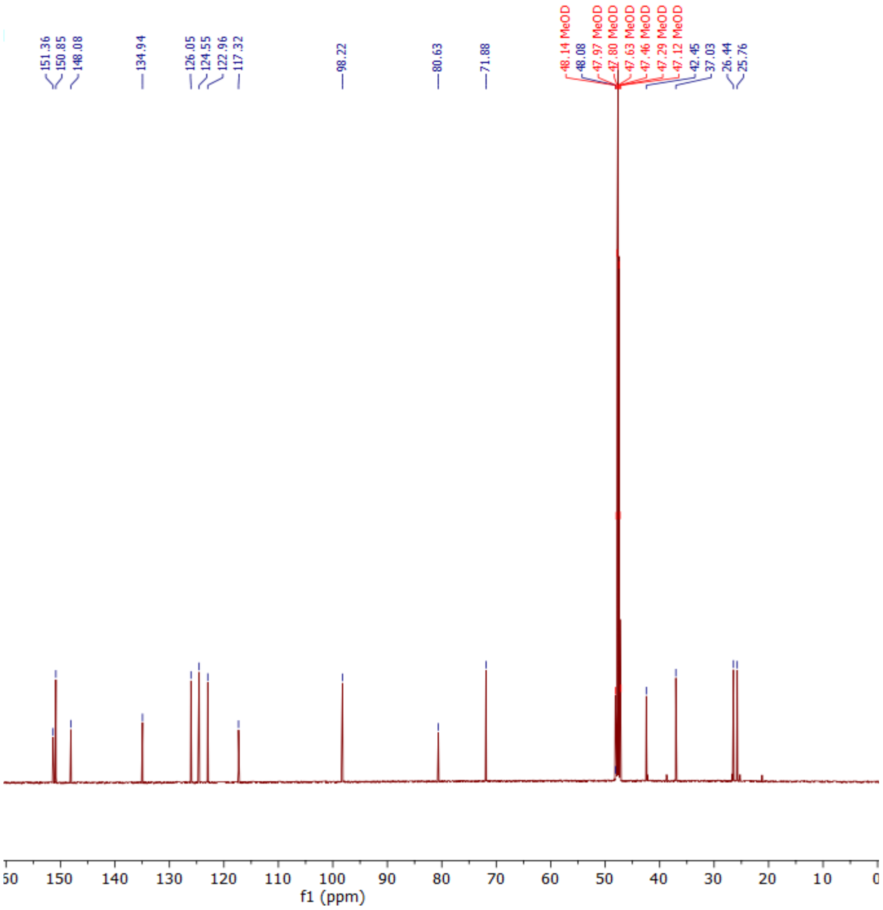
151.36
150.85
148.08
134.94
126.05
124.55
122.96
117.32
98.22
80.63
71.88
48.14 MeOD
48.08
47.97 MeOD
47.80 MeOD
47.63 MeOD
47.46 MeOD
47.29 MeOD
47.12 MeOD
42.45
37.03
26.44
25.76
f1 (ppm)

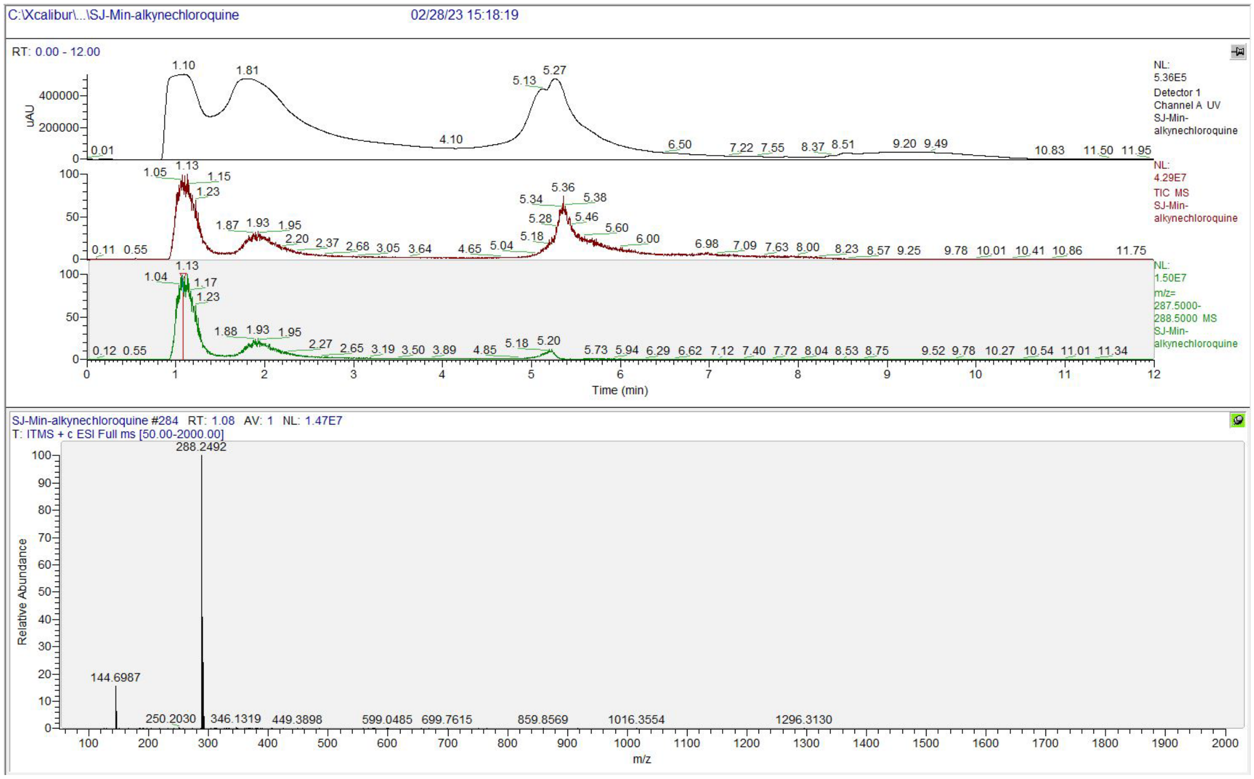
C:\Xcalibur\...\SJ-Min-alkynechloroquine
02/28/23 15:18:19
RT: 0.00 - 12.00
NL: 5.36E5 Detector 1 Channel A UV SJ-Min-alkynechloroquine
NL: 4.29E7 TIC MS SJ-Min-alkynechloroquine
NL: 1.50E7 m/z= 287.5000-288.5000 MS SJ-Min-alkynechloroquine
uAU
Time (min)
SJ-Min-alkynechloroquine #284 RT: 1.08 AV: 1 NL: 1.47E7
T: ITMS + c ESI Full ms [50.00-2000.00]
288.2492
144.6987
250.2030
346.1319
449.3898
599.0485
699.7615
859.8569
1016.3554
1296.3130
Relative Abundance
m/z

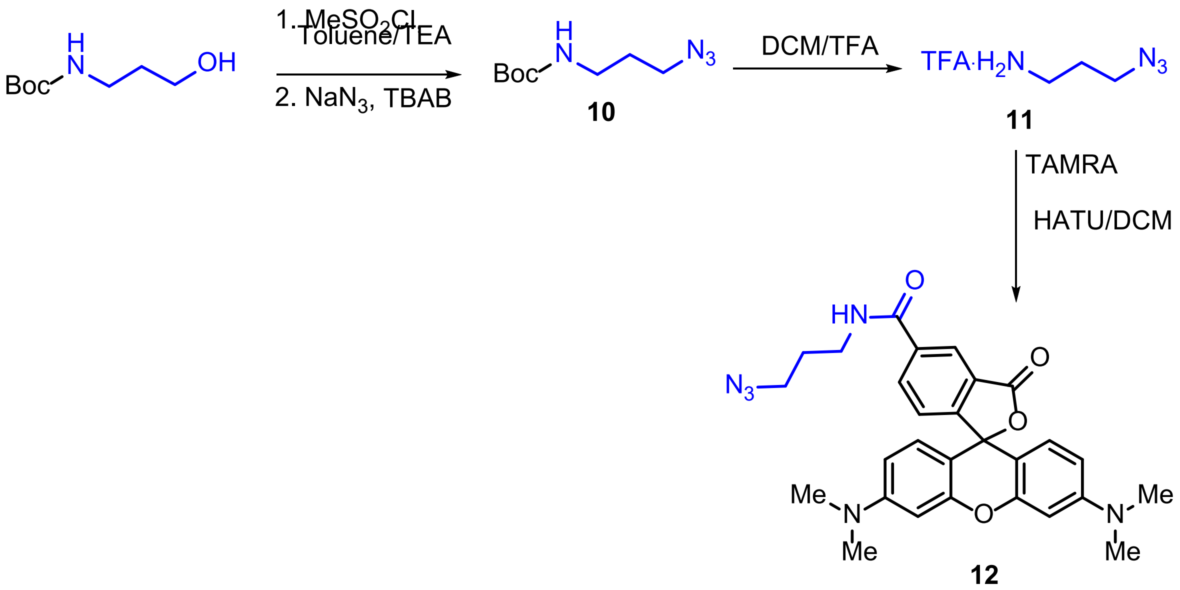

Procedure:

Step 1: To a solution of compound **10** (1.0 equiv. 5.707 mmol, 1000 mg) in toluene (40 mL) at 0 °C was added triethylamine (TEA) and then $MeSO_2Cl$. After, stirring for 30 min, tetra butyl ammonium bromide and NaN_3 (20% water) was added to the reaction mixture and stirrer at 80 °C for 7 hours, cooled and added 250 mL of diethyl ethyl. The organic layer was washed with water and brine. The solvent was evaporated, and the crude residue was purified by silica gel column chromatography using hexane/ethyl acetate as elute to give compound **11** (707 mg, 62%)

Step II: Deprotection of compound **11**: The compound **11** was dissolved in 10 mL of dichloromethane and 5 mL of trifluoroacetic was added to this solution. The reaction mixture was stirred at room temperature for 2 hours. The crude reaction was check in LCMS to confirm the desired product and then solvent was removed by rotavapor. The residue was dissolved in in methanol and evaporated by rotavapor. The process was repeated by 6-7 times. The reside was dried over high vacuum overnight and proceed for next step without purification.

Step III: 5-carboxyteramethylrhodamine (5-TAMRA) (20 mg, 0.0465 mmol) was dissolved in 2 mL DMF and mixed with HATU (19.5 mg, 0.051 mmol). The reaction mixture was stirrer for 10 min to ensure it was fully dissolved **11** TFA salt (10.96 mg, 0.052 mmol) was then added, followed by N, N-diisopropylethylamine (DIPEA) (40.5 uL, 0.2325 mmol). The reaction mixture was stirred for overnight and DMF was vacuum concentrated upon completion by TLC. The crude reaction mixture was purified by column chromatography (DCM: Methanol: TEA= 100:10:0.2) to pink solid compound **12** (26 mg, 42%)

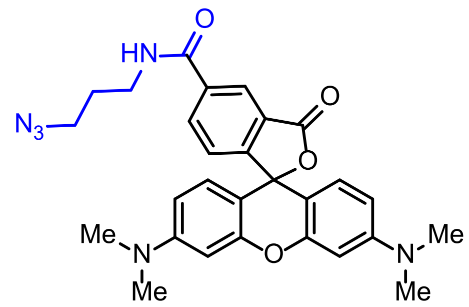

N-(3-azidopropyl)-3',6'-bis(dimethylamino)-3-oxo-3H-spiro[isobenzofuran-1,9'-xanthene]-5-carboxamide **(compound 12)**

^{1}H NMR (500 MHz, MeOD) δ 8.62 (d, J = 4.42. 2H), 8.31 (d, J = 8.44, 2H), 7.44-7.42 (m, 2H), 7.19 (d, J = 9.08, 1H), 7.04 (d, J = 9.08 Hz, 1H), 6.96 (s, 1H), 3.91-3.86 (m, 1H), 3.57 (t, J = 7.12 Hz, 1H), (t, J = 6.9 Hz, 1H), 3.44-3.41 (m, 1H), 3.39-3.37 (m, 1H), 3.31 (s, 6H), 3.27 (s, 6H), 13C NMR (126 MHz, MeOD) δ 161.83, 161.56, 157.64, 157.44, 148.59, 139.13, 134.98, 130.92, 127.79, 120.15, 117.90, 115.57, 113.88, 113.44, 95.96, 48.89, 46.50, 39.52, 37.23, 29.34, 28.39, 25.82, 17.90, 11.77. LCMS (M+H): $C_{28}H_{29}N_6O_4$ calculated 513.2, observed 513.3.

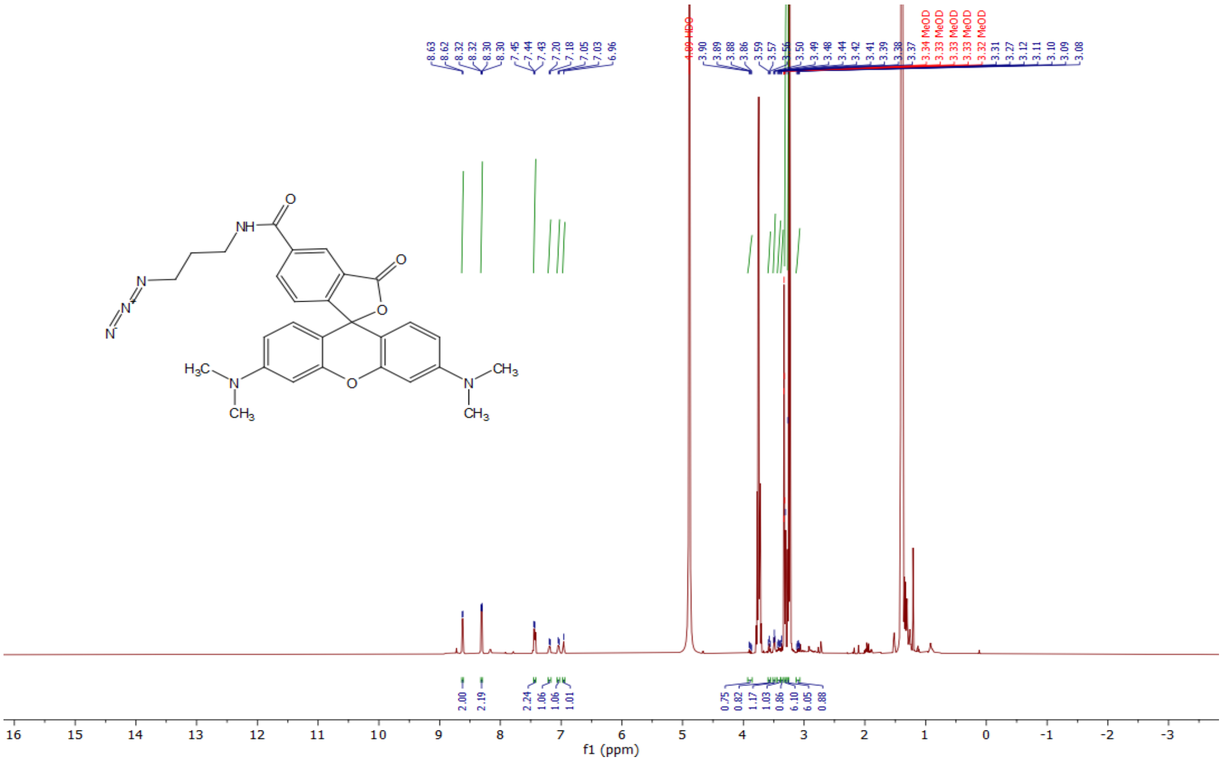

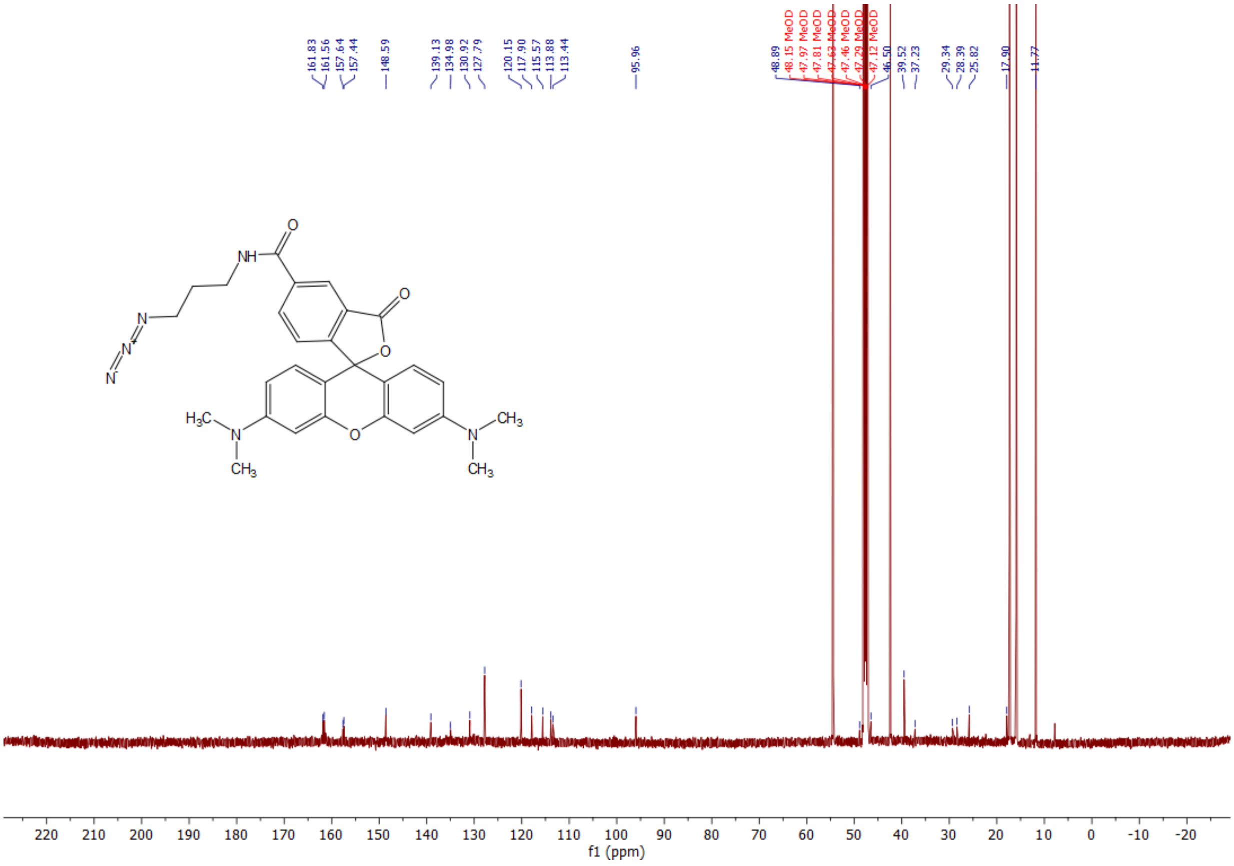

161.83
161.56
157.64
157.44
148.59
139.13
134.98
130.92
127.79
120.15
117.90
115.57
113.88
113.44
95.96
48.89
48.15 MeOD
47.97 MeOD
47.81 MeOD
47.63 MeOD
47.46 MeOD
47.29 MeOD
47.12 MeOD
46.50
39.52
37.23
29.34
28.39
25.82
17.90
11.77
f1 (ppm)

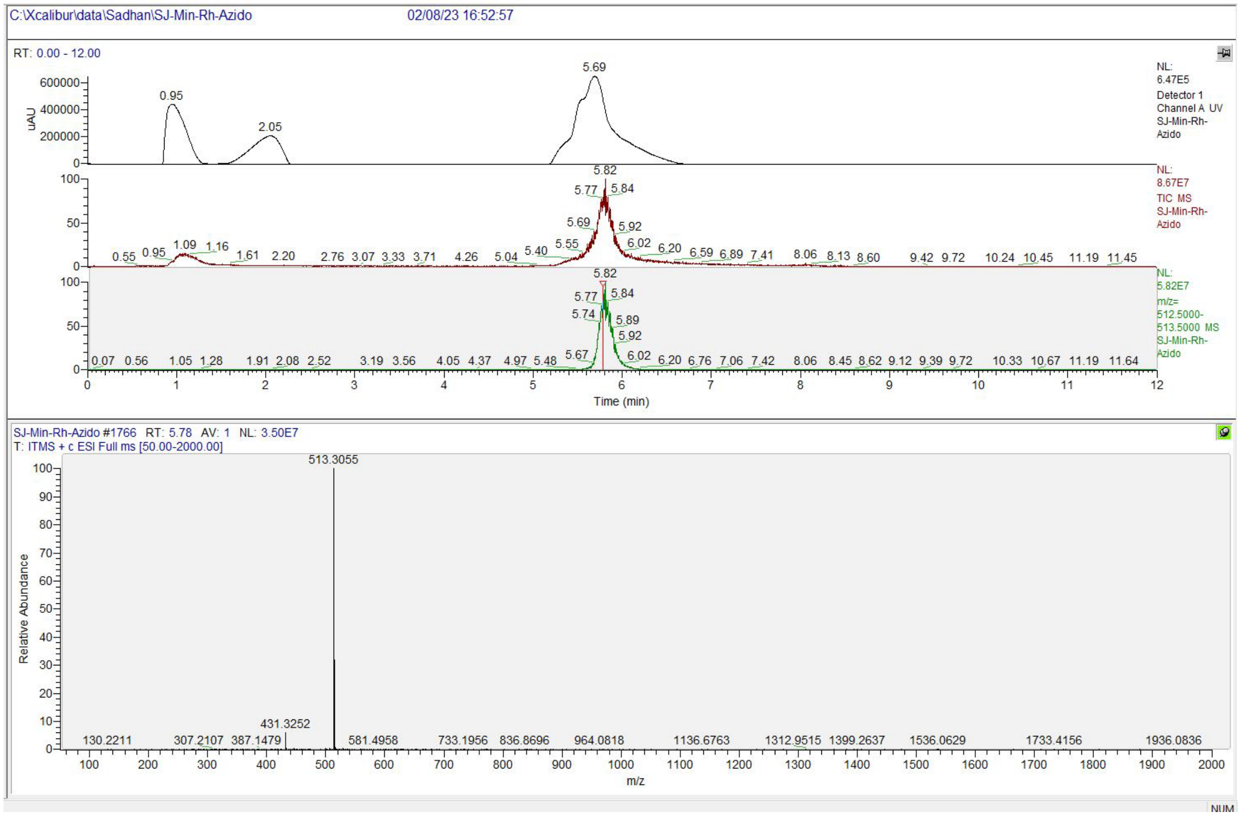

C:\Xcalibur\data\Sadhan\SJ-Min-Rh-Azido
02/08/23 16:52:57
RT: 0.00 - 12.00
NL: 6.47E5 Detector 1 Channel A UV SJ-Min-Rh-Azido
NL: 8.67E7 TIC MS SJ-Min-Rh-Azido
NL: 5.82E7 m/z= 512.5000-513.5000 MS SJ-Min-Rh-Azido
uAU
Time (min)
SJ-Min-Rh-Azido #1766 RT: 5.78 AV: 1 NL: 3.50E7
T: ITMS + c ESI Full ms [50.00-2000.00]
Relative Abundance
513.3055
431.3252
m/z

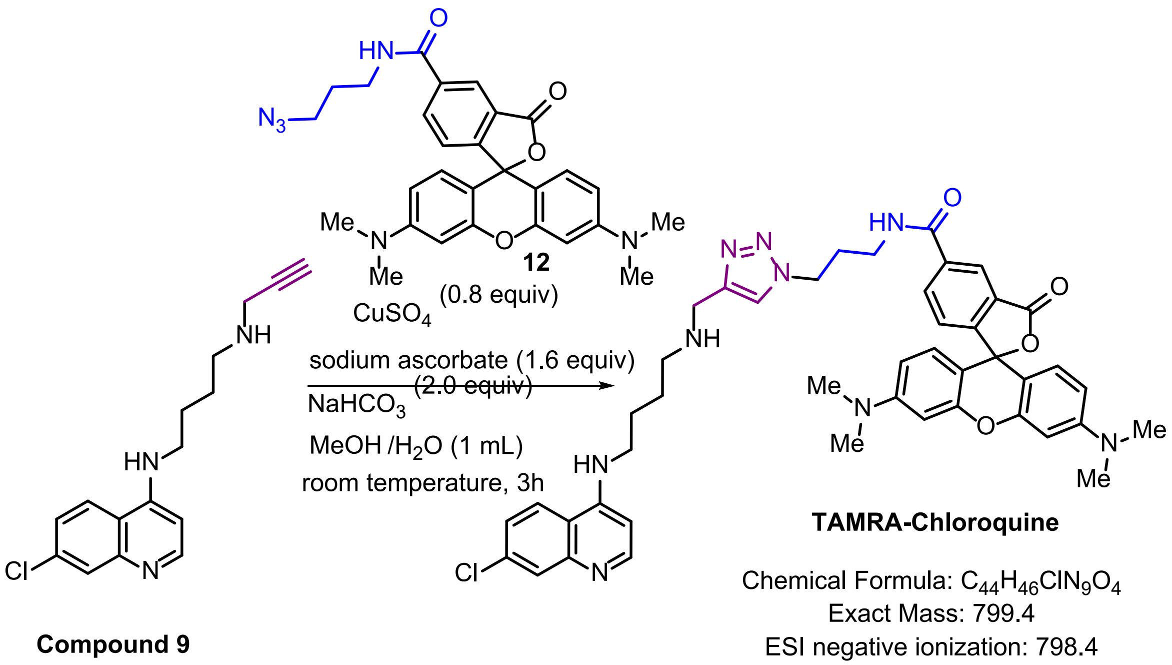

Synthesis of TAMRA-Chloroquine Probe

Procedure: *N*1-(7-chloroquinolin-4-yl)-N4-(prop-2-yn-1-yl)butane-1,4-diamine (1.0 equiv, 0.0072 mmol, 2.0 mg), *N*-(3-azidopropyl)-3',6'-bis(dimethylamino)-3-oxo-3H-spiro[isobenzofuran-1,9'-xanthene]-5-carboxamide (1.2 equiv, 0.06 mmol, 3.0 mg), CuSO4, 5 H_2O (0.8 equiv, 0.0048 mmol, 1.2mg), sodium ascorbate (1.6 equiv. 0.0096 mmol, 2.0 mg) and $NaHCO_3$(0.05 mmol, 1.2 mg) were added in Water:MeOH (1:1) solvent mixture (2 mL) and stirred for 3-6 h at room temperature. The reaction was monitored by LCMS analysis. After the reaction was completed, the products were isolated by HPLC water (0.1% TFA) as solvent A and acetonitrile (0.1% TFA) as solvent B. The flow at a rate of 10 mL/min. The purification program was run as the following: 0 to 20.0% solvent B for the first 3 min time, followed by a liner progression to 30% solvent B for next 27 minutes, end with the last 10 minutes of 80% solvent B and more 10 minutes of 100% solvent A for a total of 30 minutes per HPLC run. The product peak came out at 13.5 min, and lyophilization of the collected fraction as pink semi-solid.

Physical State: pink smi-solid

Yield: 42% (1.8 mg isolated)

1H NMR (500 MHz, CD3CN) δ 8.37-8.30 (m, 2H), 8.20 (t, *J* = 7.46, 2H), 7.99 (d, *J* = 7.9 Hz, 2H), 7.65-7.58 (m, 2H), 7.13-7.08 (m, 2H), 6.98-6.93 (m, 2H), 6.88-6.84 (m, 1H), 6.72-6.68 (m, 2H), 6.24 (d, *J* = 6.9 Hz, 2H), 6.09 (s, 1H), 5.38 (t, *J* =4.39, 1H), 4.70 (t, J = 7.06 Hz, 1 H), 4.55 (s, 1H), 4.41 (*t*, J =6.96 Hz, 1H), 4.37-4.34 (m, 1H) 4.22 (s, 1H), 4.15-4.13 (m. 1H), 4.01 (s, 2H), 3.17 (s, 12H), 2.29- 2.25 (m, 1H), 2.22 (s, 1H), 2.17 (q, J= 4.25 Hz, 1H), 1.58-1.55 (m, 1H), 1.47 (t, *J*= 2.25 Hz, 1H)

LCMS (ESI (negative): [M-H] calcd for C44H45ClN9O4: 798.4; found, 798.5.

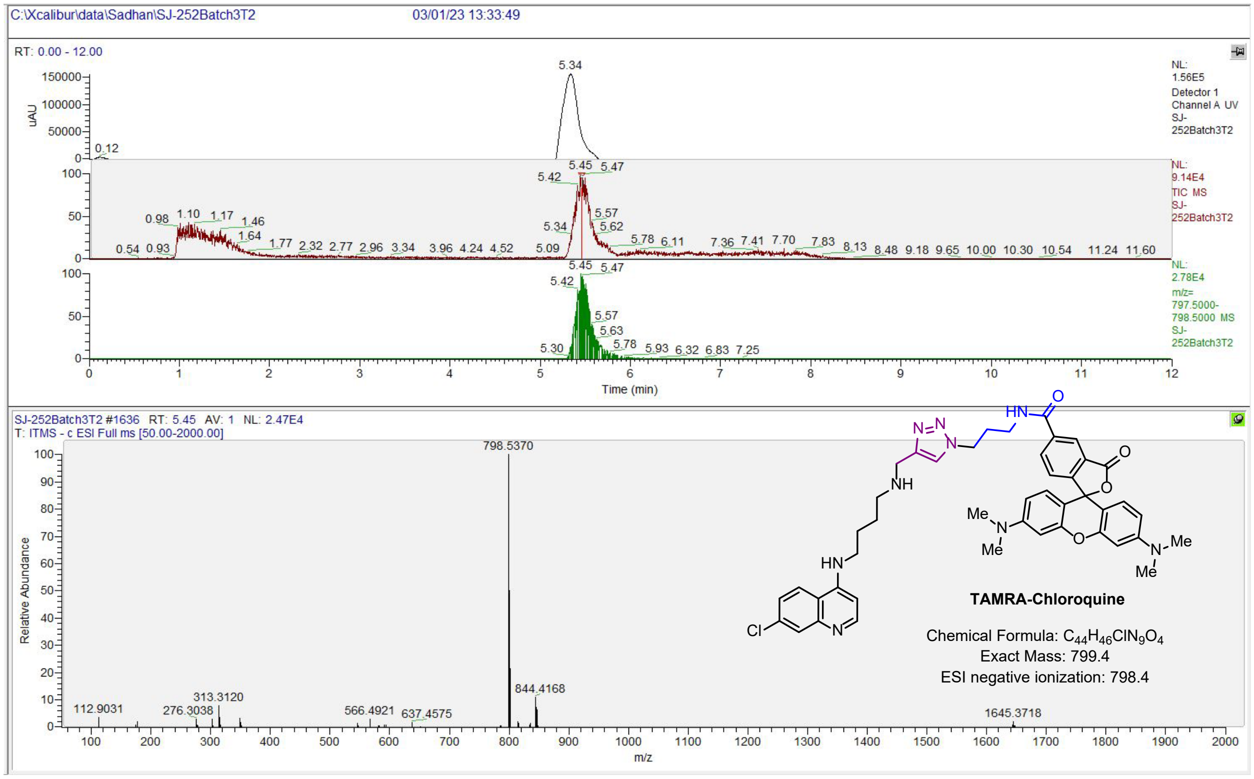

HPLC purification:

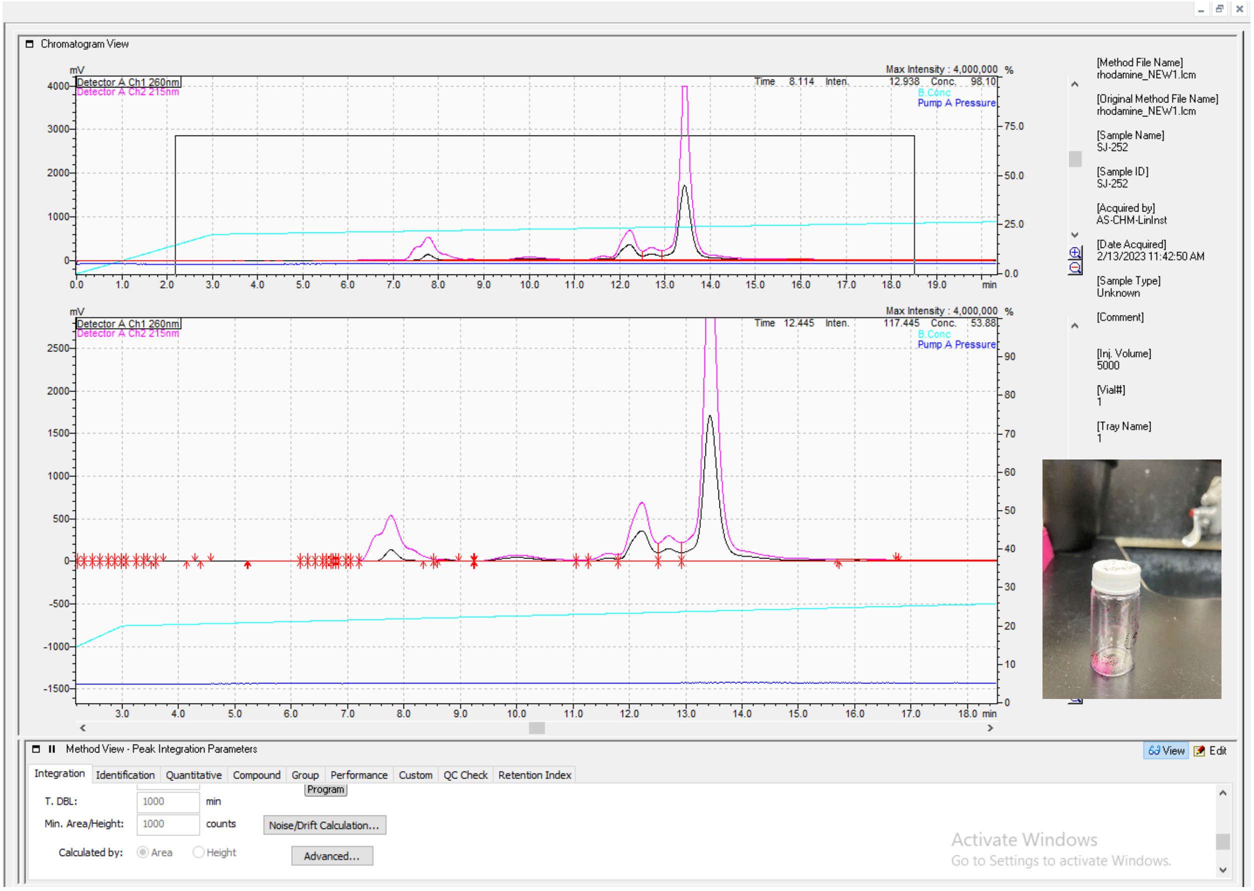
Chromatogram View
Detector A Ch1 260nm
Detector A Ch2 215nm
Max Intensity : 4,000,000
Time 8.114 Inten. 12.938 Conc. 98.10
B Conc
Pump A Pressure
Time 12.445 Inten. 117.445 Conc. 53.88
[Method File Name]
rhodamine_NEW1.lcm
[Original Method File Name]
rhodamine_NEW1.lcm
[Sample Name]
SJ-252
[Sample ID]
SJ-252
[Acquired by]
AS-CHM-LinInst
[Date Acquired]
2/13/2023 11:42:50 AM
[Sample Type]
Unknown
[Comment]
[Inj. Volume]
5000
[Vial#]
1
[Tray Name]
1
Method View - Peak Integration Parameters
Integration
Identification
Quantitative
Compound
Group
Performance
Custom
QC Check
Retention Index
Program
T. DBL: 1000 min
Min. Area/Height: 1000 counts
Noise/Drift Calculation...
Calculated by: Area Height
Advanced...
Activate Windows
Go to Settings to activate Windows.
View
Edit

^{1}H NMR

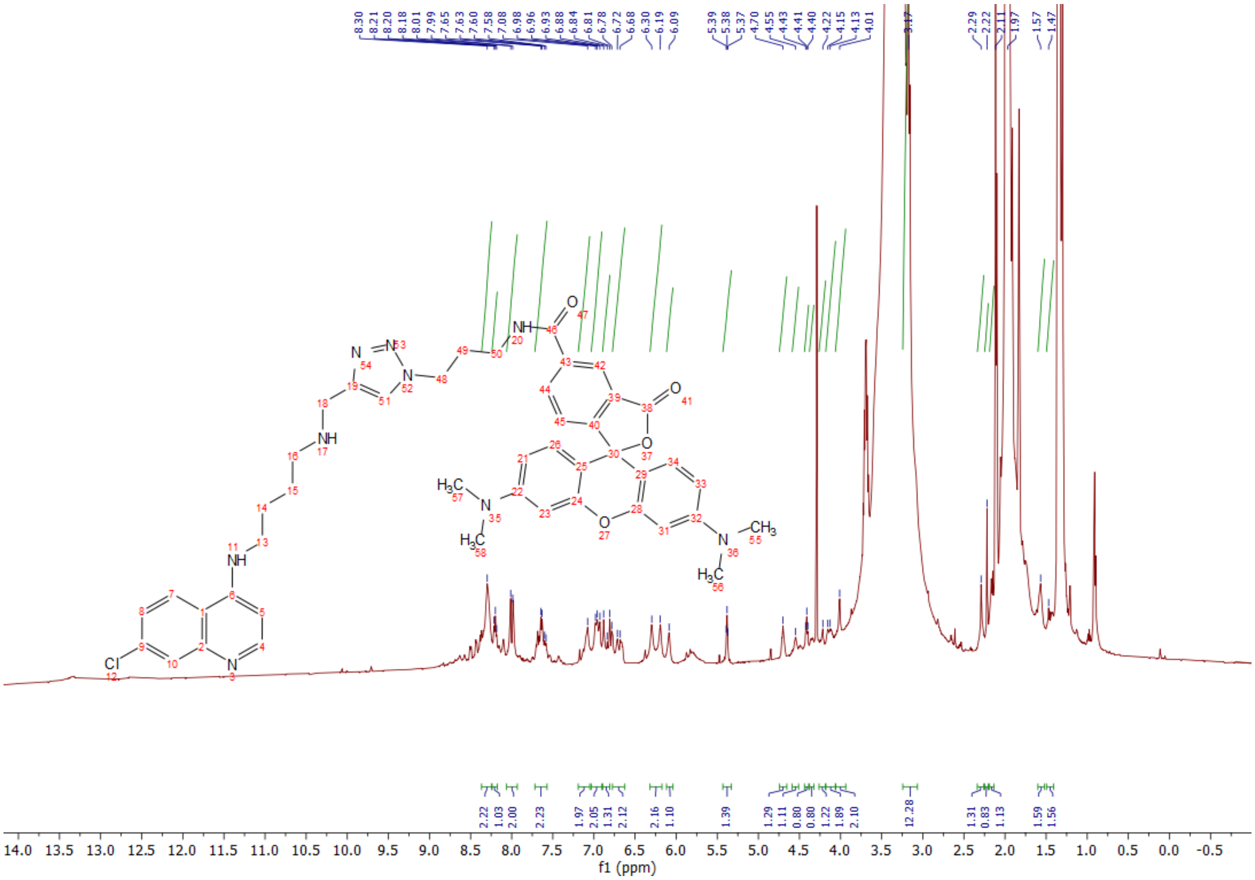

Supplement: Supplementary file 1 — oc2c01325_si_001.pdf [file oc2c01325_si_001.pdf]
