# Supplementary material for: A Turn-On Fluorescent Amino Acid Sensor Reveals Chloroquine’s Effect on Cellular Amino Acids via Inhibiting Cathepsin L
Source: ACS Cent Sci. 2023 Apr 24;9(5):980–91. doi: 10.1021/acscentsci.2c01325 (PMC10214525; doi:10.1021/acscentsci.2c01325)
Supplement: Supplementary file 3 — oc2c01325_si_003.pdf [file oc2c01325_si_003.pdf]

oc-2022-013254.R1

Name: Peer Review Information for "A turn-on fluorescent amino acid sensor reveals chloroquine's effect on cellular amino acids via inhibiting cathepsin L"

First Round of Reviewer Comments

Reviewer: 1

Comments to the Author

In "A turn-on fluorescent amino acid sensor reveals chloroquine's effect on cellular amino acids via inhibiting cathepsin L" by M. R. Smith, L. Zhang, Y. Jin, M. Yang, A. Bade, K. Gillis, T. E. Glass, and H. Lin submitted to ACS Central Science as a Research Article, the authors describe the development of an amino acid turn-on fluorogenic probe called NS560. After validation that the probe reports on amino acids in live cells, they used the probe to monitor changes in amino acid pools after treatment with chloroquine. Using a photo-crosslinking derivative of chloroquine and following up with knockdown studies they determined that inhibition of CTSL by chloroquine is a major contributor to amino acid accumulation that was observed. Additionally, the authors relate their observations to a timely topic of SARS-CoV-2 infection and treatment with chloroquine. This manuscript appears to be scientifically sound and significant but could use some additional experiments (see comments below). Therefore, I recommend acceptance of this manuscript for publication after revisions to address my comments below.

Major questions and comments:

1. Page 6, Figure 1A, How is the pinacol ester removed before reaction with an amino acid? Is there evidence that this occurs first as drawn?

2. Page 6, Figure 1C/Overall question: All of the natural amino acids plus GABA are tested to see if there is a response with this probe. Does the probe have a fluorescent response to the N-terminus of proteins including small peptides like glutathione?

3. Page 6, Figure 1A, Chris Chang's group at Berkley and others have used aryl boronic acid pinacol esters in probes for hydrogen peroxide. Does the fluorescence of your probe change in the presence of hydrogen peroxide?

4. Page 15, line 50, it is stated that cloroquine could become protonated and trapped in the lysosome, could your probe also be protonated and concentrated in the lysosome resulting in the observed increase in signal just being from additional probe being present and not an increase in amino acid concentration?

5. SI page 33, NMR spectra of NS560, there are unannotated peaks at ~0.1, 1.3, and 1.7 ppm in the 1H NMR and unannotated peaks at ~30 ppm and 0 ppm in the 13C NMR. Pure compounds are necessary for

use in biological systems as impurities can interfere with the biological process or be the cause of the observed result. What is the identity of these unannotated peaks and the purity of NS560?

6. Full chemical characterization of CQ-X is missing (13C NMR and HRMS) additionally the NMR spectra should be included to allow reviewers to confirm assignments and assess purity.

Minor questions and comments:

1. On page 4, line 53-57 it is stated that the rotational restriction of the aryl boronic acid from formation of the macrocycle influences the fluorescence properties. Has this been previously reported in similar frameworks? If so, these reports should be cited.

2. Page 7 Figure 2C and D, the side panels should have scale bars

3. To increase the reproducibility of microscopy experiments reported in the literature, all replicates and images used in the analysis should be included as supplemental information. This should include images used to generate figure 3C, 5G, and 5E.

Reviewer: 2

Comments to the Author

Lin and colleagues developed a fluorescence probe to detect free amino acids (AAs) in living cells. When they used this probe to screen small molecules that can perturb the level of AAs, they found that chloroquine (CQ) could cause accumulations of AAs in lysosome and late endosome. By applying a photo-affinity CQ probe with chemical proteomics experiments, they identified a cysteine protease, CTSL, as one major target that is highly relevant with CQ's biological activity. Genetic and biochemical validation of CTSL was performed and the results showed that CQ could inhibit the CTSL's activity which led to accumulation of AAs in lysosome. Overall, this is an interesting study with both fluorescent probe development and target deconvolution elements. Intriguing biological insights have been obtained by exploring mechanistic action of CQ. Target deconvolution using the photo-affinity probe is routine and the results are solid. The reviewer feels the major missing part is how CQ inhibits CTSL mechanistically. The work could be improved with a few more experiments:

1) Were the authors able identify the crosslinking peptides in CTSL so that a potential binding site could be inferred?

2)If no experimental complex structure is available, a computational docking study would be helpful to provide more clues.

3) How tight is the binding between CQ and CTSL? With purified protein in hand, this can be easily measured by ITC or SPR.

4)Since CTSL is cysteine protease, can authors comment on whether the inhibition might be due to covalent modification of the active-site cysteine?

Author's Response to Peer Review Comments:

Dear Editor,

We appreciate the opportunity to submit a revised version of the manuscript. We have addressed all the editorial and reviewers' comment as detailed in the attached point-by-point response. We hope you and the reviewers will find the revision satisfactory.

Because the revision required new experimental data carried out by other people not previously listed as authors, we have to add two more people as authors.

Best regards,

Hening

We would like to thank the editor and reviewers for the helpful comments. We have addressed all the comments as detailed below. Editor and Reviewers' comments are shown in black fonts, while our responses are shown in blue fonts.

To address the reviewers' concerns, we need to carry out new experiments, including the synthesis of a fluorescence probe. The chemist and one of the authors of the manuscript that did the chloroquine related synthesis, Dr. Min Yang, unfortunately passed away in January 2023. We thus had to recruit other people to complete the experiments. This explains why we were a little delayed and why we are adding additional authors who contributed to the revision.

SI FILE: SI should be formatted with a cover sheet listing authors, manuscript title, and the number of pages, figures, and tables. Please also include the email address of the corresponding author on the first page of the Supporting Information, with an asterisk next to their name in the author list.

The supplementary information file has been reformatted to include a cover sheet with the required information.

SI PAGINATION: SI pages must be numbered consecutively, starting with page S1.

The supplemental information file has been updated to include page numbers.

TABLE S1: Please move the title for Table S1 to appear above the table, not below it like a figure.

Table S1 title has been moved above the table.

SI STATEMENT: Because your manuscript is accompanied by Supporting Information for publication, a brief description of the supplementary material is required in the manuscript. The appropriate format is: Supporting Information (header), followed by a brief statement in nonsentence format listing the contents of the material supplied as Supporting Information.

A description of the contents of the supplementary information has been included.

REFERENCES: Please reformat your references according to journal guidelines. Periodical references should contain authors' surnames followed by initials, article title, journal abbreviation, year, volume number, and complete page range. For more information, please visit the author guidelines: http://pubs.acs.org/paragonplus/submission/acscii/acscii_authguide.pdf

The references have been updated to reflect the preferred journal format.

TOC GRAPHIC: ACS Central Science requests a Table of Contents (TOC) Graphic. Please provide the TOC graphic as the last page of the submitted manuscript. The graphic requested for the TOC entry can be in the form of a structure, graph, drawing, SEM/TEM photograph, or reaction scheme. Please submit a graphic in the actual size to be used for the Table of Contents, that is 1.375 inches high x 3.25 inches (20 picas) wide. The type size of labels, formulas, or numbers within the graphic must be legible. Tables or spectra are not acceptable. Please label this page "For Table of Contents Use Only" and include the manuscript title and author names. If you upload

your TOC graphic separately, please upload it as a 'Graphic for the Manuscript' file. Please note that ACS only accepts .tif and .pdf files for graphics.

A TOC graphic has been created and added to the end of the manuscript and that page has been labelled accordingly.

SYNOPSIS: ACS Central Science requires a brief synopsis. The synopsis should be no more than 200 characters (including spaces) and should reasonably correlate with the Table of Contents (TOC) graphic. The synopsis is intended to explain the importance of the article to a broader readership across the sciences. Please place your synopsis in the manuscript file after the TOC graphic.

A brief synopsis was added to the manuscript file after the TOC graphic.

Reviewer: 1

Recommendation: Publish in ACS Central Science after minor revisions noted.

Comments:
In "A turn-on fluorescent amino acid sensor reveals chloroquine's effect on cellular amino acids via inhibiting cathepsin L" by M. R. Smith, L. Zhang, Y. Jin, M. Yang, A. Bade, K. Gillis, T. E. Glass, and H. Lin submitted to ACS Central Science as a Research Article, the authors describe the development of an amino acid turn-on fluorogenic probe called NS560. After validation that the probe reports on amino acids in live cells, they used the probe to monitor changes in amino acid pools after treatment with chloroquine. Using a photo-crosslinking derivative of chloroquine and following up with knockdown studies they determined that inhibition of CTSL by chloroquine is a major contributor to amino acid accumulation that was observed. Additionally, the authors relate their observations to a timely topic of SARS-CoV-2 infection and treatment with chloroquine. This manuscript appears to be scientifically sound and significant but could use some additional experiments (see comments below). Therefore, I recommend acceptance of this manuscript for publication after revisions to address my comments below.

Major questions and comments:
1. Page 6, Figure 1A, How is the pinacol ester removed before reaction with an amino acid? Is there evidence that this occurs first as drawn?

The following study shows evidence of pinacol ester removed at physiological pH with strong kinetics. We added this citation into the main text.

Achilli, C.; Ciana, A.; Fagnoni, M.; Balduini, C.; Minetti, G. "Susceptibility to hydrolysis of phenylboronic pinacol esters at physiological pH" Cent. Eur. J. Chem.,11(2) **2013** 137-139.

2. Page 6, Figure 1C/Overall question: All of the natural amino acids plus GABA are tested to see if there is a response with this probe. Does the probe have a fluorescent response to the N-terminus of proteins including small peptides like glutathione?

In order to address this question, we titrated NS560 with glutathione at both pH 7.4 and 5.0. There is strong binding and fluorescence turn-on comparable to other proteinogenic amino acids. The new data has been added as supplemental Figures S43 and S44 and supplement Table S1.

3. Page 6, Figure 1A, Chris Chang's group at Berkley and others have used aryl boronic acid pinacol esters in probes for hydrogen peroxide. Does the fluorescence of your probe change in the presence of hydrogen peroxide?

High levels of hydrogen peroxide will degrade NS560. Thus, we do not anticipate our sensor to sense H_2O_2.

4. Page 15, line 50, it is stated that chloroquine could become protonated and trapped in the lysosome, could your probe also be protonated and concentrated in the lysosome resulting in the observed increase in signal just being from additional probe being present and not an increase in amino acid concentration?

The NS560 sensor itself is membrane permeable even at acidic pH of the lysosomes. It would only accumulate if bound to an amino acid inside the lysosomes. Incubation of living cells with NS560 under basal settings shows fluorescent signal with only occasional puncta at lysosomes (see Figure 2 or Figure 3A). The data suggest that NS560 does not get trapped in the lysosome like chloroquine.

5. SI page 33, NMR spectra of NS560, there are unannotated peaks at ~0.1, 1.3, and 1.7 ppm in the 1H NMR and unannotated peaks at ~30 ppm and 0 ppm in the 13C NMR. Pure compounds are necessary for use in biological systems as impurities can interfere with the biological process or be the cause of the observed result. What is the identity of these unannotated peaks and the purity of NS560?

We have replaced the old NMR with a new one where TMS was not used (peak at 0 ppm) without the peak at 30 ppm (which was a solvent impurity). The new data can be found in the Synthetic Procedures.

6. Full chemical characterization of CQ-X is missing (13C NMR and HRMS) additionally the NMR spectra should be included to allow reviewers to confirm assignments and assess purity.

We have added 13C NMR and HRMS for CQ-X in the Synthetic Procedures section.

Minor questions and comments:

1. On page 4, line 53-57 it is stated that the rotational restriction of the aryl boronic acid from formation of the macrocycle influences the fluorescence properties. Has this been previously reported in similar frameworks? If so, these reports should be cited.

Although there is not an exact precedent, we have added the following citation to the main text to support our proposal.

Hettie, K. S.; and Glass, T. E. "Coumarin-3-Aldehyde as a Scaffold for the Design of Tunable PET- 5 Modulated Fluorescent Sensors for Neurotransmitters" *Chem. Eur. J.* **2014**, *20*, 17488-17499.

2. Page 7 Figure 2C and D, the side panels should have scale bars

Scale bars have been added to these figures.

3. To increase the reproducibility of microscopy experiments reported in the literature, all replicates and images used in the analysis should be included as supplemental information. This should include images used to generate figure 3C, 5G, and 5E.

We thank the reviewer for the interest in reproducibility of microscopy experiments. However, a large number of cells were imaged for each analysis. If we placed each file into the supplemental, it would require a massive file size and is not consistent with typical publication practice. However, we have added Supplemental Figures S52-54 with representative images and statistical analysis for the other biological replicate experiments from Figures 3C, 5G, and 5E where we quantified NS560 puncta. All data was again analyzed using Fiji/ImageJ software.

Reviewer: 2

Recommendation: Publish in ACS Central Science after minor revisions noted.

Comments:
Lin and colleagues developed a fluorescence probe to detect free amino acids (AAs) in living cells. When they used this probe to screen small molecules that can perturb the level of AAs, they found that chloroquine (CQ) could cause accumulations of AAs in lysosome and late endosome. By applying a photo-affinity CQ probe with chemical proteomics experiments, they identified a cysteine protease, CTSL, as one major target that is highly relevant with CQ's biological activity. Genetic and biochemical validation of CTSL was performed and the results showed that CQ could inhibit the CTSL's activity which led to accumulation of AAs in lysosome. Overall, this is an interesting study with both fluorescent probe development and target deconvolution elements. Intriguing biological insights have been obtained by exploring mechanistic action of CQ. Target deconvolution using the photo-affinity probe is routine and the results are solid. The reviewer feels the major missing part is how CQ inhibits CTSL mechanistically. The work could be improved with a few more experiments:

1) Were the authors able identify the crosslinking peptides in CTSL so that a potential binding site could be inferred?

We identified the following peptides, all from the mature form of CTSL in the C-terminal region (shown in order N- to C-terminal):
AVATVGPISVAIDAGHESFLFYK
NSWGEEWGMGGYVK
NHCGIASAASYPTV

However, the crosslinked peptides were not identified because our experiments were not designed to detect the crosslinked peptides. In fact, the crosslinked peptides will remain bound to the streptavidin beads and will not be eluted. In the MS search, we also did not search for the modified peptides. The modified peptide would contain both CQ and biotin and the modification is too large for the search.

Based on the published structure of CTSL (PDB 2XU3), all three identified peptides are close to the catalytic triad Asn-His-Cys. Furthermore, the size of the CTSL band detected when validating the proteomics was the fully processed, mature CTSL of ~25 kDa. Thus, it is unlikely that CQ is binding the amino acids that make up the pro-peptide N-terminal region. Computational docking showed that CQ occupies the active site of CTSL, which we have added as Supplemental Figure S51.

2)If no experimental complex structure is available, a computational docking study would be helpful to provide more clues.

We performed computation docking study and attached the findings in Supplemental Figure S51. The best docking model shows that CQ is docked into the active site.

3) How tight is the binding between CQ and CTSL? With purified protein in hand, this can be easily measured by ITC or SPR.

The difficulty with ITC or SPR is the requirement of a large amount of CTSL. We can only get CTSL with a stock solution of ~ 2 μM. In addition, CTSL is very unstable. At room temperature, our purified CTSL will degrade itself within one hour. This instability and difficulty associated with getting large amount of proteins limits us from doing such affinity measurement. In our original manuscript, we measured the IC_{50} value of CQ inhibiting CTSL to be ~200 μM. We also used the intrinsic fluorescence change to show the direct binding to CQ to CTSL. However, due to the amount of protein required and other problems, we cannot obtain the K_d from the intrinsic fluorescence measurement. To measure the binding affinity, during the revision, we designed a fluorescent probe, Chloroquine-TAMRA (CQ-TAMRA), to perform a fluorescence polarization assay. We were able to obtain a milipolarization (mP) shift of 20-25, again indicating that CTSL does bind to CQ. However, we are again limited by the stability and amount of the CTSL and thus, could not saturate the binding curve to get a reliable K_d.

However, given that the docking suggests that CQ bind to the CTSL active site and thus should be competitive with substrate, we can calculate the *Ki* (which is equivalent to K_d) to be around 35 μM using the equation of $K_i = IC_{50}/(1+[S]/K_m)$. This estimate is now included in the manuscript.

4)Since CTSL is cysteine protease, can authors comment on whether the inhibition might be due to covalent modification of the active-site cysteine?

We thank the reviewer for this interesting question. Because covalent inhibitors require time to react with target proteins and thus the inhibition is typically dependent on pre-incubation time with the inhibitors. For the covalent inhibitor E64d, the percent activity of CTSL changes from 17% with pre-incubation to 66% without preincubation, which is consistent with the covalent inhibition nature. In contrast, pre-incubation of CTSL with CQ does not impact the inhibitory effect of CQ on the enzyme. Thus, we conclude that CQ is not a covalent inhibitor. This data has been added to the Supplemental Figure S50.

oc-2022-013254.R2

Name: Peer Review Information for "A turn-on fluorescent amino acid sensor reveals chloroquine's effect on cellular amino acids via inhibiting cathepsin L"

Second Round of Reviewer Comments

Reviewer: 2

Comments to the Author

The authors have done a good job to address my questions and concerns. I support the publication of the manuscript in the current form.

Reviewer: 1

Comments to the Author

Reviewer 1 comments

Major questions and comments:

1. Okay

2. In this case does the probe actually report on free amino acids or can it report on the prescence of any short peptide?

3. Okay

4. Okay

5. This doesn't appear to be corrected, the 1H NMR still has the unannoated peaks on page S39.

6. Thank you for including this, the 1H NMR has unnannoated peaks at ~2.0 ppm and ~1.25 ppm as well as 13C NMR at 20 ppm. Pure compounds are necessary for use in biological systems as impurities can interfere with the biological process being studied or be the cause of the result. What is the identity of these unannotated peaks and the purity of CQ-X?

Minor questions and comments:

1. okay

2. okay

3. Okay thank you. I also notice that this work was supported by the NIH, the new Data Management and Sharing Policy would typically require this data being publically avialable. While the awards that funded this work may not be bound by this new policy, the authors may want to consider depositing data

included in the analysis presented here into a repository where it can be accessed such as their institution's digital repository: eCommons: Cornell's Digital Repository.

Author's Response to Peer Review Comments:

Dear Editor,

Thanks for allowing us to submit an revised version of the manuscript. We have addressed Reviewer 1's remaining concerns as detailed in the attached point-by-point response. While we did carry out one new experiment to answer the reviewer's question, we only show the results in the attached point-by-point response as we believe it is not very relevant (the reviewer's question may be due to an misunderstanding of the structure of glutathione). The only change we made to the manuscript is in the Supporting information, Page 39, where replaced the 13C NMR spectrum with one with much better resolution.

I hope you will find the revision satisfactory and suitable for publication.

Best regards,

Hening

Review 2 has recommended publication without change. We thank Reviewer 2 for the positive comments.

Reviewer 1 still raised a few more comments based on our previous response and we have addressed them as detailed below. The number in front of the comments are from the last revision. The reviewer's comments are shown in black fonts, while our responses are shown in blue fonts.

Major comments

2. In this case does the probe actually report on free amino acids or can it report on the presence of any short peptide?

The reason that the probe can report on GSH is because of the isopeptide bond in the structure. As a result, GSH has an α-amino acid structure just like free amino acids (see the structure highlighted in the red rectangle in Figure 1 below).

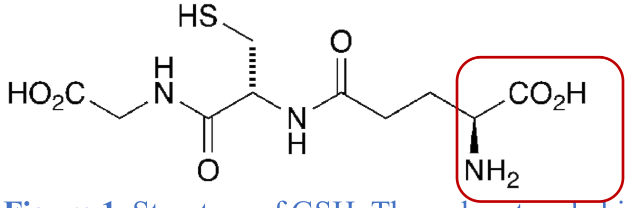

Figure 1. Structure of GSH. The red rectangle highlights the α-amino acid moiety that can readily react with NS560 to increase fluorescence.

Normal peptides do not have the α-amino acid moiety and thus the probe does not report on it, or at least report very poorly. To demonstrate this, we used a Gly-Gly-His peptide and measured the fluorescence change (Figure 2). While there is some increase in the fluorescence, the fold increase is very small (similar to some of the worst amino acids such as cysteine) and the maximum emission wavelength is also different (520 nm instead of the 560 nm for amino acids). Therefore, NS560 primarily report on α-amino acid structures.

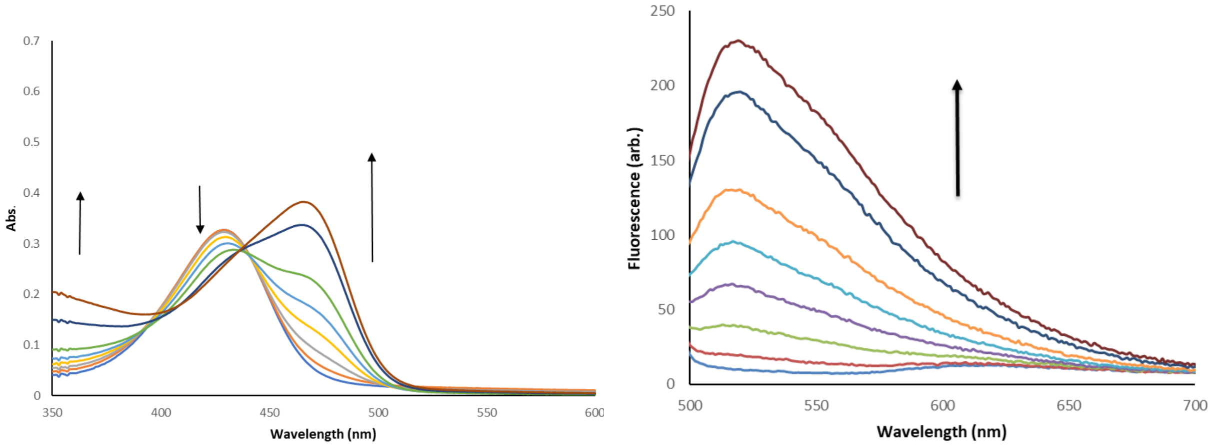

Figure 2. UV/Vis and fluorescence spectroscopy of **NS560** (10 μM in 25 HEPES, 50 mM $Na_2S_2O_3$, 1% DMSO, pH 5) with Gly-Gly-His (180 mM in buffer) , λ_{ex} = 488 nm, λ_{em} = 520 nm.

5. This doesn't appear to be corrected, the 1H NMR still has the unannotated peaks on page S39.

6. Thank you for including this, the 1H NMR has unannotated peaks at ~2.0 ppm and ~1.25 ppm as well as 13C NMR at 20 ppm. Pure compounds are necessary for use in biological systems as impurities can interfere with the biological process being studied or be the cause of the result. What is the identity of these unannotated peaks and the purity of CQ-X?

Response to 5 and 6: Thank you very much for making this comment and suggesting the explanation. Yes, we agree that there are two unannotated peaks in ^{1}H NMR of CQ-X on page S39. The peak at ~ 2.0 ppm belongs to solvent acetone proton, likely due to acetone wash of the NMR tube. The peak at ~ 1.25 ppm shows hexane/grease. These impurities come from either solvent or pipetting deuterated solvent by plastic pipette tip or plastic syringe. The same explanation applies for the peak at ~20 ppm for ^{13}C NMR (13C is low sensitivity due to low natural abundance than 1H). Hence, the unannotated peaks have come from solvent impurities (acetone and hexane/grease) in NMR spectra. We are confident the purity of the final compounds used are high (>98%).

Minor comment 3. Okay thank you. I also notice that this work was supported by the NIH, the new Data Management and Sharing Policy would typically require this data being publically available. While the awards that funded this work may not be bound by this new policy, the authors may want to consider depositing data included in the analysis presented here into a repository where it can be accessed such as their institution's digital repository: eCommons: Cornell's Digital Repository.

We will submit our data here.
